# Supplementary material for: Protocol for the process evaluation of an intervention to improve antenatal smoking cessation support (MOHMQuit) in maternity services in New South Wales, Australia
Source: BMJ Open. 2024 Mar 19;14(3):e081208. doi: 10.1136/bmjopen-2023-081208 (PMC10952879; doi:10.1136/bmjopen-2023-081208)
Supplement: Supplementary data [file bmjopen-2023-081208supp001.pdf]

Supplementary Table 1: Implementation outcomes, definitions, strategies for maximising implementation outcomes, frameworks used and measurement items

Implementation outcome (abbreviated definition)	Strategies used to maximise implementation outcomes	Level of analysis	Frameworks used	Data collection instruments, timing, participants	Items from instruments
Adoption/Uptake (intention or action to try to employ MOHMQuit)	• Warm-up meetings<br>• Follow up meetings<br>• Community of Practice	Site level<br>Individual clinician level	Proctor[1]<br>RE-AIM[2]<br>(Adoption)	• Warm-up and follow up meetings<br>• 3 months following training - questionnaire for leaders<br>• 6 months following training - questionnaire with clinicians<br>• 6 months following training - semi-structured interview with leaders<br>• Community of Practice peer support meetings attendance data	Meetings<br>* Whether the 10 and 2-week warm-up meetings took place/were attended;<br>* Whether the 2 and 4 week post training meetings took place/were attended<br>3 month questionnaire for leaders<br>** In the last 3 months did you or any other staff in your service…* (followed by a number of MOHMQuit components)*?*<br>6 month questionnaire for clinicians<br>** How useful were each of the MOHMQuit resources when working with women (scale of 1-3 Very useful to Not at all useful + Not Applicable as a response option)?*<br>6 month interview guide for leaders<br>** Have you, or any of your colleagues implemented any of the components of MOHMQuit* (followed by a number of MOHMQuit components)*?*<br>** What do you think helped in delivering MOHMQuit?*<br><br>Community of Practice meetings<br>* Sites attending community of practice meetings
Fidelity (delivered as intended in the Protocol[3], adherence)	• Warm-up and follow-up meetings<br>• Consistency in the team delivering MOHMQuit training at each site in the first instance	Site level	Proctor[1]<br>RE-AIM[2]<br>(Implementation)	• Warm-up and follow up meetings<br>• Training logs of expected and actual	Meetings<br>* Whether the 10 and 2-week warm-up meetings took place/were attended;<br>* Whether the 2 and 4 week post training meetings took place/were attended<br><br>Training logs

Implementation outcome (abbreviated definition)	Strategies used to maximise implementation outcomes	Level of analysis	Frameworks used	Data collection instruments, timing, participants	Items from instruments
	• clear plans and materials for content of training			attendance at training of leaders and clinicians recorded at the time of training<br>• Fidelity record (a checklist of which aspects of the planned training were actually delivered – completed by researchers observing the training, plus any additional observational data about engagement of participants)<br>• 6 months following training - semi-structured interview with leaders	* Proportion of eligible leaders and clinicians who were invited and who actually attended training<br><br>Fidelity record<br>* the extent to which training was delivered as anticipated<br><br>6 month interview guide for leaders<br>** Were any adaptations made to MOHMQuit? (What/who/when/why/how?)*
Penetration (degree of integration of MOHMQuit practices within the service)	• Involving leaders in the training for clinicians for a whole-of-service approach<br>• MOHMQuit leadership components which focus on repeated audit and feedback plus action planning;	Site level	Proctor[1]<br>RE-AIM[2]<br>(Adoption)	• 3 months following training - questionnaire for leaders<br>• 6 months following training - semi-structured interview with leaders	3 month questionnaire for leaders<br>** In the last 3 months did you or any other staff in your service…* (followed by a number of MOHMQuit components)*?*<br>6 month interview guide for leaders<br>** Have you, or any of your colleagues implemented any of the components of MOHMQuit* (followed by a number of MOHMQuit components)*?*<br>** How much has MOHMQuit become part of 'usual practice'? (e.g. standard operating procedures, local policies, SCS as a*

Implementation outcome (abbreviated definition)	Strategies used to maximise implementation outcomes	Level of analysis	Frameworks used	Data collection instruments, timing, participants	Items from instruments
	developing and implementing a clinical pathway for SCS; and the development and maintenance of SCS 'champions' within each service<br>• The train the trainer model as an integral part of the intervention to support its ongoing implementation				***standing item on meeting agendas, audit part of usual audit schedule etc.)***
**Reach (did MOHMQuit include all clinicians and leaders that it aimed to?)**	• **10-week warm-up meetings to allow time for planning and rostering**<br>• **The train the trainer model as an integral part of the intervention to support participation of all relevant existing and new staff**	**Site level**	**RE-AIM[4]**	• **Training logs of expected and actual attendance at training of leaders and clinicians recorded at the time of training**<br>• **3 months following training - questionnaire for leaders**<br>• **6 months following training - semi-**	**Training logs**<br>*** Proportion of eligible leaders and clinicians who were invited and proportion who actually attended training (compare the seniority, and role e.g. midwife, obstetrician of those who participated to those who did not)**<br><br>**3 month questionnaire for leaders**<br>*** *In the last 3 months did you or any other staff in your service design and run any staff training on SCS?* (the train the trainer model);**<br>*** *Please tell us more about this training* (space to write a qualitative response)**<br>**6 month interview guide for leaders**

**Implementation outcome (abbreviated definition)**	**Strategies used to maximise implementation outcomes**	**Level of analysis**	**Frameworks used**	**Data collection instruments, timing, participants**	**Items from instruments**
				structured interview with leaders	*** Have you, or any of your colleagues implemented any of the components of MOHMQuit** (followed by a number of MOHMQuit components including **Designing and running any staff training**)?
**Sustainability (whether factors are in place to promote the ongoing use of MOHMQuit)**	• **MOHMQuit leadership components which focus on repeated audit and feedback plus action planning; developing and implementing a clinical pathway for SCS; and the development and maintenance of SCS 'champions' within each service**<br>• **The train the trainer model as an integral part of the intervention to support its ongoing implementation**<br>• **The Community of Practice**	**Site level**<br>**Individual clinician level**	**Proctor[1]**<br>**RE-AIM[2] (Maintenance)**<br>**Rogers[5]**	• **6 months following training - questionnaire with clinicians**<br>• **6 months following training - semi-structured interview with leaders**<br>• **Community of Practice peer support attendance data**	**<u>6 month questionnaire for clinicians</u>**<br>**** How useful were each of the MOHMQuit resources when working with women (scale of 1-3 Very useful** to **Not at all useful + Not Applicable as a response option)?***<br>**<u>6 month interview guide for leaders</u>**<br>**** How much has MOHMQuit become part of 'usual practice'?***<br>**** What do you think helped in delivering MOHMQuit? (contextual factors)***<br>**<u>Community of Practice meetings</u>**<br>*** Sites attending community of practice meetings**
Acceptability (how palatable is MOHMQuit to	• Comprehensive and systematic design of MOHMQuit using the	Site level<br>Individual level	Proctor[1]<br>Sekhon[8]	• Immediately following training - questionnaire with clinicians	<u>Immediately following training for clinicians</u>:<br>* *On a scale of 1 to 3 (very useful* to *not at all useful) what's your impression of how useful the MOHMQuit training is going*

Implementation outcome (abbreviated definition)	Strategies used to maximise implementation outcomes	Level of analysis	Frameworks used	Data collection instruments, timing, participants	Items from instruments
clinicians and leaders?)	Behaviour Change Wheel integrating input from clinicians and leaders[6 7]<br>• Feasibility and acceptability trial with subsequent minor amendments to the intervention[6]<br>• At the 10 week warm-up the long 'history' of the development of MOHMQuit is described to ensure leaders are reassured about its quality, relevance and acceptability			• 3 months following training - questionnaire for leaders<br>• 6 months following training - questionnaire with clinicians<br>• 6 months following training - semi-structured interview with leaders	*to be in helping you provide SCS by addressing gaps in your knowledge/skills/confidence?* (perceived effectiveness[8]);<br>* *On a scale of 1-3 (very much to not at all) how much do you think MOHMQuit will help you provide SCS* (perceived effectiveness[8]);<br>**Overall how do you feel about MOHMQuit (scale of 1-4)?* (affective attitude[8])<br><u>3 month questionnaire for leaders</u>:<br>* *Please give MOHMQuit a score of whether you think it has helped your service to routinely provide evidence-based SCS (scale of 1-10 Has not helped at all* to *Has been entirely helpful)* (perceived effectiveness[8]);<br><u>6 month questionnaire for clinicians</u>:<br>* *On a scale of 1-5 (Strongly agree* to *Strongly disagree) I am confident providing smoking cessation assistance to pregnant women* (self-efficacy[8]);<br>* *On a scale of 1-5 (Strongly agree* to *Strongly disagree) I am confident arranging follow up support for pregnant smokers* (self-efficacy[8]);<br>* *On a scale of 1-4* (*very much* to *not at all*) *to what extent did MOHMQuit help you to provide high quality smoking cessation support to women at every visit?* (perceived effectiveness[8])<br><u>6 month interview guide for leaders</u><br>* *How would you describe MOHMQuit (what it is and how it aims to improve practice) to a leader in a maternity service in a different hospital?* (intervention coherence[8])<br>* *Did MOHMQuit improve the SCS provided to pregnant women in your service?* (perceived effectiveness[8])?

Implementation outcome (abbreviated definition)	Strategies used to maximise implementation outcomes	Level of analysis	Frameworks used	Data collection instruments, timing, participants	Items from instruments
Appropriateness (perceived fit or relevance of MOHMQuit with the service)	• Comprehensive and systematic design of MOHMQuit using the Behaviour Change Wheel integrating input from clinicians and leaders[6 7]<br>• Feasibility and acceptability trial with subsequent minor amendments to the intervention[6]<br>• At the 10 week warm-up the long 'history' of the development of MOHMQuit is described to ensure leaders are reassured about its quality, relevance and acceptability	Site level	Proctor[1]<br>Rogers[5]	• 6 months following training - semi-structured interview with leaders	6 month interview guide for leaders<br>* *Were any adaptations made to MOHMQuit? (What/who/when/why/how?)*
Feasibility (actual fit – the extent to which MOHMQuit can be integrated into usual care in a service)	• Comprehensive and systematic design of MOHMQuit using the Behaviour Change Wheel integrating input from clinicians and leaders[6 7]	Site level	Proctor[1]<br>Rogers[5]	• 3 months following training - questionnaire for leaders<br>• 6 months following training - semi-structured interview with leaders	3 month questionnaire for leaders<br>* *In the last 3 months did you or any other staff in your service…* (followed by a number of MOHMQuit components)*?*<br>6 month interview guide for leaders<br>* *Have you, or any of your colleagues implemented any of the components of MOHMQuit* (followed by a number of MOHMQuit components)*?*

Implementation outcome (abbreviated definition)	Strategies used to maximise implementation outcomes	Level of analysis	Frameworks used	Data collection instruments, timing, participants	Items from instruments
	• Feasibility and acceptability trial with subsequent minor amendments to the intervention[6]<br>• At the 10 week warm-up the long 'history' of the development of MOHMQuit is described to ensure leaders are reassured about its quality, relevance and acceptability				** How much has MOHMQuit become part of 'usual practice'? (e.g. standard operating procedures, local policies, SCS as a standing item on meeting agendas, audit part of usual audit schedule etc.)*
HOW the implementation of the intervention changed behaviour – the 'mechanisms of impact'+ acceptability, appropriateness and feasibility above		Site level	Moore[4]	• 6 months following training - semi-structured interview with leaders	6 month interview guide for leaders<br>** Did MOHMQuit improve the SCS provided to pregnant women in your service? How did it do this?*<br>** How can the implementation of MOHMQuit be improved?*
**HOW context affected implementation**	• **Commitment of maternity service leaders in the**	**Site level**	**Fernandez[9]**	• **Key contextual information (Table 1) completed by research**	**See Table 1 above**<br>*** Birth numbers; smoking prevalence; Performance against the performance indicator of antenatal smoking; Safer Baby**

Implementation outcome (abbreviated definition)	Strategies used to maximise implementation outcomes	Level of analysis	Frameworks used	Data collection instruments, timing, participants	Items from instruments
	research as Partner Investigators on the grant and as members of the MOHMQuit research Steering Committee and various working groups<br>• Warm-up meetings<br>• Follow up meetings<br>• Community of Practice			team during the implementation<br>• 3 months following training - questionnaire for leaders<br><br>• 6 months following training - questionnaire with clinicians<br><br>• 6 months following training - semi-structured interview with leaders	**Bundle; RSVP policy; Other SCS initiatives; Accreditation; leadership structure; models of care on offer; other**<br><br>**3 month questionnaire for leaders**<br>**** Please indicate the extent to which you agree (from Not at all*** **to** ***Very great extent)…*** **all 12 items from the Implementation Leadership Scale[10] e.g.** ***I have developed a plan to facilitate the implementation of MOHMQuit***<br>**6 month questionnaire for clinicians**<br>**** How well do you feel your service leadership has supported the implementation of MOHMQuit*** **(scale of 1-5 from** ***Strongly Disagree*** **to** ***Strongly*** **agree) the 4 items of the Implementation Climate measure[9]** ***e.g. Our service leadership makes sure that we have the time and space necessary to discuss changes to improve care***<br>**** …the general feeling for implementation of MOHMQuit in your service*** **(scale of 1-5 from** ***Strongly Disagree*** **to** ***Strongly*** **agree) the 4 items from the Leadership Engagement measure[9]** ***e.g. Our service staff get the support they need to implement MOHMQuit***<br>**6 month interview guide for leaders**<br>**** Has anything changed in terms of your or others' leadership within the service/s around SCS due to MOHMQuit? (Why? How?)***<br>**** What do you think helped in delivering MOHMQuit?***<br>**** What made delivering MOHMQuit more of a challenge?***

BOLD TYPEFACE INDICATES OUTCOMES THAT WILL BE THE FOCUS OF THE PROCESS EVALUATION

Implementation cost is not included in Table 2 as a detailed economic evaluation of MOHMQuit is taking place and is the subject of a separate paper.[11] Data to contribute to the economic evaluation will be collected as part of the semi-structured interview with leaders.

References

1. Proctor E, Silmere H, Raghavan R, et al. Outcomes for implementation research: conceptual distinctions, measurement challenges, and research agenda. *Administration and policy in mental health and mental health services research* 2011;38(2):65-76.
2. Glasgow RE, Vogt TM, Boles SM. Evaluating the public health impact of health promotion interventions: the RE-AIM framework. *Am J Public Health* 1999;89(9):1322-27.
3. Barnes LAJ, Longman J, Adams C, et al. The MOHMQuit (Midwives and Obstetricians Helping Mothers to Quit Smoking) Trial: protocol for a stepped-wedge implementation trial to improve best practice smoking cessation support in public antenatal care services. *Implement Sci* 2022;17(1):79. doi: 10.1186/s13012-022-01250-3
4. Moore GF, Audrey S, Barker M, et al. Process evaluation of complex interventions: Medical Research Council guidance. *bmj* 2015;350
5. Rogers EM. Diffusion of Innovations. 4th ed. New York: The Free Press 1995.
6. Passey ME, Adams C, Paul C, et al. Improving implementation of smoking cessation guidelines in pregnancy care: Development of an intervention to address system, maternity service leader and clinician factors. *Implementation Science Communications* 2021;2:128. doi: https://doi.org/10.1186/s43058-021-00235-5
7. Longman J, Adams C, Paul C, et al. Improving clinicians' implementation of guidelines to help women stop smoking in pregnancy: developing evidence-based print and video materials. *Int J Environ Res Public Health* 2021;18(19):10522. doi: https://doi.org/10.3390/ijerph181910522
8. Sekhon M, Cartwright M, Francis JJ. Acceptability of healthcare interventions: an overview of reviews and development of a theoretical framework. *BMC Health Serv Res* 2017;17(1):88.
9. Fernandez ME, Walker TJ, Weiner BJ, et al. Developing measures to assess constructs from the Inner Setting domain of the Consolidated Framework for Implementation Research. *Implement Sci* 2018;13(1):52. doi: 10.1186/s13012-018-0736-7
10. Aarons GA, Ehrhart MG, Farahnak LR. The Implementation Leadership Scale (ILS): Development of a Brief Measure of Unit Level Implementation Leadership. *Implement Sci* 2014;9:45. doi: 10.1186/1748-5908-9-45
11. Pearce A, Scarfe J, Jones M, et al. Study protocol of an economic evaluation embedded in the Midwives and Obstetricians Helping Mothers to Quit Smoking (MOHMQuit) trial. *BMC Health Serv Res* 2023;23(1):1-9.
