# Supplementary material for: Protocol for the process evaluation of an intervention to improve antenatal smoking cessation support (MOHMQuit) in maternity services in New South Wales, Australia
Source: BMJ Open. 2024 Mar 19;14(3):e081208. doi: 10.1136/bmjopen-2023-081208 (PMC10952879; doi:10.1136/bmjopen-2023-081208)
Supplement: Author's manuscript [file bmjopen-2023-081208.draft_revisions.pdf]

BMJ Open is committed to open peer review. As part of this commitment we make the peer review history of every article we publish publicly available.

When an article is published we post the peer reviewers' comments and the authors' responses online. We also post the versions of the paper that were used during peer review. These are the versions that the peer review comments apply to.

The versions of the paper that follow are the versions that were submitted during the peer review process. They are not the versions of record or the final published versions. They should not be cited or distributed as the published version of this manuscript.

BMJ Open is an open access journal and the full, final, typeset and author-corrected version of record of the manuscript is available on our site with no access controls, subscription charges or pay-per-view fees (http://bmjopen.bmj.com).

If you have any questions on BMJ Open's open peer review process please email
info.bmjopen@bmj.com

BMJ Open

Protocol for the process evaluation of an intervention to improve antenatal smoking cessation support (MOHMQuit) in maternity services in New South Wales, Australia

Journal:	*BMJ Open*
Manuscript ID	bmjopen-2023-081208
Article Type:	Protocol
Date Submitted by the Author:	25-Oct-2023
Complete List of Authors:	Longman, Jo; The University of Sydney, University Centre for Rural Health<br>Paul, Christine; University of Newcastle, School of Medicine and Public Health<br>Cashmore, Aaron; NSW Health, Centre for Epidemiology and Evidence; The University of Sydney, School of Public Health, Faculty of Medicine and Health<br>Twyman, Laura; Cancer Council NSW; The University of Newcastle, School of Medicine and Public Health<br>Barnes, Larisa; The University of Sydney, University Centre for Rural Health<br>Adams, Catherine; NSW Health, Clinical Excellence Commission<br>Bonevski, Billie; Flinders University, College of Medicine & Public Health<br>Milat, Andrew; The University of Sydney, School of Public Health<br>Passey, Megan; The University of Sydney, Daffodil Centre and the University Centre for Rural Health, Faculty of Medicine and Health
Keywords:	Implementation Science, Behavior, Primary Health Care

SCHOLARONE™
Manuscripts

Protocol for the process evaluation of an intervention to improve antenatal smoking cessation support (MOHMQuit) in maternity services in New South Wales, Australia

Jo Longman[1], Chris Paul[2], Aaron Cashmore[3,4], Laura Twyman[5], Larisa AJ Barnes[1,6], Cathy Adams[7], Billie Bonevski[8], Andrew Milat[3,4] and Megan E Passey[1,6]

[1] The University of Sydney, The University Centre for Rural Health, 61 Uralba St., Lismore, NSW 2480, Australia.
[2] University of Newcastle, School of Medicine and Public Health, University Drive, Callaghan, NSW 2308, Australia.
[3] NSW Ministry of Health, Centre for Epidemiology and Evidence, 1 Reserve Road, St Leonards, NSW 2065, Australia.
[4] The University of Sydney, Sydney School of Public Health, Faculty of Medicine and Health, Camperdown, NSW 2006, Australia.
[5] Tobacco Control Unit, Cancer Prevention and Advocacy Division, Cancer Council NSW, and Conjoint Fellow, School of Medicine and Public Health, University of Newcastle, 153 Dowling St., Woolloomooloo, NSW 2011, Australia
[6] The University of Sydney, Daffodil Centre, PO Box 572 Kings Cross NSW 1340, Australia.
[7] Maternity and Neonatal Safety Program; Clinical Excellence Commission, 1 Reserve Road, St Leonards, NSW 2065
[8] Flinders University, College of Medicine & Public Health, Flinders Health and Medical Research Institute, Sturt Road, Bedford Park, SA 5042, Australia

Corresponding author:

Larisa A.J. Barnes
larisa.barnes@sydney.edu.au
Daffodil Centre and the University Centre for Rural Health
Faculty of Medicine and Health
The University of Sydney

For peer review only

For peer review only - http://bmjopen.bmj.com/site/about/guidelines.xhtml

ABSTRACT

Introduction

Smoking cessation in pregnancy remains a public health priority. Our team used the Behaviour Change Wheel to develop the MOHMQuit intervention (Midwives and Obstetricians Helping Mothers to Quit smoking) with health system, leader and clinician components. MOHMQuit addresses a critical evidence to practice gap in the provision of smoking cessation support in antenatal care. It involves nine maternity services in NSW in a cluster randomised stepped-wedge controlled trial of effectiveness. This paper describes the design and rationale for the process evaluation of MOHMQuit. The process evaluation aims to assess to what extent and how MOHMQuit is being implemented (acceptability; adoption/uptake; appropriateness; feasibility; fidelity; penetration and sustainability), and the context in which it is implemented, in order to support further refinement of MOHMQuit throughout the trial, and aid understanding and interpretation of the results of the trial.

Methods and analysis

The process evaluation is an integral part of the stepped-wedge trial. Its design is underpinned by implementation science frameworks and adopts a mixed methods approach. Quantitative evidence from participating leaders and clinicians in our study will be used to produce individual and site-level descriptive statistics. Qualitative evidence of leaders' perceptions about the implementation will be collected using semi-structured interviews and will be analysed descriptively within-site and thematically across the dataset. The process evaluation will also use publicly-available data and observations from the research team implementing MOHMQuit e.g. training logs. These data will be synthesised to provide site-level as well as individual-level implementation outcomes.

Ethics and dissemination

The study received ethical approval from the Population Health Services Research Ethics Committee for NSW, Australia (Reference 2021/ETH00887). Results will be communicated to the process evaluation participants via the study's Steering Committee and will also be published in peer-reviewed journals and presented at conferences.

Trial registration

Australian New Zealand Trials Registry ACTRN12622000167763
https://www.australianclinicaltrials.gov.au/anzctr/trial/ACTRN12622000167763

Keywords

Implementation science, Behavior, Primary health care, Smoking cessation support, Pregnancy, Antenatal care, Systems change intervention, Stepped-wedge cluster-randomised controlled trial; evaluation studies as topic; process evaluation

For peer review only - http://bmjopen.bmj.com/site/about/guidelines.xhtml

Strengths and limitations of this study

► The process evaluation has been designed using implementation science frameworks

► The study uses multiple data sources. Qualitative and quantitative data will be collected independently from leaders and clinicians in each MOHMQuit site as well as contextual and publicly-available data, and observational data from the research team implementing MOHMQuit

► MOHMQuit is a complex intervention with many moving parts which interact with one another and the stakeholders involved. No process evaluation is able to collect data to understand all aspects of these interactions, particularly not in a 'real world' trial such as this one.

For peer review only

For peer review only - http://bmjopen.bmj.com/site/about/guidelines.xhtml

INTRODUCTION

In 2020, 9.2% of mothers in Australia smoked tobacco at some point during their pregnancy.[1] Smoking in pregnancy is associated with a multitude of adverse outcomes for both mother and baby including pre-term birth and small for gestational age babies.[2-5] Smoking is the most important modifiable risk for adverse outcomes and therefore supporting pregnant women to stop smoking remains a major public health concern and a priority for the New South Wales (NSW) Ministry of Health.[6-8] Clinical guidelines for NSW have existed for almost 20 years and recommend clinicians routinely provide evidence-based smoking cessation support (SCS) at all antenatal care visits for women who smoke or who have stopped smoking in this pregnancy.[9] Implementation of the Guidelines shows room for improvement.[10-13] This fact, along with wider evidence that women want to stop smoking in pregnancy but some lack confidence to do so, [14] would value support from their clinicians[15] and a systematic review demonstrating that psychosocial interventions helps women to stop smoking, [16] led us to develop a theoretically underpinned intervention, MOHMQuit (Midwives and Obstetricians Helping Mothers to Quit smoking).

The MOHMQuit intervention

The MOHMQuit intervention has multiple components targeting different parts of a complex health system.[17] It is based on the '5As' of SCS (Ask, Advise, Assess, Assist and Arrange Follow-up) which has shown evidence of effectiveness for SCS.[18] MOHMQuit was specifically designed to improve antenatal care clinicians' implementation of the NSW Guidelines, and was developed using the Behaviour Change Wheel.[19] It is an intervention built on local and international evidence identifying barriers and enablers for health systems, leaders and clinicians providing SCS.[20] It focuses on changing behaviours by targeting systems, leaders and clinicians (see Figure 1 for a glossary of terms). MOHMQuit is being implemented in a stepped-wedge trial across five Local Health Districts in NSW with diverse characteristics including organisational structure and staffing profiles.

Figure 1: Glossary of terms

**Antenatal Care clinicians**	• Antenatal care midwives<br>• Aboriginal Health Workers (AHWs) - primary healthcare workers who ensure culturally safe maternity care in supporting Aboriginal and/or Torres Strait Islander women or women having an Aboriginal baby<br>• Obstetricians (staff specialists; Visiting Medical Officers with specialist obstetric training, Career Medical Officers) and obstetric registrars
**Leaders**	Maternity service leaders (those who support or supervise health professionals providing antenatal care), including:<br>• Clinical Midwifery Consultants<br>• Maternity Unit Managers<br>• Clinical Midwifery Educators<br>• Clinical Midwifery Specialists<br>• Antenatal clinic coordinators<br>• Obstetric leads

The development of the MOHMQuit intervention and its support materials have been described in detail previously.[20 21] In brief, there are four main components (also referred to in the implementation science literature as 'implementation strategies'): (1) separate training events for: maternity service leaders (half day), midwives/AHWs (full day) and obstetricians (two hours).

For peer review only - http://bmjopen.bmj.com/site/about/guidelines.xhtml

Midwifery educators also take part in the leaders' and midwives' training events as a 'train the trainer' model (which includes a comprehensive MOHMQuit training manual) is central to the sustainability of the intervention; (2) a number of MOHMQuit leadership processes and systems tools e.g. a report template for the electronic medical record system facilitating leaders' scrutiny of their services' SCS performance; a service audit tool for leaders; (3) MOHMQuit written resources such as a booklet on 'Stopping smoking for you and your baby' for clinicians to use with women; and (4) a series of 11 short video clips for training and skills development to be used in a wide variety of settings e.g. at handover meetings.

Two months prior to the implementation starting in the first site, a day-long face to face gathering was held bringing together key decision makers and clinicians from across the sites to ensure a shared awareness and understanding of MOHMQuit (including its history and rationale), promote enthusiasm, motivation and engagement and establish shared understanding about roles and responsibilities.

At each site, ten weeks prior to the intervention the research team and the maternity service leaders will participate in a 'warm-up' meeting. Whilst each site has a strong existing connection with MOHMQuit via the face to face day, and through the inclusion of partner investigators at each site, the warm-up meeting includes: acknowledging and thanking those involved (which extend beyond the site partner investigators and include the antenatal clinic coordinator, the clinical midwifery educator and other leaders), generating enthusiasm, building momentum in the lead up to the implementation of MOHMQuit, and working through the logistics of implementation at each site. Two weeks prior to the intervention a second meeting will be held which has a 'trouble-shooting' agenda and will also include detail of the research elements of MOHMQuit for example how and when outcome and process evaluation data from the site will be collected. Additional meetings are planned for two and four weeks post-intervention, to maintain momentum and explore any unresolved issues in the ongoing implementation of MOHMQuit. A MOHMQuit Community of Practice will be established which each site can join following implementation. The Community of Practice will offer a regular forum for sharing and supporting other clinicians and leaders in continuing to implement MOHMQuit and is one of several sustainability features of MOHMQuit. Finally, three and a half months after implementation, each site will receive feedback from brief interviews with women about the smoking cessation support they received during their antenatal care. They will continue to receive these reports quarterly until the end of the trial.

MOHMQuit is currently being trialled in a multi-site cluster randomised stepped-wedge effectiveness trial in nine sites in publicly-funded maternity services in NSW, Australia.[22] Implementation is planned to take place over a 13 month timeframe. Unlike many earlier interventions aimed at improving SCS, MOHMQuit is built on implementation science frameworks and is specific to the public maternity service setting. The trial will assess the intervention outcomes. The primary intervention outcome is smoking cessation, and secondary intervention outcomes include changes to clinicians' knowledge, skills, confidence and behaviour in providing SCS and test the 'mechanisms of action'[23-25] by which each of the components/strategies effect intervention outcomes and moderators of their impact in this framework-driven approach.[22] Cost-effectiveness will be assessed in an economic evaluation.[26] The trial will also assess implementation outcomes (assessing how MOHMQuit was implemented) in a detailed process evaluation. The process evaluation will complement the assessment of the MOHMQuit intervention outcomes. Conducting process evaluation alongside effectiveness trials in this way is recommended.[27 28]

For peer review only - http://bmjopen.bmj.com/site/about/guidelines.xhtml

Aims of the MOHMQuit process evaluation

Process evaluations explore how an intervention is implemented. They assess three aspects: (a) how and to what extent the intervention was implemented; (b) the 'mechanisms of impact' i.e. how the intervention components and participants' interactions with these components effected changes in behaviour; and (c) the context in which the intervention was implemented.[29] We anticipate that the process evaluation will contribute formatively by providing feedback that may further refine the intervention. This is particularly useful in a stepped-wedge trial design where each site joins the trial sequentially, and acceptable as long as the changes made to components retain the integrity of the function they were meant to perform in the original intervention design.[30 31] The summative use of process evaluation is in providing insight into the mechanisms through which the intervention outcomes (the primary intervention outcome being pregnant women stopping smoking), were achieved or not, and therefore it will contribute to understanding and interpreting the results of the effectiveness trial.[32] Without this insight effective (and ineffective) aspects of the intervention may not be understood and this has implications for the scale-up of an intervention such as MOHMQuit. In this way, the process evaluation will maximise the knowledge gained throughout the trial and describe the most effective delivery processes for the MOHMQuit intervention. The aim of this protocol paper is to describe the process evaluation planned as an integral part of the MOHMQuit trial.

METHODS AND ANALYSIS

Overall design and objectives of the process evaluation

The design for the process evaluation began with the implementation outcomes defined by Enola Proctor and team in order to facilitate an understanding of the various dimensions of the implementation: acceptability; adoption/uptake; appropriateness; feasibility; fidelity; penetration and sustainability (and sustainment).[33] Implementation outcomes are "…the effects of deliberate and purposive actions to implement new…practices".[33] The Proctor implementation outcomes generally map on to other well-used frameworks such as the RE-AIM framework [28] but 'Reach' from the RE-AIM framework was specifically added into the design to capture the number of clinicians and leaders invited to and taking part in the trial. Two other frameworks informed the implementation outcomes of interest (Sekhon[34] for acceptability Rogers[35] for sustainability, appropriateness and feasibility) and Moore[32] and Fernandez'[36] work guided exploration of mechanisms of impact and how context affected implementation. The context in which the intervention was implemented will also be assessed. Context is variously defined[37] but here contextual features are conceived of broadly as those influencing the delivery of the intervention and include the engagement of leaders and the organisational setting and culture of the service in which the intervention is implemented.[38]

Important features of the process of implementing MOHMQuit were discussed and agreed with a process evaluation working group of the project's Steering Committee (a key governance committee of the project and constituted of research academics, policy makers, managers and leaders[20]). From there, instruments were developed which encompassed both individual and service level observations, and decisions were made about the focus, given that "Process evaluations cannot expect to provide answers to all of the uncertainties of a complex intervention. It is generally better to answer the most important questions well than to try to answer too many questions and do so unsatisfactorily." [32]

For peer review only - http://bmjopen.bmj.com/site/about/guidelines.xhtml

With that in mind, a focus on fidelity; adoption/uptake; penetration; reach, sustainability and context was agreed. In part these foci were based on learning from the feasibility and acceptability trial of MOHMQuit.[20] In addition, the short duration of the trial (the time from implementation at the first site to the end of data collection, excluding the wash out period, is 24 months and from the final site, only 8 months) would make sustainment challenging to measure. Sustainability is, however, included in the evaluation. Sustainment is "the continued use of a practice that is the target of the implementation, whereas sustainability addresses whether the factors are in place to promote the ongoing use." [39]

The process evaluation has three interrelated objectives; to (at the individual and site level) assess:

1. To what extent MOHMQuit was implemented - measured quantitatively focusing on the implementation outcomes of adoption, fidelity, penetration, reach and sustainability, and will also involve qualitative measures (interviews with leaders)
2. How changes in behaviour were effected (the mechanisms of impact) – measured quantitatively focusing on the implementation outcomes of acceptability, appropriateness and feasibility, and a more nuanced understanding of this from leaders' perspectives in qualitative interviews
3. The impact of context (moderators) on the implementation of MOHMQuit. A moderator is a factor that will strengthen or lessen the influence of a strategy to implement MOHMQuit.[24] We anticipate a number of moderators will be an important part of the context for MOHMQuit implementation (as well as intervention outcomes), affecting the relationship between the implementation outcomes (e.g. reach), and the implementation of MOHMQuit. The moderators measured include:

    a. Leadership
        i. Leaders self-assessment of their leadership for implementation at 3 months using the Implementation Leadership Scale[40]
        ii. Clinicians questionnaires at 6 months which include the Leadership Engagement Scale[36]
    b. Implementation climate
        i. Clinician questionnaires at 6 months which include the Implementation Climate Scale[36]
    c. Service Size
    d. Smoking prevalence amongst pregnant women birthing at that site
    e. Other demands on leaders/service (e.g. new SCS policies and training or accreditation)

In summary, we speculate that the impact of the context on the implementation outcomes could be as follows:

- Leadership and implementation climate - impacting on all outcomes
- Service size, smoking prevalence and models of care - impacting on adoption, appropriateness, feasibility, penetration and sustainability
- Other demands on leaders - impacting on implementation in terms of adoption, fidelity, penetration and sustainability

see Figure 2 below

For peer review only - http://bmjopen.bmj.com/site/about/guidelines.xhtml

Figure 2: impact of the context on the implementation outcomes

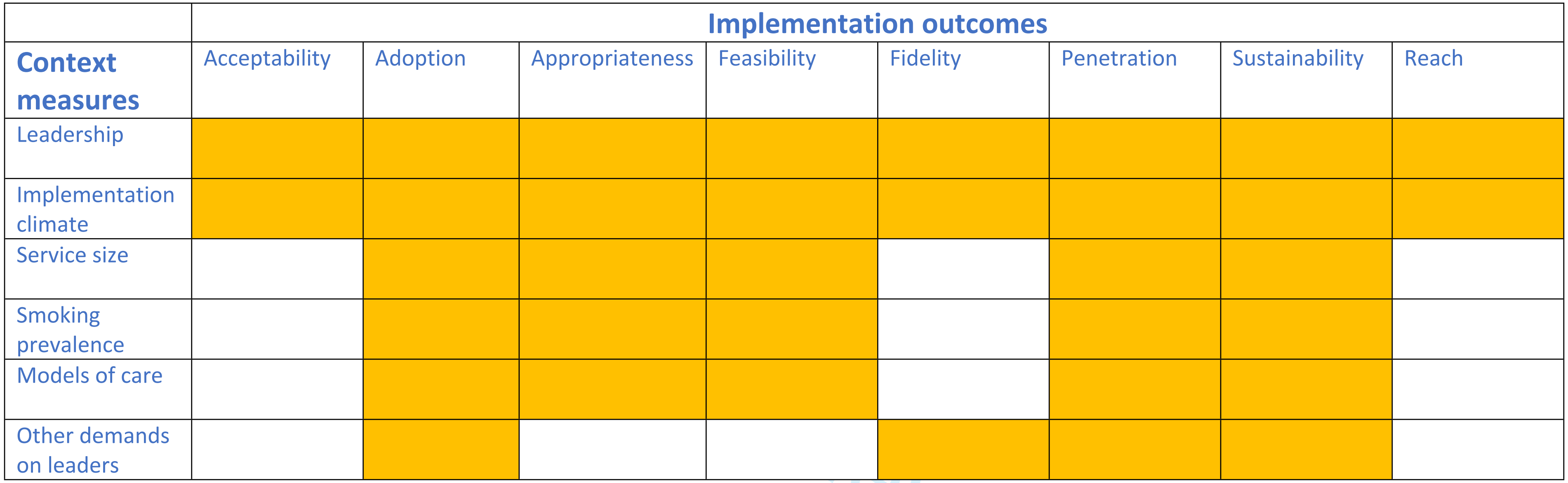

For peer review only - http://bmjopen.bmj.com/site/about/guidelines.xhtml

Recruitment and consent

The Local Health Districts (LHDs – which manage public hospitals and provide healthcare services in a defined geographic area) in NSW with relatively high rates of smoking in pregnancy were approached to participate in the MOHMQuit trial. There are 15 LHDs in total, seven with high smoking rates in pregnancy were invited and five agreed to participate in the trial. Between them they selected nine maternity services (sites) to take part. The senior midwives and lead obstetricians from these five LHDs were partner investigators in a Partnership Grant application subsequently awarded by Australia's National Health and Medical Research Council and so their involvement with the project substantially precedes the implementation trial of MOHMQuit.

Individual service leaders and clinicians in each of the nine sites will be provided with a Participant Information Sheet and those who agree to participate in the research will be asked to sign a written consent form indicating their consent to take part in data collection. This consent applies to data collection to measure the implementation outcomes and context as well as the intervention outcomes.

Process evaluation data collection

The process evaluation will adopt a mixed methods approach, collecting quantitative evidence from questionnaires and qualitative evidence of leaders' perceptions of how MOHMQuit may have changed behaviour (where it was perceived to have done so) from semi-structured interviews. Data will be collected by the research team independently from each of the nine MOHMQuit sites. Study-specific questionnaires will be used to collect implementation outcome data from leaders and clinicians at each site at various time points: immediately following training, three months after the training and six months after the training as outlined below. To minimise participant burden, the questionnaires will also collect the data required to measure the intervention outcomes.

Qualitative data will be collected using semi-structured interviews with leaders six months after the training at each site. The interviews have three key purposes. Firstly, interviews will collect data on the components of MOHMQuit which have been implemented in the six months following the MOHMQuit training (uptake) e.g. use of the report template for the electronic medical record system facilitating leaders' scrutiny of their services' SCS performance for feedback and continuous improvement, or MOHMQuit training delivered by the service themselves (using the train the trainer manual). Secondly, they will collect data to support the calculation of an implementation cost as part of the detailed economic evaluation of MOHMQuit, the subject of a separate paper, [26] by recording how much time leaders' assess they spend implementing those components of MOHMQuit. Finally, they will collect data which will enhance the contextual information collected by the research team by eliciting leaders' perspectives of the enablers and barriers of the implementation of MOHMQuit and what might be improved with regard to it. Interviews will be conducted using the Teams platform, recorded and transcribed. They will be guided by an interview schedule driven by the implementation outcomes and the contextual factors that supported or hindered implementation and any adaptations made to the intervention. The semi-structured nature of the interviews will allow for flexibility in questioning and expansion on responses.

Data collection from leaders and antenatal care clinicians will be as follows:

Leaders

- An online questionnaire to <u>all</u> leaders **three months after** the training at each site regardless of whether they attended MOHMQuit training.

For peer review only - http://bmjopen.bmj.com/site/about/guidelines.xhtml

- A semi-structured one to one telephone interview **six months after** the training with the midwifery partner investigator and one to two other leaders at each site.

Antenatal care clinicians

- A paper questionnaire **immediately following the training** at each site to participants who attended training.
- An online questionnaire to all antenatal care clinicians and AHWs **six months after** the training at each site regardless of whether they attended MOHMQuit training.

In addition, attendance and fidelity information (which aspects of the training were delivered) will be kept by the research team during each training event and the attendance and engagement at various meetings that are components of MOHMQuit. The additional data collection includes:

- Training logs – to calculate proportion attended at each training event (attendance/invited)
- A 'fidelity checklist' of which elements of the training were covered during each training event
- Attendance and notes from 10 week warm-up meetings
- Attendance and notes from 2 week warm-up meetings
- Attendance and notes from 2 week follow-up meetings
- Attendance and notes from 4 week follow-up meetings
- Attendance and notes from monthly Community of Practice meetings

For each site a 'context table' will be completed by the research team using publicly available sources and with input from partner investigators at each site (Table 1).

Table 1: Key contextual information collected for each site

**Number of births at site 2020**
**Smoking prevalence 2020**
**Performance against the NSW Ministry of Health's performance indicator of antenatal smoking**
**Safer Baby Bundle at site?* (Yes/No)**
**Preparation and training for new NSW Maternity Care Policy (RSVP)# overlaps with MOHMQuit timing? (Yes/No)**
**Other SCS initiatives running at the site? (Yes/No)**
**Accreditation for Quality Improvement going on concurrent with MOHMQuit? (Yes/No)**
**Leadership structure at the site**
**Models of care offered and proportion of women at booking and at birth for each model**
**Other e.g. external events like disasters, vacant posts**

* Safer Baby Bundle is a multi-component intervention in maternity service which aims to reduce the number of preventable stillbirths
# The RSVP policy is a policy directive establishing minimum requirements for health services to provide evidence-based smoking cessation support to women before during and after pregnancy. The RSVP Policy was released 14 October 2022.

We anticipate that the data collection itself may have the beneficial sustainability effect of reminding leaders and clinicians about MOHMQuit and possibly prompting renewed attention and/or commitment to it.

Table 2 provides detail of working definitions and how each of the implementation outcomes and contextual features will be measured at which timepoints, using which instruments with whom, and

For peer review only - http://bmjopen.bmj.com/site/about/guidelines.xhtml

which strategies (components of the MOHMQuit intervention) are aimed to maximise the implementation outcomes.

For peer review only

For peer review only - http://bmjopen.bmj.com/site/about/guidelines.xhtml

Table 2: Implementation outcomes, definitions, strategies for maximising implementation outcomes, frameworks used and measurement items

Implementation outcome (abbreviated definition)	Strategies used to maximise implementation outcomes	Level of analysis	Frameworks used	Data collection instruments, timing, participants	Items from instruments
**Adoption/Uptake (intention or action to try to employ MOHMQuit)**	• Warm-up meetings<br>• Follow up meetings<br>• Community of Practice	Site level<br>Individual clinician level	Proctor[33]<br>RE-AIM[28] (Adoption)	• Warm-up and follow up meetings<br>• 3 months following training - questionnaire for leaders<br>• 6 months following training - questionnaire with clinicians<br>• 6 months following training - semi-structured interview with leaders<br>• Community of Practice peer support meetings attendance data	<u>Meetings</u><br>* Whether the 10 and 2-week warm-up meetings took place/were attended;<br>* Whether the 2 and 4 week post training meetings took place/were attended<br><u>3 month questionnaire for leaders</u><br>* *In the last 3 months did you or any other staff in your service…* (followed by a number of MOHMQuit components)*?*<br><u>6 month questionnaire for clinicians</u><br>* *How useful were each of the MOHMQuit resources when working with women (scale of 1-3 Very useful to Not at all useful + Not Applicable as a response option)?*<br><u>6 month interview guide for leaders</u><br>* *Have you, or any of your colleagues implemented any of the components of MOHMQuit* (followed by a number of MOHMQuit components)*?*<br>* *What do you think helped in delivering MOHMQuit?*<br><u>Community of Practice meetings</u><br>* Sites attending community of practice meetings
**Fidelity (delivered as intended in the Protocol[22], adherence)**	• Warm-up and follow-up meetings<br>• Consistency in the team delivering MOHMQuit training at each site in the first instance	Site level	Proctor[33]<br>RE-AIM[28] (Implementation)	• Warm-up and follow up meetings<br>• Training logs of expected and actual	<u>Meetings</u><br>* Whether the 10 and 2-week warm-up meetings took place/were attended;<br>* Whether the 2 and 4 week post training meetings took place/were attended<br><u>Training logs</u>

For peer review only - http://bmjopen.bmj.com/site/about/guidelines.xhtml

Implementation outcome (abbreviated definition)	Strategies used to maximise implementation outcomes	Level of analysis	Frameworks used	Data collection instruments, timing, participants	Items from instruments
	• clear plans and materials for content of training			attendance at training of leaders and clinicians recorded at the time of training<br>• Fidelity record (a checklist of which aspects of the planned training were actually delivered – completed by researchers observing the training, plus any additional observational data about engagement of participants)<br>• 6 months following training - semi-structured interview with leaders	* Proportion of eligible leaders and clinicians who were invited and who actually attended training<br><br>Fidelity record<br>* the extent to which training was delivered as anticipated<br><br>6 month interview guide for leaders<br>* *Were any adaptations made to MOHMQuit? (What/who/when/why/how?)*
Penetration (degree of integration of MOHMQuit practices within the service)	• Involving leaders in the training for clinicians for a whole-of-service approach<br>• MOHMQuit leadership components which focus on repeated audit and feedback plus action planning;	Site level	Proctor[33]<br>RE-AIM[28] (Adoption)	• 3 months following training - questionnaire for leaders<br>• 6 months following training - semi-structured interview with leaders	3 month questionnaire for leaders<br>* *In the last 3 months did you or any other staff in your service…* (followed by a number of MOHMQuit components)*?*<br>6 month interview guide for leaders<br>* *Have you, or any of your colleagues implemented any of the components of MOHMQuit* (followed by a number of MOHMQuit components)*?*<br>* *How much has MOHMQuit become part of 'usual practice'? (e.g. standard operating procedures, local policies, SCS as a*

For peer review only - http://bmjopen.bmj.com/site/about/guidelines.xhtml

Implementation outcome (abbreviated definition)	Strategies used to maximise implementation outcomes	Level of analysis	Frameworks used	Data collection instruments, timing, participants	Items from instruments
	developing and implementing a clinical pathway for SCS; and the development and maintenance of SCS 'champions' within each service<br>• The train the trainer model as an integral part of the intervention to support its ongoing implementation				***standing item on meeting agendas, audit part of usual audit schedule etc.)***
**Reach (did MOHMQuit include all clinicians and leaders that it aimed to?)**	• **10-week warm-up meetings to allow time for planning and rostering**<br>• **The train the trainer model as an integral part of the intervention to support participation of all relevant existing and new staff**	**Site level**	**RE-AIM[32]**	• **Training logs of expected and actual attendance at training of leaders and clinicians recorded at the time of training**<br>• **3 months following training - questionnaire for leaders**<br>• **6 months following training - semi-**	**Training logs**<br>*** Proportion of eligible leaders and clinicians who were invited and proportion who actually attended training (compare the seniority, and role e.g. midwife, obstetrician of those who participated to those who did not)**<br><br>**3 month questionnaire for leaders**<br>**** In the last 3 months did you or any other staff in your service design and run any staff training on SCS?*** **(the train the trainer model);**<br>**** Please tell us more about this training*** **(space to write a qualitative response)**<br>**6 month interview guide for leaders**

For peer review only - http://bmjopen.bmj.com/site/about/guidelines.xhtml

Implementation outcome (abbreviated definition)	Strategies used to maximise implementation outcomes	Level of analysis	Frameworks used	Data collection instruments, timing, participants	Items from instruments
				structured interview with leaders	* *Have you, or any of your colleagues implemented any of the components of MOHMQuit* (followed by a number of MOHMQuit components including *Designing and running any staff training*)?
**Sustainability (whether factors are in place to promote the ongoing use of MOHMQuit)**	• **MOHMQuit leadership components which focus on repeated audit and feedback plus action planning; developing and implementing a clinical pathway for SCS; and the development and maintenance of SCS 'champions' within each service**<br>• **The train the trainer model as an integral part of the intervention to support its ongoing implementation**<br>• **The Community of Practice**	**Site level Individual clinician level**	**Proctor[33] RE-AIM[28] (Maintenance) Rogers[35]**	• **6 months following training - questionnaire with clinicians**<br>• **6 months following training - semi-structured interview with leaders**<br>• **Community of Practice peer support attendance data**	**6 month questionnaire for clinicians**<br>* ***How useful were each of the MOHMQuit resources when working with women (scale of 1-3 Very useful to Not at all useful + Not Applicable as a response option)?***<br>**6 month interview guide for leaders**<br>* ***How much has MOHMQuit become part of 'usual practice'?***<br>* ***What do you think helped in delivering MOHMQuit? (contextual factors)***<br>**Community of Practice meetings**<br>*** Sites attending community of practice meetings**
Acceptability (how palatable is MOHMQuit to	• Comprehensive and systematic design of MOHMQuit using the	Site level Individual level	Proctor[33] Sekhon[34]	• Immediately following training - questionnaire with clinicians	Immediately following training for clinicians:<br>* *On a scale of 1 to 3 (very useful to not at all useful) what's your impression of how useful the MOHMQuit training is going*

For peer review only - http://bmjopen.bmj.com/site/about/guidelines.xhtml

Implementation outcome (abbreviated definition)	Strategies used to maximise implementation outcomes	Level of analysis	Frameworks used	Data collection instruments, timing, participants	Items from instruments
clinicians and leaders?)	Behaviour Change Wheel integrating input from clinicians and leaders[20 21]<br>• Feasibility and acceptability trial with subsequent minor amendments to the intervention[20]<br>• At the 10 week warm-up the long 'history' of the development of MOHMQuit is described to ensure leaders are reassured about its quality, relevance and acceptability			• 3 months following training - questionnaire for leaders<br>• 6 months following training - questionnaire with clinicians<br>• 6 months following training - semi-structured interview with leaders	*to be in helping you provide SCS by addressing gaps in your knowledge/skills/confidence?* (perceived effectiveness[34]);<br>* *On a scale of 1-3 (very much to not at all) how much do you think MOHMQuit will help you provide SCS* (perceived effectiveness[34]);<br>**Overall how do you feel about MOHMQuit (scale of 1-4)?* (affective attitude[34])<br>3 month questionnaire for leaders:<br>* *Please give MOHMQuit a score of whether you think it has helped your service to routinely provide evidence-based SCS (scale of 1-10 Has not helped at all to Has been entirely helpful)* (perceived effectiveness[34]);<br>6 month questionnaire for clinicians:<br>* *On a scale of 1-5 (Strongly agree to Strongly disagree) I am confident providing smoking cessation assistance to pregnant women* (self-efficacy[34]);<br>* *On a scale of 1-5 (Strongly agree to Strongly disagree) I am confident arranging follow up support for pregnant smokers* (self-efficacy[34]);<br>* *On a scale of 1-4* (very much to not at all) *to what extent did MOHMQuit help you to provide high quality smoking cessation support to women at every visit?* (perceived effectiveness[34])<br>6 month interview guide for leaders<br>* *How would you describe MOHMQuit (what it is and how it aims to improve practice) to a leader in a maternity service in a different hospital?* (intervention coherence[34])<br>* *Did MOHMQuit improve the SCS provided to pregnant women in your service?* (perceived effectiveness[34])?

For peer review only - http://bmjopen.bmj.com/site/about/guidelines.xhtml

Implementation outcome (abbreviated definition)	Strategies used to maximise implementation outcomes	Level of analysis	Frameworks used	Data collection instruments, timing, participants	Items from instruments
Appropriateness (perceived fit or relevance of MOHMQuit with the service)	• Comprehensive and systematic design of MOHMQuit using the Behaviour Change Wheel integrating input from clinicians and leaders[20,21]<br>• Feasibility and acceptability trial with subsequent minor amendments to the intervention[20]<br>• At the 10 week warm-up the long 'history' of the development of MOHMQuit is described to ensure leaders are reassured about its quality, relevance and acceptability	Site level	Proctor[33]<br>Rogers[35]	• 6 months following training - semi-structured interview with leaders	<u>6 month interview guide for leaders</u><br>** Were any adaptations made to MOHMQuit? (What/who/when/why/how?)*
Feasibility (actual fit – the extent to which MOHMQuit can be integrated into usual care in a service)	• Comprehensive and systematic design of MOHMQuit using the Behaviour Change Wheel integrating input from clinicians and leaders[20,21]	Site level	Proctor[33]<br>Rogers[35]	• 3 months following training - questionnaire for leaders<br>• 6 months following training - semi-structured interview with leaders	<u>3 month questionnaire for leaders</u><br>** In the last 3 months did you or any other staff in your service…* (followed by a number of MOHMQuit components)?<br><u>6 month interview guide for leaders</u><br>** Have you, or any of your colleagues implemented any of the components of MOHMQuit* (followed by a number of MOHMQuit components)?

For peer review only - http://bmjopen.bmj.com/site/about/guidelines.xhtml

Implementation outcome (abbreviated definition)	Strategies used to maximise implementation outcomes	Level of analysis	Frameworks used	Data collection instruments, timing, participants	Items from instruments
	• Feasibility and acceptability trial with subsequent minor amendments to the intervention[20]<br>• At the 10 week warm-up the long 'history' of the development of MOHMQuit is described to ensure leaders are reassured about its quality, relevance and acceptability				** How much has MOHMQuit become part of 'usual practice'? (e.g. standard operating procedures, local policies, SCS as a standing item on meeting agendas, audit part of usual audit schedule etc.)*
HOW the implementation of the intervention changed behaviour – the 'mechanisms of impact'+ acceptability, appropriateness and feasibility above		Site level	Moore[32]	• 6 months following training - semi-structured interview with leaders	<u>6 month interview guide for leaders</u><br>** Did MOHMQuit improve the SCS provided to pregnant women in your service? How did it do this?*<br>** How can the implementation of MOHMQuit be improved?*
**HOW context affected implementation**	• **Commitment of maternity service leaders in the**	**Site level**	**Fernandez[36]**	• **Key contextual information (Table 1) completed by research**	**<u>See Table 1 above</u>**<br>*** Birth numbers; smoking prevalence; Performance against the performance indicator of antenatal smoking; Safer Baby**

For peer review only - http://bmjopen.bmj.com/site/about/guidelines.xhtml

Implementation outcome (abbreviated definition)	Strategies used to maximise implementation outcomes	Level of analysis	Frameworks used	Data collection instruments, timing, participants	Items from instruments
	research as Partner Investigators on the grant and as members of the MOHMQuit research Steering Committee and various working groups<br>• Warm-up meetings<br>• Follow up meetings<br>• Community of Practice			team during the implementation<br>• 3 months following training - questionnaire for leaders<br>• 6 months following training - questionnaire with clinicians<br>• 6 months following training - semi-structured interview with leaders	Bundle; RSVP policy; Other SCS initiatives; Accreditation; leadership structure; models of care on offer; other<br><br><u>3 month questionnaire for leaders</u><br>* *Please indicate the extent to which you agree (from Not at all* to *Very great extent)…* all 12 items from the Implementation Leadership Scale[40] e.g. *I have developed a plan to facilitate the implementation of MOHMQuit*<br><u>6 month questionnaire for clinicians</u><br>* *How well do you feel your service leadership has supported the implementation of MOHMQuit* (scale of 1-5 from *Strongly Disagree* to *Strongly* agree) the 4 items of the Implementation Climate measure[36] *e.g. Our service leadership makes sure that we have the time and space necessary to discuss changes to improve care*<br>* *…the general feeling for implementation of MOHMQuit in your service* (scale of 1-5 from *Strongly Disagree* to *Strongly* agree) the 4 items from the Leadership Engagement measure[36] *e.g. Our service staff get the support they need to implement MOHMQuit*<br><u>6 month interview guide for leaders</u><br>* *Has anything changed in terms of your or others' leadership within the service/s around SCS due to MOHMQuit? (Why? How?)*<br>* *What do you think helped in delivering MOHMQuit?*<br>* *What made delivering MOHMQuit more of a challenge?*

For peer review only

For peer review only - http://bmjopen.bmj.com/site/about/guidelines.xhtml

BOLD TYPEFACE INDICATES OUTCOMES THAT WILL BE THE FOCUS OF THE PROCESS EVALUATION

Implementation cost is not included in Table 2 as a detailed economic evaluation of MOHMQuit is taking place and is the subject of a separate paper.[26] Data to contribute to the economic evaluation will be collected as part of the semi-structured interview with leaders.

For peer review only

For peer review only - http://bmjopen.bmj.com/site/about/guidelines.xhtml

Patient and public involvement

As this is an implementation science trial, our partners in identifying the need for the study and in its design and implementation were health service clinicians, leaders and policy makers.[20] Patients were not involved in designing or implementing the research, but are participants in the trial[22] but not in the process evaluation.

Data analysis

We will assess each of the implementation outcomes (Table 2) for each site, including assessing variation across the nine sites. At this stage it is not possible to definitively describe which of the implementation outcomes our analyses will be focused on as that will depend on the variation in implementation outcomes across sites. For example, if there is little variation in fidelity it will not help explain the MOHMQuit (intervention) outcomes. However, where appropriate descriptive statistics (means, standard deviations and proportions) will be produced using data from questionnaire responses from clinicians and leaders to summarise quantitative results by participant and by site.

Analyses for the moderators will include calculation of a mean score for the leadership[40] sub-scales for each participant (four subscales: the proactive subscale, the knowledgeable subscale, the supportive subscale, and the perseverant subscale), a mean score for each set of items that load onto the relevant subscale will be calculated for each subscale. A mean of the scale scores will be calculated which will provide a total score for the Implementation Leadership Scale.[40] In addition, scores will be aggregated to provide a site-level score. We do not anticipate adding these results, or any of the data from Table 1 to any model but they will help constitute a broader assessment of the context for implementation to contribute to understanding of in which sites, and how, MOHMQuit was effective.

Qualitative data from semi-structured interviews with leaders will be analysed descriptively to explore perspectives of uptake by site and thematically across all sites regarding the enablers, barriers and how implementation of MOHMQuit might be improved.[41]

Data from multiple sources will facilitate triangulation. This mixed methods approach will broaden and deepen understanding of the results of the trial. The key findings will be presented in an integrated way using a side-by-side joint display table[42] each source being given equal weight.

Figure 3 below describes this visually.

For peer review only

For peer review only

For peer review only - http://bmjopen.bmj.com/site/about/guidelines.xhtml

Figure 3: Mixed methods approach to data collection and analysis

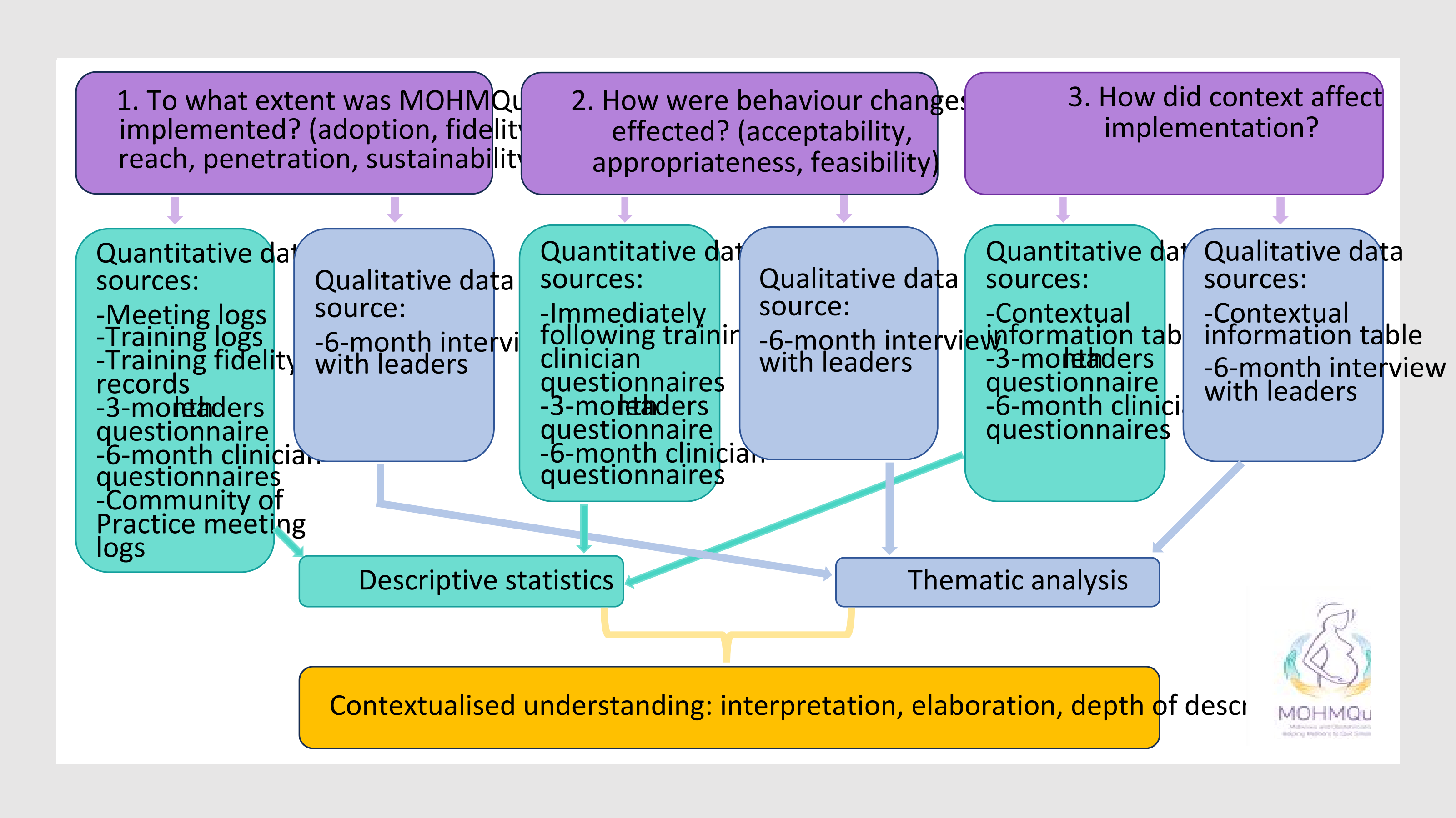

For peer review only - http://bmjopen.bmj.com/site/about/guidelines.xhtml

Ethical considerations and dissemination

The process evaluation received ethical approval from the NSW Population Health Services Research Ethics Committee (Reference Number 2021/ETH00887) on July 23rd, 2021. Results of the process evaluation will be written up for publication in peer-reviewed journals and presented at conferences. The process evaluation will perform a formative function facilitated by the stepped-wedge design (with sites receiving the intervention in a staggered implementation) allowing for further polishing of the intervention as the trial proceeds. The process evaluation will also provide contextual information to elucidate the findings of the trial in terms of how MOHMQuit may have been effective in some sites but not in others. This understanding is critical in relation to rolling out MOHMQuit across NSW should the intervention prove to be effective.

Trial registration number

The MOHMQuit trial is registered with ANZCTR (www.anzctr.org.au): ACTRN12622000167763.

DISCUSSION

Implementation science is the study of approaches that support the systematic uptake of research findings into 'usual care'.[43] In cases where there is an urgent need for behaviour change and a clear evidence to practice gap, such as with SCS in antenatal care, implementation science provides a framework for examining an intervention such as MOHMQuit. This paper describes the mixed-methods design and underpinning frameworks for the process evaluation of MOHMQuit as part of an implementation science study. MOHMQuit is a complex multi-component intervention designed using the Behaviour Change Wheel.[19] It aims to change the behaviour of antenatal care providers to improve the support provided to women to stop smoking in pregnancy. MOHMQuit is being implemented in a stepped-wedge effectiveness trial across nine publicly funded maternity services in NSW.[22]

The process evaluation will facilitate the ongoing refinement of MOHMQuit and will provide an assessment of the extent to which MOHMQuit was implemented, what the mechanisms of impact were and what the context of implementation was, and how it affected the implementation of MOHMQuit. It will also inform other components of the study for example contributing data to support costing of MOHMQuit for the economic evaluation. We anticipate that the findings from the process evaluation will contextualise and aid understanding of our trial results, and may support the further implementation of MOHMQuit in NSW. For example, if it transpires that evidence of implementation leadership is strongly associated with sites where MOHMQuit was shown to be particularly effective, the scale-up would need to include a focus on *implementation leadership* (as measured by the Implementation Leadership Scale),[40] and on implementing the leadership components of the intervention. Our process evaluation will also contribute knowledge about the implementation of stepped-wedge trials which may be useful to others in the future. Whilst we have described our intended approach to evaluating the implementation of MOHMQuit, we have also included flexibility of approach in recognition of unanticipated implementation factors that may surface.[38]

Smoking in pregnancy is an ongoing public health challenge and represents a considerable gap between the evidence for smoking cessation support and practice. Providing a broader understanding of how MOHMQuit was or was not effective will be key to its potential future roll-out/scale up.

For peer review only - http://bmjopen.bmj.com/site/about/guidelines.xhtml

Empirical testing of the theory

Implementation science is a relatively new academic endeavour and this process evaluation has the potential to contribute to a growing body of evidence of approaches to implementing comprehensive stepped-wedge trial designs that are inclusive of process evaluation.

Strengths and limitations

The process evaluation has been designed using implementation science frameworks and explores the implementation of MOHMQuit, a thorough and theoretically underpinned intervention and trial design.[20] The results of the trial will provide further evidence for the effectiveness, or otherwise, of this theoretically driven approach. The mixed methods approach in the process evaluation includes qualitative and quantitative data collection from a wide range of stakeholders (leaders and clinicians) in each MOHMQuit site, some of whom will not have directly participated in the MOHMQuit training, as well as publicly-available data and observational data from the research team implementing MOHMQuit. This approach has the potential to produce findings that have depth and nuance and will aid understanding of the trial findings. However, MOHMQuit is a complex intervention with many moving parts which interact with one another, and the stakeholders involved. No process evaluation is able to collect data to understand all aspects of these interactions. In addition, the MOHMQuit trial is a 'real world' trial. This has strengths in producing findings that can be confidently understood as realistic, however it also produces many challenges including the potential impact of new policies and procedures, staffing issues etc. many of which we have aimed to record as part of the process evaluation but some of which we are likely to have missed. This may compromise our capacity to fully understand and accurately interpret the intervention outcomes

Trial status

Recruitment for the trial is underway. Process evaluation data collection commenced in March 2023 and will conclude in May 2024.

For peer review only

For peer review only - http://bmjopen.bmj.com/site/about/guidelines.xhtml

ACKNOWLEDGEMENTS

This paper is submitted on behalf of the MOHMQuit Trial team, including all chief investigators, partner investigators and associate investigators, and co-researchers and site leads at each of the MOHMQuit sites. In addition to the named authors, the team includes Dheya Al Mashat (NSW Health), Dianne Avery (NSW Health), Elizabeth Best (NSW Ministry of Health), Alecia Brooks (Cancer Council NSW), Rashna Chinoy (NSW Health), Justine Elliot (NSW Health), Jacinta Felsch (NSW Health), Mohamed Foda (NSW Health), Sandra Forde (NSW Health), Tara Farrugia (NSW Health), Tracey Greenberg (Alcohol and Drug Service, St Vincent's Hospital Sydney), Jane Griffith (NSW Health), Madeline Hubbard (NSW Health), Damien McCaul (NSW Ministry of Health), James McLennan (Alcohol and Drug Service, St Vincent's Hospital Sydney), Kate Reakes (Cancer Institute NSW), Virginia Stulz (NSW Health and Western Sydney University), and Tracey Zakazakaarcher (NSW Health).

AUTHORS' CONTRIBUTIONS

The process evaluation was conceived and designed by MP, JL, CP, LT, LB, CA, BB and LA. The first draft of the paper was written by JL with input from MP and CP before receiving input from all other authors. All authors have read and approved the final manuscript.

FUNDING

This work was supported by funding from the NHMRC (GNT1072213) and the Cancer Institute NSW (13/ECF/1-11). MP was supported by a fellowship from the NHMRC (GNT1159601). The funding bodies did not have any role in the design of the study and collection, analysis and interpretation of data.

Ethics approval and consent to participate

Ethics approval for the research was received from the Population Health Services Research Ethics Committee (Reference Number 2021/ETH00887), on July 23rd, 2021. All potential participants will be provided with a Participant Information Sheet (PIS). A signed (written) consent form will be obtained by site trial staff for all maternity service leaders and clinicians who participate in the trial.

Clinicians: Participation of clinicians (anonymous survey participation) is voluntary. Participant Information Sheets for clinicians will explicitly state that the decision to participate or not participate will not influence their professional standing or the care of any of their patients/clients in any way.

Maternity service leaders: The participation of maternity service leaders (semi-structured interviews) is voluntary. The Participant Information Sheet for leaders will explicitly state that the decision to participate or not participate will not influence their professional standing or the care of any of their patients/clients in any way. The Participant Information Sheet will also detail that information shared in interviews will be de-identified before publication or dissemination.

Availability of data and materials

Not applicable

COMPETING INTERESTS

None declared.

For peer review only

For peer review only

References

1. Australian Institue of Health and Welfare. Australia's mothers and babies 2020, web report - Summary Canberra: Australian Government; 2022 [Available from: https://www.aihw.gov.au/reports/mothers-babies/australias-mothers-babies/contents/antenatal-period/smoking-during-pregnancy accessed 23 February 2023.
2. Diamanti A, Papadakis S, Schoretsaniti S, et al. Smoking cessation in pregnancy: An update for maternity care practitioners. *Tobacco induced diseases* 2019;17:57. doi: https://doi.org/10.18332/tid/109906
3. Lange S, Probst C, Rehm J, et al. National, regional, and global prevalence of smoking during pregnancy in the general population: a systematic review and meta-analysis. *The Lancet Global Health* 2018;6(7):e769-e76. doi: https://doi.org/10.1016/S2214-109X(18)30223-7
4. Avşar TS, McLeod H, Jackson L. Health outcomes of smoking during pregnancy and the postpartum period: an umbrella review. *BMC Pregnancy and Childbirth* 2021;21(1):254. doi: 10.1186/s12884-021-03729-1
5. US Department of Health and Human Services. The Health Consequences of Smoking: 50 Years of Progress. A Report of the Surgeon General. Atlanta, GA: U.S. Department of Health and Human Services, Centers for Disease Control and Prevention, National Center for Chronic Disease Prevention and Health Promotion, Office on Smoking and Health, 2014.
6. U.S. Department of Health & Human Services. Smoking Cessation: A Report of the Surgeon General. Rockville: US Department of Health and Human Services, 2020.
7. NSW Ministry of Health. The First 2000 Days Framework. In: Health and Social Policy Branch, ed. Sydney, Australia, 2019.
8. NSW Ministry of Health. First 2000 Days Implementation Strategy 2020-2025. St Leonards: NSW Ministry of Health, 2021.
9. NSW Ministry of Health. NSW Clinical Guidelines for the Management of Substance Use During Pregnancy, Birth and the Postnatal Period. Sydney: NSW Ministry of Health, 2014.
10. Passey ME, Longman JM, Adams CM, et al. Factors associated with provision of smoking cessation support to pregnant women – a cross-sectional survey of midwives in New South Wales, Australia. *BMC Pregnancy and Childbirth* 2020;20:219.
11. Longman JM, Adams CM, Johnston JJ, et al. Improving implementation of the smoking cessation guidelines with pregnant women: How to support clinicians? *Midwifery* 2018;58:137-44. doi: https://doi.org/10.1016/j.midw.2017.12.016
12. Passey M, Sanson-Fisher RW. Provision of antenatal smoking cessation support: a survey with pregnant Aboriginal and Torres Strait Islander women. *Nicotine Tob Res* 2015;17(6):746-49.
13. Bar Zeev Y, Bonevski B, Twyman L, et al. Opportunities Missed: A Cross-sectional Survey of the Provision of Smoking Cessation Care to Pregnant Women by Australian General Practitioners and Obstetricians. *Nicotine & tobacco research: official journal of the Society for Research on Nicotine and Tobacco* 2017;19(5):636-41.
14. Hoekzema L, Werumeus Buning A, Bonevski B, et al. Smoking rates and smoking cessation preferences of pregnant women attending antenatal clinics of two large Australian maternity hospitals. *Aust N Z J Obstet Gynaecol* 2014;54(1):53-8.
15. Passey ME, Sanson-Fisher RW, Stirling JM. Supporting pregnant Aboriginal and Torres Strait Islander women to quit smoking: views of antenatal care providers and pregnant Indigenous women. *Matern Child Health J* 2014;18:2293-99.
16. Chamberlain C, O'Mara-Eves A, Porter J, et al. Psychosocial interventions for supporting women to stop smoking in pregnancy. *The Cochrane Library* 2017(2) doi: 10.1002/14651858.CD001055.pub5.
17. Skivington K, Matthews L, Simpson SA, et al. A new framework for developing and evaluating complex interventions: update of Medical Research Council guidance. *bmj* 2021;374
18. U.S. Department of Health and Human Services. Smoking Cessation. A Report of the Surgeon General. Atlanta GA: U.S. Department of Health and Human Services, Centers for Disease

For peer review only - http://bmjopen.bmj.com/site/about/guidelines.xhtml

Control and Prevention, National Center for Chronic Disease Prevention and Health Promotion, Office on Smoking and Health, 2020.
19. Michie S, van Stralen MM, West R. The behaviour change wheel: A new method for characterising and designing behaviour change interventions. *Implement Sci* 2011;6:42.
20. Passey ME, Adams C, Paul C, et al. Improving implementation of smoking cessation guidelines in pregnancy care: Development of an intervention to address system, maternity service leader and clinician factors. *Implementation Science Communications* 2021;2:128. doi: https://doi.org/10.1186/s43058-021-00235-5
21. Longman J, Adams C, Paul C, et al. Improving clinicians' implementation of guidelines to help women stop smoking in pregnancy: developing evidence-based print and video materials. *Int J Environ Res Public Health* 2021;18(19):10522. doi: https://doi.org/10.3390/ijerph181910522
22. Barnes LAJ, Longman J, Adams C, et al. The MOHMQuit (Midwives and Obstetricians Helping Mothers to Quit Smoking) Trial: protocol for a stepped-wedge implementation trial to improve best practice smoking cessation support in public antenatal care services. *Implement Sci* 2022;17(1):79. doi: 10.1186/s13012-022-01250-3
23. Lewis CC, Boyd MR, Walsh-Bailey C, et al. A systematic review of empirical studies examining mechanisms of implementation in health. *Implement Sci* 2020;15:1-25.
24. Lewis CC, Klasnja P, Powell BJ, et al. From classification to causality: advancing understanding of mechanisms of change in implementation science. *Frontiers in public health* 2018;6:136.
25. Springer MV, Sales AE, Islam N, et al. A step toward understanding the mechanism of action of audit and feedback: a qualitative study of implementation strategies. *Implement Sci* 2021;16:1-13.
26. Pearce A, Scarfe J, Jones M, et al. Study protocol of an economic evaluation embedded in the Midwives and Obstetricians Helping Mothers to Quit Smoking (MOHMQuit) trial. *BMC Health Serv Res* 2023;23(1):1-9.
27. Graham Moore SA, Barker M, Bond L, et al. Process evaluation of complex interventions. *Complex Interventions in Health: An overview of research methods* 2015:222.
28. Glasgow RE, Vogt TM, Boles SM. Evaluating the public health impact of health promotion interventions: the RE-AIM framework. *Am J Public Health* 1999;89(9):1322-27.
29. Global Alliance for Chronic Diseases. GACD Process Evaluation Guidelines, 2015 [Available from: https://www.gacd.org/perch/resources/gacd-pe-guidelines-v1-0.pdf.
30. Hawe P, Shiell A, Riley T. Complex interventions: how "out of control" can a randomised controlled trial be? *Bmj* 2004;328(7455):1561-63.
31. Kirk MA, Moore JE, Wiltsey Stirman S, et al. Towards a comprehensive model for understanding adaptations' impact: the model for adaptation design and impact (MADI). *Implement Sci* 2020;15:1-15.
32. Moore GF, Audrey S, Barker M, et al. Process evaluation of complex interventions: Medical Research Council guidance. *bmj* 2015;350
33. Proctor E, Silmere H, Raghavan R, et al. Outcomes for implementation research: conceptual distinctions, measurement challenges, and research agenda. *Administration and policy in mental health and mental health services research* 2011;38(2):65-76.
34. Sekhon M, Cartwright M, Francis JJ. Acceptability of healthcare interventions: an overview of reviews and development of a theoretical framework. *BMC Health Serv Res* 2017;17(1):88.
35. Rogers EM. Diffusion of Innovations. 4th ed. New York: The Free Press 1995.
36. Fernandez ME, Walker TJ, Weiner BJ, et al. Developing measures to assess constructs from the Inner Setting domain of the Consolidated Framework for Implementation Research. *Implement Sci* 2018;13(1):52. doi: 10.1186/s13012-018-0736-7
37. Craig P, Di Ruggiero E, Frolich KL, et al. Taking account of context in population health intervention research: guidance for producers, users and funders of research. . Canadian

For peer review only

Institutes of Health Research (CIHR)–National Institute for Health Research (NIHR) Southampton: National Institute for Health Research, 2018.

38. Beames JR, Lingam R, Boydell K, et al. Protocol for the process evaluation of a complex intervention delivered in schools to prevent adolescent depression: the Future Proofing Study. *BMJ open* 2021;11(1):e042133.
39. Moullin JC, Sklar M, Ehrhart MG, et al. Provider REport of Sustainment Scale (PRESS): development and validation of a brief measure of inner context sustainment. *Implement Sci* 2021;16(1):1-10.
40. Aarons GA, Ehrhart MG, Farahnak LR. The Implementation Leadership Scale (ILS): Development of a Brief Measure of Unit Level Implementation Leadership. *Implement Sci* 2014;9:45. doi: 10.1186/1748-5908-9-45
41. Braun V, Clarke V. Using thematic analysis in psychology. *Qualitative research in psychology* 2006;3(2):77-101.
42. Guetterman TC, Fetters MD, Creswell JW. Integrating quantitative and qualitative results in health science mixed methods research through joint displays. *The Annals of Family Medicine* 2015;13(6):554-61.
43. Eccles M, Mittman B. Welcome to implementation science [Internet]. Vol. 1. *Implementation Science BioMed Central* 2006:1.

For peer review only

**Antenatal Care clinicians**	• Antenatal care midwives<br>• Aboriginal Health Workers (AHWs) - primary healthcare workers who ensure culturally safe maternity care in supporting Aboriginal and/or Torres Strait Islander women or women having an Aboriginal baby<br>• Obstetricians (staff specialists; Visiting Medical Officers with specialist obstetric training, Career Medical Officers) and obstetric registrars
**Leaders**	Maternity service leaders (those who support or supervise health professionals providing antenatal care), including:<br>• Clinical Midwifery Consultants<br>• Maternity Unit Managers<br>• Clinical Midwifery Educators<br>• Clinical Midwifery Specialists<br>• Antenatal clinic coordinators<br>• Obstetric leads

For peer review only

	Implementation outcomes							
**Context measures**	Acceptability	Adoption	Appropriateness	Feasibility	Fidelity	Penetration	Sustainability	Reach
Leadership								
Implementation climate								
Service size								
Smoking prevalence								
Models of care								
Other demands on leaders								

For peer review only

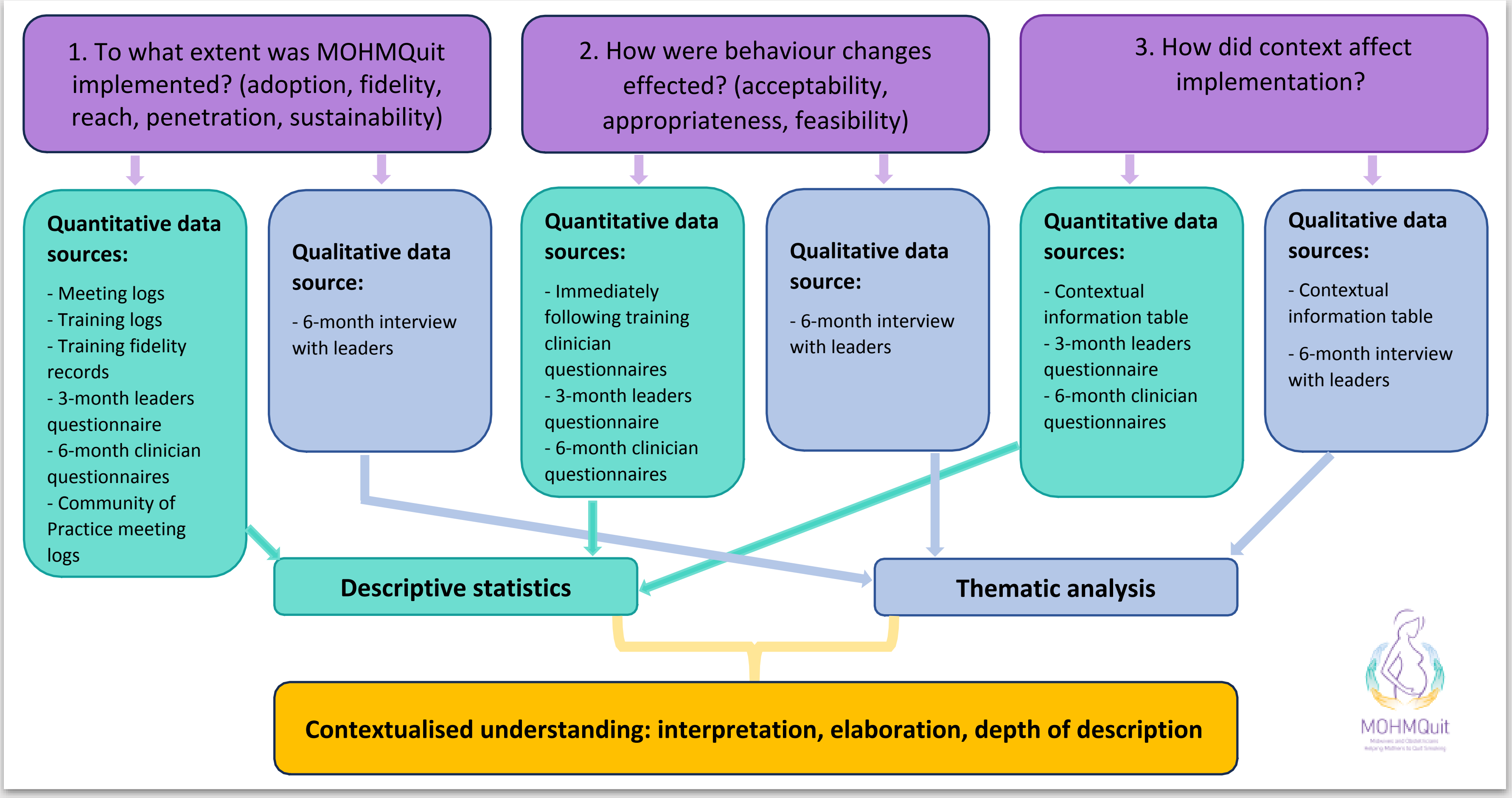
1. To what extent was MOHMQuit implemented? (adoption, fidelity, reach, penetration, sustainability)
2. How were behaviour changes effected? (acceptability, appropriateness, feasibility)
3. How did context affect implementation?
Quantitative data sources:
- Meeting logs
- Training logs
- Training fidelity records
- 3-month leaders questionnaire
- 6-month clinician questionnaires
- Community of Practice meeting logs
Qualitative data source:
- 6-month interview with leaders
Quantitative data sources:
- Immediately following training clinician questionnaires
- 3-month leaders questionnaire
- 6-month clinician questionnaires
Qualitative data source:
- 6-month interview with leaders
Quantitative data sources:
- Contextual information table
- 3-month leaders questionnaire
- 6-month clinician questionnaires
Qualitative data sources:
- Contextual information table
- 6-month interview with leaders
Descriptive statistics
Thematic analysis
Contextualised understanding: interpretation, elaboration, depth of description
MOHMQuit

SPIRIT
STANDARD PROTOCOL ITEMS: RECOMMENDATIONS FOR INTERVENTIONAL TRIALS

SPIRIT 2013 Checklist: Recommended items to address in a clinical trial protocol and related documents*

Section/item	Item No	Description
**Administrative information**		
Title	1	Descriptive title identifying the study design, population, interventions, and, if applicable, trial acronym<br>***Protocol for the process evaluation of an intervention to improve antenatal smoking cessation support (MOHMQuit) in maternity services in New South Wales, Australia***
Trial registration	2a	Trial identifier and registry name. If not yet registered, name of intended registry<br>***The MOHMQuit trial is registered (ACTRN12622000167763 https://www.australianclinicaltrials.gov.au/anzctr/trial/ACTRN12622000167763)***
	2b	All items from the World Health Organization Trial Registration Data Set<br>**N/A**
Protocol version	3	Date and version identifier<br>**The protocol for the whole MOHMQuit trial (which includes the process evaluation) is a published paper (Barnes, L. A. J., J. Longman, C. Adams, C. Paul, L. Atkins, B. Bonevski, A. Cashmore, L. Twyman, R. Bailie, A. Pearce, D. Barker, A. J. Milat, J. Dorling, M. Nicholl and M. Passey (2022). "The MOHMQuit (Midwives and Obstetricians Helping Mothers to Quit Smoking) Trial: protocol for a stepped-wedge implementation trial to improve best practice smoking cessation support in public antenatal care services." Implementation Science 17(1): 79). This paper itself is a more detailed protocol for the process evaluation.**
Funding	4	Sources and types of financial, material, and other support<br>***This work was supported by funding from the NHMRC (GNT1072213) and the Cancer Institute NSW (13/ECF/1-11). MP was supported by a fellowship from the NHMRC (GNT1159601).***

For peer review only

Roles and responsibilities	5a	Names, affiliations, and roles of protocol contributors<br>***Jo Longman[1], Chris Paul[2], Aaron Cashmore[3,4], Laura Twyman[5], Larisa AJ Barnes[1], Cathy Adams[6], Billie Bonevski[7], Andrew Milat[3,4] and Megan E Passey[1]* (affiliations are listed on the title page of the paper).**<br>***The process evaluation was conceived and designed by MP, JL, CP, LT, LB, CA, BB and LA. The first draft of the paper was written by JL with input from MP and CP before receiving input from all other authors. All authors have read and approved the final manuscript.***
	5b	Name and contact information for the trial sponsor<br>N/A
	5c	Role of study sponsor and funders, if any, in study design; collection, management, analysis, and interpretation of data; writing of the report; and the decision to submit the report for publication, including whether they will have ultimate authority over any of these activities<br>**The funding bodies did not have any role in the design of the study and collection, analysis and interpretation of data.**
	5d	Composition, roles, and responsibilities of the coordinating centre, steering committee, endpoint adjudication committee, data management team, and other individuals or groups overseeing the trial, if applicable (see Item 21a for data monitoring committee)<br>N/A
**Introduction**		
Background and rationale	6a	Description of research question and justification for undertaking the trial, including summary of relevant studies (published and unpublished) examining benefits and harms for each intervention<br>**Includes research questions driving the process evaluation and justification for the process evaluation – see paragraph *"Aims of the MOHMQuit process evaluation"*.**
	6b	Explanation for choice of comparators<br>N/A
Objectives	7	Specific objectives or hypotheses<br>**See paragraph *"Overall design and objectives of the process evaluation"*.**

For peer review only - http://bmjopen.bmj.com/site/about/guidelines.xhtml

Trial design	8	Description of trial design including type of trial (eg, parallel group, crossover, factorial, single group), allocation ratio, and framework (eg, superiority, equivalence, noninferiority, exploratory)<br>***MOHMQuit is currently being trialled in a multi-site cluster randomised stepped-wedge effectiveness trial in nine sites in publicly-funded maternity services in NSW, Australia***

Methods: Participants, interventions, and outcomes

Study setting	9	Description of study settings (eg, community clinic, academic hospital) and list of countries where data will be collected. Reference to where list of study sites can be obtained<br>**MOHMQuit is currently being trialled in a multi-site cluster randomised stepped-wedge effectiveness trial in nine sites in publicly-funded maternity services in NSW, Australia**.
Eligibility criteria	10	Inclusion and exclusion criteria for participants. If applicable, eligibility criteria for study centres and individuals who will perform the interventions (eg, surgeons, psychotherapists)<br>**See "Recruitment and Consent" section**
Interventions	11a	Interventions for each group with sufficient detail to allow replication, including how and when they will be administered<br>**The MOHMQuit intervention has been described in detail in a previously published manuscript so is described in brief here. See "The MOHMQuit Intervention" section**
	11b	Criteria for discontinuing or modifying allocated interventions for a given trial participant (eg, drug dose change in response to harms, participant request, or improving/worsening disease)<br>N/A
	11c	Strategies to improve adherence to intervention protocols, and any procedures for monitoring adherence (eg, drug tablet return, laboratory tests)<br>N/A
	11d	Relevant concomitant care and interventions that are permitted or prohibited during the trial<br>N/A

For peer review only - http://bmjopen.bmj.com/site/about/guidelines.xhtml

Outcomes	12	Primary, secondary, and other outcomes, including the specific measurement variable (eg, systolic blood pressure), analysis metric (eg, change from baseline, final value, time to event), method of aggregation (eg, median, proportion), and time point for each outcome. Explanation of the clinical relevance of chosen efficacy and harm outcomes is strongly recommended<br>**Primary and secondary intervention outcomes are described in brief:** ***The primary intervention outcome is smoking cessation, and secondary intervention outcomes include changes to clinicians' knowledge, skills, confidence and behaviour in providing SCS and test the 'mechanisms of action' by which each of the components/strategies effect intervention outcomes and moderators of their impact in this framework-driven approach. Cost-effectiveness will be assessed in an economic evaluation.*** **The implementation outcomes (the process evaluation) are described in detail – see "Overall design and objectives of the process evaluation" section and Table 4**
Participant timeline	13	Time schedule of enrolment, interventions (including any run-ins and washouts), assessments, and visits for participants. A schematic diagram is highly recommended (see Figure)<br>**The timeline for the process evaluation is described in the "Trial status" paragraph**.
Sample size	14	Estimated number of participants needed to achieve study objectives and how it was determined, including clinical and statistical assumptions supporting any sample size calculations<br>N/A
Recruitment	15	Strategies for achieving adequate participant enrolment to reach target sample size<br>N/A

Methods: Assignment of interventions (for controlled trials)

Allocation:

Sequence generation	16a	Method of generating the allocation sequence (eg, computer-generated random numbers), and list of any factors for stratification. To reduce predictability of a random sequence, details of any planned restriction (eg, blocking) should be provided in a separate document that is unavailable to those who enrol participants or assign interventions<br>N/A
Allocation concealment mechanism	16b	Mechanism of implementing the allocation sequence (eg, central telephone; sequentially numbered, opaque, sealed envelopes), describing any steps to conceal the sequence until interventions are assigned<br>N/A

Implementation	16c	Who will generate the allocation sequence, who will enrol participants, and who will assign participants to interventions<br>N/A
Blinding (masking)	17a	Who will be blinded after assignment to interventions (eg, trial participants, care providers, outcome assessors, data analysts), and how<br>N/A
	17b	If blinded, circumstances under which unblinding is permissible, and procedure for revealing a participant's allocated intervention during the trial<br>N/A
**Methods: Data collection, management, and analysis**		
Data collection methods	18a	Plans for assessment and collection of outcome, baseline, and other trial data, including any related processes to promote data quality (eg, duplicate measurements, training of assessors) and a description of study instruments (eg, questionnaires, laboratory tests) along with their reliability and validity, if known. Reference to where data collection forms can be found, if not in the protocol<br>**This is described in the "Process evaluation data collection" section**
	18b	Plans to promote participant retention and complete follow-up, including list of any outcome data to be collected for participants who discontinue or deviate from intervention protocols<br>**Plans to keep MOHMQuit at the forefront of clinicians' and leaders' minds (from whom data will be collected 6 months following the intervention) include those addressing sustainability of the intervention: MOHMQuit leadership components; the 'train the trainer' model; and the Community of Practice (Table 4)**
Data management	19	Plans for data entry, coding, security, and storage, including any related processes to promote data quality (eg, double data entry; range checks for data values). Reference to where details of data management procedures can be found, if not in the protocol<br>**This detail is included in the ethics application for the trial (which includes the process evaluation) and for the sake of brevity are not included in this manuscript.**
Statistical methods	20a	Statistical methods for analysing primary and secondary outcomes. Reference to where other details of the statistical analysis plan can be found, if not in the protocol<br>**This is described in the "Data analysis" section**

For peer review only - http://bmjopen.bmj.com/site/about/guidelines.xhtml

	20b	Methods for any additional analyses (eg, subgroup and adjusted analyses)<br>N/A
	20c	Definition of analysis population relating to protocol non-adherence (eg, as randomised analysis), and any statistical methods to handle missing data (eg, multiple imputation)<br>N/A
**Methods: Monitoring**		
Data monitoring	21a	Composition of data monitoring committee (DMC); summary of its role and reporting structure; statement of whether it is independent from the sponsor and competing interests; and reference to where further details about its charter can be found, if not in the protocol. Alternatively, an explanation of why a DMC is not needed<br>N/A
	21b	Description of any interim analyses and stopping guidelines, including who will have access to these interim results and make the final decision to terminate the trial<br>N/A
Harms	22	Plans for collecting, assessing, reporting, and managing solicited and spontaneously reported adverse events and other unintended effects of trial interventions or trial conduct<br>N/A
Auditing	23	Frequency and procedures for auditing trial conduct, if any, and whether the process will be independent from investigators and the sponsor<br>N/A
**Ethics and dissemination**		
Research ethics approval	24	Plans for seeking research ethics committee/institutional review board (REC/IRB) approval<br>***Ethics approval for the research was received from the Population Health Services Research Ethics Committee (Reference Number 2021/ETH00887), on July 23rd, 2021***.
Protocol amendments	25	Plans for communicating important protocol modifications (eg, changes to eligibility criteria, outcomes, analyses) to relevant parties (eg, investigators, REC/IRBs, trial participants, trial registries, journals, regulators)<br>N/A
Consent or assent	26a	Who will obtain informed consent or assent from potential trial participants or authorised surrogates, and how (see Item 32)<br>**This is described in the section "Ethical approval and consent to participate"**

only

For peer review only - http://bmjopen.bmj.com/site/about/guidelines.xhtml

	26b	Additional consent provisions for collection and use of participant data and biological specimens in ancillary studies, if applicable<br>N/A
Confidentiality	27	How personal information about potential and enrolled participants will be collected, shared, and maintained in order to protect confidentiality before, during, and after the trial<br>**This is described in the section "Ethical approval and consent to participate"**
Declaration of interests	28	Financial and other competing interests for principal investigators for the overall trial and each study site<br>**There is a "Competing Interests" statement and a ICMJE Disclosure Form submitted with the manuscript**
Access to data	29	Statement of who will have access to the final trial dataset, and disclosure of contractual agreements that limit such access for investigators<br>N/A
Ancillary and post-trial care	30	Provisions, if any, for ancillary and post-trial care, and for compensation to those who suffer harm from trial participation<br>N/A
Dissemination policy	31a	Plans for investigators and sponsor to communicate trial results to participants, healthcare professionals, the public, and other relevant groups (eg, via publication, reporting in results databases, or other data sharing arrangements), including any publication restrictions<br>**This is described in the "Ethics and dissemination" section**
	31b	Authorship eligibility guidelines and any intended use of professional writers<br>**This level of detail has not been included for the sake of brevity. Professional writers will not be used.**
	31c	Plans, if any, for granting public access to the full protocol, participant-level dataset, and statistical code<br>N/A
**Appendices**		
Informed consent materials	32	Model consent form and other related documentation given to participants and authorised surrogates<br>**This level of detail has not been included for the sake of brevity.**
Biological specimens	33	Plans for collection, laboratory evaluation, and storage of biological specimens for genetic or molecular analysis in the current trial and for future use in ancillary studies, if applicable<br>N/A

*It is strongly recommended that this checklist be read in conjunction with the SPIRIT 2013 Explanation & Elaboration for important clarification on the items. Amendments to the

For peer review only - http://bmjopen.bmj.com/site/about/guidelines.xhtml

protocol should be tracked and dated. The SPIRIT checklist is copyrighted by the SPIRIT Group under the Creative Commons "Attribution-NonCommercial-NoDerivs 3.0 Unported" license.

For peer review only

BMJ Open

Protocol for the process evaluation of an intervention to improve antenatal smoking cessation support (MOHMQuit) in maternity services in New South Wales, Australia

Journal:	*BMJ Open*
Manuscript ID	bmjopen-2023-081208.R1
Article Type:	Protocol
Date Submitted by the Author:	19-Feb-2024
Complete List of Authors:	Longman, Jo; The University of Sydney, University Centre for Rural Health<br>Paul, Christine; University of Newcastle, School of Medicine and Public Health<br>Cashmore, Aaron; NSW Health, Centre for Epidemiology and Evidence; The University of Sydney, School of Public Health, Faculty of Medicine and Health<br>Twyman, Laura; Cancer Council NSW; The University of Newcastle, School of Medicine and Public Health<br>Barnes, Larisa; The University of Sydney, University Centre for Rural Health<br>Adams, Catherine; NSW Health, Clinical Excellence Commission<br>Bonevski, Billie; Flinders University, College of Medicine & Public Health<br>Milat, Andrew; The University of Sydney, School of Public Health<br>Passey, Megan; The University of Sydney, Daffodil Centre and the University Centre for Rural Health, Faculty of Medicine and Health
**Primary Subject Heading**:	Public health
Secondary Subject Heading:	Research methods
Keywords:	Implementation Science, Behavior, Primary Health Care, Pregnant Women, Change management < HEALTH SERVICES ADMINISTRATION & MANAGEMENT, Quality in health care < HEALTH SERVICES ADMINISTRATION & MANAGEMENT

SCHOLARONE™
Manuscripts

Protocol for the process evaluation of an intervention to improve antenatal smoking cessation support (MOHMQuit) in maternity services in New South Wales, Australia

Jo Longman[1], Chris Paul[2], Aaron Cashmore[3,4], Laura Twyman[5], Larisa AJ Barnes[1,6], Cathy Adams[7], Billie Bonevski[8], Andrew Milat[3,4] and Megan E Passey[1,6]

[1] The University of Sydney, The University Centre for Rural Health, 61 Uralba St., Lismore, NSW 2480, Australia.
[2] University of Newcastle, School of Medicine and Public Health, University Drive, Callaghan, NSW 2308, Australia.
[3] NSW Ministry of Health, Centre for Epidemiology and Evidence, 1 Reserve Road, St Leonards, NSW 2065, Australia.
[4] The University of Sydney, Sydney School of Public Health, Faculty of Medicine and Health, Camperdown, NSW 2006, Australia.
[5] Tobacco Control Unit, Cancer Prevention and Advocacy Division, Cancer Council NSW, and Conjoint Fellow, School of Medicine and Public Health, University of Newcastle, 153 Dowling St., Woolloomooloo, NSW 2011, Australia
[6] The University of Sydney, Daffodil Centre, PO Box 572 Kings Cross NSW 1340, Australia.
[7] Maternity and Neonatal Safety Program; Clinical Excellence Commission, 1 Reserve Road, St Leonards, NSW 2065
[8] Flinders University, College of Medicine & Public Health, Flinders Health and Medical Research Institute, Sturt Road, Bedford Park, SA 5042, Australia

Corresponding author:

Larisa A.J. Barnes
larisa.barnes@sydney.edu.au
Daffodil Centre and the University Centre for Rural Health
Faculty of Medicine and Health
The University of Sydney

For peer review only

For peer review only - http://bmjopen.bmj.com/site/about/guidelines.xhtml

ABSTRACT

Introduction

Smoking cessation in pregnancy remains a public health priority. Our team used the Behaviour Change Wheel to develop the MOHMQuit intervention (Midwives and Obstetricians Helping Mothers to Quit smoking) with health system, leader (including managers and educators) and clinician components. MOHMQuit addresses a critical evidence to practice gap in the provision of smoking cessation support in antenatal care. It involves nine maternity services in New South Wales in a cluster randomised stepped-wedge controlled trial of effectiveness. This paper describes the design and rationale for the process evaluation of MOHMQuit. The process evaluation aims to assess to what extent and how MOHMQuit is being implemented (acceptability; adoption/uptake; appropriateness; feasibility; fidelity; penetration and sustainability), and the context in which it is implemented, in order to support further refinement of MOHMQuit throughout the trial, and aid understanding and interpretation of the results of the trial.

Methods and analysis

The process evaluation is an integral part of the stepped-wedge trial. Its design is underpinned by implementation science frameworks and adopts a mixed methods approach. Quantitative evidence from participating leaders and clinicians in our study will be used to produce individual and site-level descriptive statistics. Qualitative evidence of leaders' perceptions about the implementation will be collected using semi-structured interviews and will be analysed descriptively within-site and thematically across the dataset. The process evaluation will also use publicly-available data and observations from the research team implementing MOHMQuit e.g. training logs. These data will be synthesised to provide site-level as well as individual-level implementation outcomes.

Ethics and dissemination

The study received ethical approval from the Population Health Services Research Ethics Committee for NSW, Australia (Reference 2021/ETH00887). Results will be communicated via the study's Steering Committee and will also be published in peer-reviewed journals and presented at conferences.

Trial registration

Australian New Zealand Trials Registry ACTRN12622000167763
https://www.australianclinicaltrials.gov.au/anzctr/trial/ACTRN12622000167763

Keywords

Implementation science, Behavior, Primary health care, Smoking cessation support, Pregnancy, Antenatal care, Systems change intervention, Stepped-wedge cluster-randomised controlled trial; evaluation studies as topic; process evaluation

For peer review only - http://bmjopen.bmj.com/site/about/guidelines.xhtml

Strengths and limitations of this study

► The process evaluation has been designed using implementation science frameworks

► The study uses multiple data sources. Qualitative and quantitative data will be collected independently from leaders and clinicians in each MOHMQuit site as well as contextual and publicly-available data, and observational data from the research team implementing MOHMQuit

► MOHMQuit is a complex intervention with many moving parts which interact with one another and the stakeholders involved. No process evaluation is able to collect data to understand all aspects of these interactions, particularly not in a ‘real world’ trial such as this one.

For peer review only

For peer review only - http://bmjopen.bmj.com/site/about/guidelines.xhtml

INTRODUCTION

In 2020, 9.2% of mothers in Australia smoked tobacco at some point during their pregnancy.(1) Smoking in pregnancy is associated with a multitude of adverse outcomes for both mother and baby including pre-term birth and low birth weight babies.(2-5) In Australia, smoking is the most common modifiable risk for adverse pregnancy and birth outcomes(6) and therefore supporting pregnant women to stop smoking remains a major public health concern and a priority for the New South Wales (NSW) Ministry of Health.(7-9) Clinical guidelines for NSW have existed for almost 20 years and recommend clinicians routinely provide evidence-based smoking cessation support (SCS) at all antenatal care visits for women who smoke or who have stopped smoking in this pregnancy.(10) Implementation of the Guidelines shows room for improvement.(11-14) This fact, along with wider evidence that women want to stop smoking in pregnancy but some lack confidence to do so, (15) would value support from their clinicians(16) and a systematic review demonstrating that psychosocial interventions helps women to stop smoking, (17) led us to develop a theoretically underpinned intervention, MOHMQuit (Midwives and Obstetricians Helping Mothers to Quit smoking) to improve implementation of the NSW Guidelines.

The MOHMQuit intervention

The MOHMQuit intervention has multiple components targeting different parts of a complex health system.(18) It is based on the '5As' of SCS: Ask, Advise, Assess, Assist and Arrange Follow-up, which has shown evidence of effectiveness for SCS.(19) MOHMQuit was developed using the Behaviour Change Wheel method.(20) It is an intervention built on local and international evidence identifying barriers and enablers for health systems, leaders and clinicians providing SCS.(21) It focuses on changing behaviours by targeting systems such as the electronic medical record system, leaders and clinicians (see Figure 1 for further detail on what is meant by leaders and clinicians). For example, changing clinicians' behaviours so that they implement the Guidelines by asking about smoking and discussing cessation at every antenatal visit, and assisting women by providing behavioural support such as discussing triggers for smoking, managing nicotine cravings, and planning a quit attempt. The MOHMQuit trial is an implementation trial using a stepped-wedge design across five Local Health Districts in NSW with diverse characteristics including organisational structure and staffing profiles.

Figure 1: Description of key participant groups

The development of the MOHMQuit intervention and its support materials have been described in detail previously.(21 22) In brief, there are four main components (also referred to in the implementation science literature as 'implementation strategies'):

(1) separate training events for: maternity service leaders - half day, midwives and AHWs - full day and obstetricians - two hours. Midwifery educators also take part in the leaders' and midwives' training events as a 'train the trainer' model which includes a comprehensive MOHMQuit training manual, is central to the sustainability of the intervention;

(2) a number of MOHMQuit leadership processes and systems tools e.g. a report template for the electronic medical record system facilitating leaders' scrutiny of their services' SCS performance; a service audit tool for leaders;

(3) MOHMQuit written resources such as a booklet on 'Stopping smoking for you and your baby' for clinicians to use with women; and

For peer review only - http://bmjopen.bmj.com/site/about/guidelines.xhtml

(4) a series of 11 short video clips for training and skills development to be used in a wide variety of settings e.g. at handover meetings.

Two months prior to the implementation starting in the first site, a day-long face to face gathering was held bringing together key decision makers and clinicians from across the sites to ensure a shared awareness and understanding of MOHMQuit including its history and rationale, promote enthusiasm, motivation and engagement and establish shared understanding about roles and responsibilities.

At each site, ten weeks prior to the intervention the research team and the maternity service leaders will participate in a 'warm-up' meeting. Whilst each site has a strong existing connection with MOHMQuit via the face to face day, and through the inclusion of partner investigators at each site, the warm-up meeting includes: acknowledging and thanking those involved (which extend beyond the site partner investigators and include the antenatal clinic coordinator, the clinical midwifery educator and other leaders), generating enthusiasm, building momentum in the lead up to the implementation of MOHMQuit, and working through the logistics of implementation at each site. Two weeks prior to the intervention a second meeting will be held which has a 'trouble-shooting' agenda and will also include detail of the research elements of MOHMQuit for example how and when outcome and process evaluation data from the site will be collected. Additional meetings are planned for two and four weeks post-intervention, to maintain momentum and explore any unresolved issues in the ongoing implementation of MOHMQuit. A MOHMQuit Community of Practice will be established which each site can join following implementation. The Community of Practice will offer a regular forum for sharing and supporting other clinicians and leaders in continuing to implement MOHMQuit and is one of several sustainability features of MOHMQuit. Finally, three and a half months after implementation, each site will receive feedback from brief interviews with women about the smoking cessation support they received during their antenatal care. They will continue to receive these reports quarterly until the end of the trial.

MOHMQuit is currently being trialled in a multi-site cluster randomised stepped-wedge effectiveness trial in nine sites in publicly-funded maternity services in NSW, Australia.(23) Implementation is planned to take place over a 13 month timeframe. Unlike many earlier interventions aimed at improving SCS,(24) MOHMQuit is built on implementation science frameworks and is specific to the public maternity service setting. The trial will assess the intervention outcomes. The primary intervention outcome is smoking cessation, and secondary intervention outcomes include changes to clinicians' knowledge, skills, confidence and behaviour in providing SCS and test the 'mechanisms of action'(25-27) by which each of the components effect intervention outcomes and moderators of their impact in this framework-driven approach.(23) Cost-effectiveness will be assessed in an economic evaluation.(28) The trial will also assess key implementation outcomes (assessing how MOHMQuit was implemented) primarily based on Proctor et al's implementation science framework(29) in a detailed process evaluation. The process evaluation will complement the assessment of the MOHMQuit intervention outcomes. Conducting process evaluation alongside effectiveness trials in this way is recommended.(30 31)

Aims of the MOHMQuit process evaluation

Process evaluations explore how an intervention is implemented. They assess three aspects: (a) how and to what extent the intervention was implemented; (b) the 'mechanisms of impact' i.e. how the intervention components and participants' interactions with these components effected changes in behaviour; and (c) the context in which the intervention was implemented.(32) We anticipate that the process evaluation will contribute formatively by providing feedback that may further refine the

For peer review only - http://bmjopen.bmj.com/site/about/guidelines.xhtml

intervention. This is particularly useful in a stepped-wedge trial design where each site joins the trial sequentially, and acceptable as long as the changes made to components retain the integrity of the function they were meant to perform in the original intervention design.(33 34) The summative use of process evaluation is in providing insight into the mechanisms through which the intervention outcomes (the primary intervention outcome being pregnant women stopping smoking), were achieved or not, and therefore it will contribute to understanding and interpreting the results of the effectiveness trial.(35) Without this insight effective, and ineffective, aspects of the intervention may not be understood and this has implications for the scale-up of an intervention such as MOHMQuit. In this way, the process evaluation will maximise the knowledge gained throughout the trial and describe the most effective delivery processes for the MOHMQuit intervention. The aim of this protocol paper is to describe the process evaluation planned as an integral part of the MOHMQuit trial.

METHODS AND ANALYSIS

Overall design and objectives of the process evaluation

The design for the process evaluation began with the implementation outcomes defined by Enola Proctor and team in order to facilitate an understanding of the various dimensions of the implementation: acceptability; adoption/uptake; appropriateness; feasibility; fidelity; penetration and sustainability (and sustainment).(29) Implementation outcomes are "…the effects of deliberate and purposive actions to implement new…practices".(29) The Proctor implementation outcomes generally map on to other well-used frameworks such as the RE-AIM (Reach, Efficacy, Adoption, Implementation, Maintenance) framework (31) but 'Reach' from the RE-AIM framework was specifically added into the design as 'Reach' captures the number of clinicians and leaders invited to and taking part in the trial. Two other frameworks informed the implementation outcomes of interest:Sekhon(36) for acceptability, and Rogers(37) for sustainability, appropriateness and feasibility; and Moore(35) and Fernandez'(38) work guided exploration of mechanisms of impact and how context affected implementation. The context in which the intervention was implemented will also be assessed. Context is variously defined(39) but here contextual features are conceived of broadly as those influencing the delivery of the intervention and include the engagement of leaders and the organisational setting and culture of the service in which the intervention is implemented.(40)

Important features of the process of implementing MOHMQuit were discussed and agreed with a process evaluation working group of the project's Steering Committee (a key governance committee of the project and constituted of research academics, policy makers, managers and leaders(21)). Subsequently, instruments were developed which encompassed both individual and service level data collection. Decisions were made regarding the specific foci s of the process evaluation, acknowledging that "Process evaluations cannot expect to provide answers to all of the uncertainties of a complex intervention. It is generally better to answer the most important questions well than to try to answer too many questions and do so unsatisfactorily." (35)

With that in mind, a focus on fidelity; adoption/uptake; penetration; reach, sustainability and context was agreed. In part these foci were based on learning from the feasibility and acceptability trial of MOHMQuit.(21) In addition, the short duration of the trial (the time from implementation at the first site to the end of data collection, excluding the wash out period, is 24 months and from the final site, only 8 months) would make sustainment challenging to measure. Sustainability is, however, included in the evaluation. Sustainment is "the continued use of a practice that is the

For peer review only - http://bmjopen.bmj.com/site/about/guidelines.xhtml

target of the implementation, whereas sustainability addresses whether the factors are in place to promote the ongoing use." (41)

The process evaluation has three interrelated objectives; to, at both the individual and site level, assess:

1. To what extent MOHMQuit was implemented - measured quantitatively focusing on the implementation outcomes of adoption, fidelity, penetration, reach and sustainability, and will also involve qualitative measures (interviews with leaders)
2. How changes in behaviour were effected (the mechanisms of impact) – measured quantitatively focusing on the implementation outcomes of acceptability, appropriateness and feasibility, and a more nuanced understanding of this from leaders' perspectives in qualitative interviews
3. The impact of context (moderators) on the implementation of MOHMQuit. A moderator is a factor that will strengthen or lessen the influence of a strategy to implement MOHMQuit.(26) We anticipate a number of moderators will be an important part of the context for MOHMQuit implementation, as well as intervention outcomes, affecting the relationship between the implementation outcomes e.g. reach, and the implementation of MOHMQuit. The moderators measured include:

    a. Leadership
        i. Leaders self-assessment of their leadership for implementation at 3 months using the Implementation Leadership Scale(42)
        ii. Clinicians questionnaires at 6 months which include the Leadership Engagement Scale(38)
    b. Implementation climate
        i. Clinician questionnaires at 6 months which include the Implementation Climate Scale(38)
    c. Service Size
    d. Smoking prevalence amongst pregnant women birthing at that site
    e. Other demands on leaders/service e.g. new SCS policies and training or accreditation

In summary, we speculate that the impact of the context on the implementation outcomes could be as follows:

- Leadership and implementation climate - impacting on all outcomes
- Service size, smoking prevalence and models of care - impacting on adoption, appropriateness, feasibility, penetration and sustainability
- Other demands on leaders - impacting on implementation in terms of adoption, fidelity, penetration and sustainability

see Figure 2 below which summarises our speculation about which of each of the context elements might impact on each of the implementation outcomes e.g. we anticipate leadership will impact on all of the implementation outcomes.

For peer review only - http://bmjopen.bmj.com/site/about/guidelines.xhtml

Figure 2: Speculating which context elements may impact on each of the implementation outcomes

For peer review only

For peer review only - http://bmjopen.bmj.com/site/about/guidelines.xhtml

Recruitment and consent

The Local Health Districts (LHDs – which manage public hospitals and provide healthcare services in a defined geographic area) in NSW with relatively high rates of smoking in pregnancy were approached to participate in the MOHMQuit trial. There are 15 LHDs in total, seven with high smoking rates in pregnancy were invited and five agreed to participate in the trial. Between them they selected nine maternity services (sites) to take part. The senior midwives and lead obstetricians from these five LHDs were partner investigators in a Partnership Grant application subsequently awarded by Australia's National Health and Medical Research Council and so their involvement with the project substantially precedes the implementation trial of MOHMQuit.

Individual service leaders and clinicians in each of the nine sites will be provided with a Participant Information Sheet and those who agree to participate in the research will be asked to sign a written consent form indicating their consent to take part in data collection. This consent applies to data collection to measure the implementation outcomes and context as well as the intervention outcomes.

Process evaluation data collection

The process evaluation will adopt a mixed methods approach, collecting quantitative evidence from questionnaires and qualitative evidence of leaders' perceptions of how MOHMQuit may have changed behaviour (where it was perceived to have done so) from semi-structured interviews. Data will be collected by the research team independently from each of the nine MOHMQuit sites. Study-specific questionnaires will be used to collect implementation outcome data from leaders and clinicians at each site at various time points: immediately following training, three months after the training and six months after the training as outlined below. To minimise participant burden, the questionnaires will also collect the data required to measure the intervention outcomes.

Qualitative data will be collected using semi-structured interviews with leaders six months after the training at each site. The interviews have three key purposes. Firstly, interviews will collect data on the components of MOHMQuit which have been implemented in the six months following the MOHMQuit training (uptake) e.g. use of the report template for the electronic medical record system facilitating leaders' scrutiny of their services' SCS performance for feedback and continuous improvement, or MOHMQuit training delivered by the service themselves using the train the trainer manual. Secondly, they will collect data to support the calculation of an implementation cost as part of the detailed economic evaluation of MOHMQuit, the subject of a separate paper, (28) by recording how much time leaders' assess they spend implementing those components of MOHMQuit. Finally, they will collect data which will enhance the contextual information collected by the research team by eliciting leaders' perspectives of the enablers and barriers of the implementation of MOHMQuit and what might be improved with regard to it. Interviews will be conducted using the Teams platform, recorded and transcribed. They will be guided by an interview schedule driven by the implementation outcomes and the contextual factors that supported or hindered implementation and any adaptations made to the intervention. The semi-structured nature of the interviews will allow for flexibility in questioning and expansion on responses.

Data collection from leaders and antenatal care clinicians will be as follows:

Leaders

- An online questionnaire to <u>all</u> leaders **three months after** the training at each site regardless of whether they attended MOHMQuit training (anticipated numbers of leaders who will be invited approximately 55) .

For peer review only - http://bmjopen.bmj.com/site/about/guidelines.xhtml

- A semi-structured one to one telephone interview **six months after** the training with the midwifery partner investigator and one to two other leaders at each site.

Antenatal care clinicians

- A paper questionnaire **immediately following the training** at each site to participants who attended training (anticipated numbers of participants who will be invited approximately 250).
- An online questionnaire to all antenatal care clinicians and AHWs **six months after** the training at each site regardless of whether they attended MOHMQuit training (anticipated numbers of participants who will be invited approximately 300).

In addition, attendance and fidelity information (which aspects of the training were delivered) will be kept by the research team during each training event and the attendance and engagement at various meetings that are components of MOHMQuit. The additional data collection includes:

- Training logs – to calculate proportion attended at each training event (attendance/invited)
- A 'fidelity checklist' of which elements of the training were covered during each training event
- Attendance and notes from 10 week warm-up meetings
- Attendance and notes from 2 week warm-up meetings
- Attendance and notes from 2 week follow-up meetings
- Attendance and notes from 4 week follow-up meetings
- Attendance and notes from monthly Community of Practice meetings

For each site a 'context table' will be completed by the research team using publicly available sources and with input from partner investigators at each site (Table 1).

Table 1: Key contextual information collected for each site

**Number of births at site 2020**
**Smoking prevalence 2020**
**Performance against the NSW Ministry of Health's performance indicator of antenatal smoking**
**Safer Baby Bundle at site?* (Yes/No)**
**Preparation and training for new NSW Maternity Care Policy (RSVP)# overlaps with MOHMQuit timing? (Yes/No)**
**Other SCS initiatives running at the site? (Yes/No)**
**Accreditation for Quality Improvement going on concurrent with MOHMQuit? (Yes/No)**
**Leadership structure at the site**
**Models of care offered and proportion of women at booking and at birth for each model**
**Other e.g. external events like disasters, vacant posts**

* Safer Baby Bundle is a multi-component intervention in maternity service which aims to reduce the number of preventable stillbirths

# The RSVP policy is a policy directive establishing minimum requirements for health services to provide evidence-based smoking cessation support to women before during and after pregnancy. The RSVP Policy was released 14 October 2022.

We anticipate that the data collection itself may have the beneficial sustainability effect of reminding leaders and clinicians about MOHMQuit and possibly prompting renewed attention and/or commitment to it.

For peer review only - http://bmjopen.bmj.com/site/about/guidelines.xhtml

Table 2 provides detail of working definitions and how each of the implementation outcomes and contextual features will be measured at which timepoints, using which instruments with whom, and which strategies (components of the MOHMQuit intervention) are aimed to maximise the implementation outcomes. Further detail is available in Supplementary Table 1.

For peer review only

For peer review only - http://bmjopen.bmj.com/site/about/guidelines.xhtml

Table 2: Implementation outcomes, definitions, strategies for maximising implementation outcomes, frameworks used and measurement items

Implementation outcome	Strategies used to maximise implementation outcomes	Frameworks used	Data collection instruments, timing, participants
Adoption/Uptake (intention or action to try to employ MOHMQuit)	• Warm-up and follow-up meetings<br>• Community of Practice	Proctor(29)<br>RE-AIM(31) (Adoption)	• Attendance at warm-up and follow up meetings<br>• 3 months post-training - questionnaire with leaders<br>• 6 months post-training - questionnaire with clinicians<br>• 6 months post- training - interview with leaders<br>• Community of Practice peer support meetings attendance
Fidelity (delivered as intended in the Protocol(23), adherence)	• Warm-up and follow-up meetings<br>• Consistency in the team delivering MOHMQuit training at each site in the first instance<br>• Clear plans and materials for content of training	Proctor(29)<br>RE-AIM(31) (Implementation)	• Attendance at warm-up and follow up meetings<br>• Training logs of expected and actual attendance at training of leaders and clinicians<br>• Fidelity record (which aspects of the planned training were actually delivered)<br>• 6 months post- training - interview with leaders
Penetration (degree of integration of MOHMQuit practices within the service)	• Involving leaders in the training for clinicians for a whole-of-service approach<br>• MOHMQuit leadership components include repeated audit and feedback plus action planning; developing and implementing a clinical pathway for SCS; and the development and maintenance of SCS 'champions' within each service<br>• Train the trainer model an integral part of the intervention	Proctor(29)<br>RE-AIM(31) (Adoption)	• 3 months post-training - questionnaire with leaders<br>• 6 months post-training - interview with leaders
Reach (did MOHMQuit include everyone that it aimed to?)	• 10-week warm-up meetings to allow time for planning and rostering<br>• The train the trainer model as an integral part of the intervention to support participation of all relevant existing and new staff	RE-AIM(35)	• Training logs of expected and actual attendance at training of leaders and clinicians recorded at the time of training<br>• 3 months post-training - questionnaire with leaders<br>• 6 months post-training - interview with leaders
Sustainability (factors promoting ongoing use of MOHMQuit)	• MOHMQuit leadership components include repeated audit and feedback plus action planning; developing and implementing a clinical pathway for SCS; and development and maintenance of SCS 'champions' within each service<br>• Train the trainer model an integral part of the intervention<br>• The Community of Practice	Proctor(29)<br>RE-AIM(31) (Maintenance)<br>Rogers(37)	• 6 months post-training - questionnaire with clinicians<br>• 6 months post-training - interview with leaders<br>• Community of Practice peer support attendance data

For peer review only - http://bmjopen.bmj.com/site/about/guidelines.xhtml

Implementation outcome	Strategies used to maximise implementation outcomes	Frameworks used	Data collection instruments, timing, participants
Acceptability (how palatable is MOHMQuit to clinicians and leaders?)	• Comprehensive systematic design of MOHMQuit using the Behaviour Change Wheel with input from clinicians and leaders(21 22)<br>• Feasibility and acceptability trial with subsequent minor amendments to the intervention(21)<br>• 10 week warm-up includes the history of MOHMQuit so leaders are reassured about its quality, relevance and acceptability	Proctor(29)<br>Sekhon(36)	• Immediately post-training - questionnaire with clinicians<br>• 3 months post-training - questionnaire with leaders<br>• 6 months post-training - questionnaire with clinicians<br>• 6 months post- training - interview with leaders
Appropriateness (perceived fit or relevance of MOHMQuit with the service)	• Comprehensive and systematic design of MOHMQuit using the Behaviour Change Wheel integrating input from clinicians and leaders(21 22)<br>• Feasibility and acceptability trial with subsequent minor amendments to the intervention(21)<br>• 10 week warm-up includes the history of MOHMQuit so leaders are reassured about its quality, relevance and acceptability	Proctor(29)<br>Rogers(37)	• 6 months post-training - interview with leaders
Feasibility (actual fit – the extent to which MOHMQuit can be integrated into usual care in a service)	• Comprehensive and systematic design of MOHMQuit using the Behaviour Change Wheel integrating input from clinicians and leaders(21 22)<br>• Feasibility and acceptability trial with subsequent minor amendments to the intervention(21)<br>• 10 week warm-up includes the history of MOHMQuit so leaders are reassured about its quality, relevance and acceptability	Proctor(29)<br>Rogers(37)	• 3 months post-training - questionnaire with leaders<br>• 6 months post-training - interview with leaders
HOW behaviour was changed		Moore(35)	• 6 months post-training - interview with leaders
**HOW context affected implementation**	• **Commitment of maternity service leaders in the research as Partner Investigators and members of MOHMQuit Steering Committee and various working groups**<br>• **Warm-up meetings and follow up meetings**<br>• **Community of Practice**	**Fernandez(38)**	• **Key contextual information (Table 1) completed by research team during the implementation**<br>• **3 months post-training - questionnaire with leaders**<br>• **6 months post-training - questionnaire with clinicians**<br>• **6 months post-training - interview with leaders**

BOLD TYPEFACE INDICATES OUTCOMES THAT WILL BE THE FOCUS OF THE PROCESS EVALUATION

Implementation cost is not included in Table 2 as a detailed economic evaluation of MOHMQuit is taking place and is the subject of a separate paper.(28) Data to contribute to the economic evaluation will be collected as part of the semi-structured interview with leaders.

For peer review only - http://bmjopen.bmj.com/site/about/guidelines.xhtml

Patient and public involvement

As this is an implementation science trial, our partners in identifying the need for the study and in its design and implementation were health service clinicians, leaders and policy makers.(21) Patients were not involved in designing or implementing the research, but are participants in the trial(23) but not in the process evaluation.

Data analysis

We will assess each of the implementation outcomes (Table 2) for each site, including assessing variation across the nine sites. At this stage it is not possible to definitively describe which of the implementation outcomes our analyses will be focused on as that will depend on the variation in implementation outcomes across sites. For example, if there is little variation in fidelity it will not help explain the MOHMQuit (intervention) outcomes. However, where appropriate descriptive statistics (measures of central tendency, standard deviations and proportions) will be produced using data from questionnaire responses from clinicians and leaders to summarise quantitative results by participant and by site.

Analyses for the moderators will include calculation of a measure of central tendency, for the leadership(42) sub-scales for each participant. There are four subscales: the proactive subscale, the knowledgeable subscale, the supportive subscale, and the perseverant subscale. A measure of central tendency for each set of items that load onto the relevant subscale will be calculated for each subscale. A measure of central tendency of the scale scores will be calculated which will provide a total score for the Implementation Leadership Scale.(42) In addition, scores will be aggregated to provide a site-level score. We do not anticipate adding these results, or any of the data from Table 1 to any model but they will help constitute a broader assessment of the context for implementation to contribute to understanding of in which sites, and how, MOHMQuit was effective.

Qualitative data from semi-structured interviews with leaders will be analysed descriptively to explore perspectives of uptake by site and thematically across all sites regarding the enablers, barriers and how implementation of MOHMQuit might be improved.(43)

Data from multiple sources will facilitate triangulation, for example collecting data about acceptability from quantitative data (post-training questionnaires from clinicians and questionnaires at six months from all clinicians) along with qualitative interviews with leaders from each site. This mixed methods approach will broaden and deepen understanding of the results of the trial. The key findings will be presented in an integrated way using a side-by-side joint display table(44) each source being given equal weight.

Figure 3 below describes this visually.

For peer review only - http://bmjopen.bmj.com/site/about/guidelines.xhtml

Figure 3: Mixed methods approach to data collection and analysis

For peer review only

For peer review only - http://bmjopen.bmj.com/site/about/guidelines.xhtml

Ethical considerations and dissemination

The process evaluation received ethical approval from the NSW Population Health Services Research Ethics Committee (Reference Number 2021/ETH00887) on July 23rd, 2021. Results of the process evaluation will be written up for publication in peer-reviewed journals and presented at conferences. The process evaluation will perform a formative function facilitated by the stepped-wedge design with sites receiving the intervention in a staggered implementation, allowing for further polishing of the intervention as the trial proceeds. The process evaluation will also provide contextual information to elucidate the findings of the trial in terms of how MOHMQuit may have been effective in some sites but not in others. This understanding is critical in relation to rolling out MOHMQuit across NSW should the intervention prove to be effective.

Trial registration number

The MOHMQuit trial is registered with ANZCTR (www.anzctr.org.au): ACTRN12622000167763.

DISCUSSION

Implementation science is the study of approaches that support the systematic uptake of research findings into 'usual care'.(45) In cases where there is an urgent need for behaviour change and a clear evidence to practice gap, such as with SCS in antenatal care, implementation science provides a framework for examining an intervention such as MOHMQuit. This paper describes the mixed-methods design and underpinning frameworks for the process evaluation of MOHMQuit as part of an implementation science study. MOHMQuit is a complex multi-component intervention designed using the Behaviour Change Wheel.(20) It aims to change the behaviour of antenatal care providers to improve the support provided to women to stop smoking in pregnancy. MOHMQuit is being implemented in a stepped-wedge effectiveness trial across nine publicly funded maternity services in NSW.(23)

The process evaluation will facilitate the ongoing refinement of MOHMQuit and will provide an assessment of the extent to which MOHMQuit was implemented, what the mechanisms of impact were and what the context of implementation was, and how it affected the implementation of MOHMQuit. It will also inform other components of the study for example contributing data to support costing of MOHMQuit for the economic evaluation. We anticipate that the findings from the process evaluation will contextualise and aid understanding of our trial results, and may support the further implementation of MOHMQuit in NSW. For example, if it transpires that implementation leadership is more evident in those sites where MOHMQuit was shown to be particularly effective, the scale-up would need to include a focus on *implementation leadership* and on implementing the leadership components of the intervention. Our process evaluation will also contribute knowledge about the implementation of stepped-wedge trials which may be useful to others in the future. Whilst we have described our intended approach to evaluating the implementation of MOHMQuit, we have also included flexibility of approach in recognition of unanticipated implementation factors that may surface.(40)

Smoking in pregnancy is an ongoing public health challenge and represents a considerable gap between the evidence for smoking cessation support and practice. Providing a broader understanding of how MOHMQuit was or was not effective will be key to its potential future roll-out/scale up.

For peer review only - http://bmjopen.bmj.com/site/about/guidelines.xhtml

Empirical testing of the theory

Implementation science is a relatively new academic endeavour and this process evaluation has the potential to contribute to a growing body of evidence of approaches to implementing comprehensive stepped-wedge trial designs that are inclusive of process evaluation.

Strengths and limitations

The process evaluation has been designed using implementation science frameworks and explores the implementation of MOHMQuit, a thorough and theoretically underpinned intervention and trial design.(21) The results of the trial will provide further evidence for the effectiveness, or otherwise, of this theoretically driven approach. The mixed methods approach in the process evaluation includes qualitative and quantitative data collection from a wide range of leaders and clinicians in each MOHMQuit site, some of whom will not have directly participated in the MOHMQuit training, as well as publicly-available data and observational data from the research team implementing MOHMQuit. This approach has the potential to produce findings that have depth and nuance and will aid understanding of the trial findings. However, MOHMQuit is a complex intervention with many moving parts which interact with one another, and the stakeholders involved. No process evaluation is able to collect data to understand all aspects of these interactions. In addition, the MOHMQuit trial is a 'real world' trial. This has strengths in producing findings that can be confidently understood as realistic, however it also produces many challenges including the potential impact of new policies and procedures, staffing issues etc. many of which we have aimed to record as part of the process evaluation but some of which we are likely to have missed. This may compromise our capacity to fully understand and accurately interpret the intervention outcomes.

Trial status

Recruitment for the trial is underway. Process evaluation data collection commenced in March 2023 and will conclude in May 2024.

For peer review only - http://bmjopen.bmj.com/site/about/guidelines.xhtml

ACKNOWLEDGEMENTS

This paper is submitted on behalf of the MOHMQuit Trial team, including all chief investigators, partner investigators and associate investigators, and co-researchers and site leads at each of the MOHMQuit sites. In addition to the named authors, the team includes Dheya Al Mashat (NSW Health), Dianne Avery (NSW Health), Elizabeth Best (NSW Ministry of Health), Alecia Brooks (Cancer Council NSW), Rashna Chinoy (NSW Health), Justine Elliot (NSW Health), Jacinta Felsch (NSW Health), Mohamed Foda (NSW Health), Sandra Forde (NSW Health), Tara Farrugia (NSW Health), Tracey Greenberg (Alcohol and Drug Service, St Vincent's Hospital Sydney), Jane Griffith (NSW Health), Madeline Hubbard (NSW Health), Damien McCaul (NSW Ministry of Health), James McLennan (Alcohol and Drug Service, St Vincent's Hospital Sydney), Kate Reakes (Cancer Institute NSW), Virginia Stulz (NSW Health and Western Sydney University), Tracey Zakazakaarcher (NSW Health), and Lou Atkins (University College London) who provided excellent early guidance on the process evaluation design.

AUTHORS' CONTRIBUTIONS

The process evaluation was conceived and designed by all authors: MP, JL, CP, LT, LB, CA, BB, AC and AM . The first draft of the paper was written by JL with input from MP and CP before receiving input from all other authors. All authors have read and approved the final manuscript.

FUNDING

This work is currently supported by a partnership grant from the NHMRC (GNT1185261). Previously, NHMRC funding (GNT1072213) and the Cancer Institute NSW funding (13/ECF/1-11) supported the developmental work of MOHMQuit. MP was also supported during this time by a fellowship grant from the NHMRC (GNT1159601). The funding bodies did not have any role in the design of the study and collection, analysis and interpretation of data.

Ethics approval and consent to participate

Ethics approval for the research was received from the Population Health Services Research Ethics Committee (Reference Number 2021/ETH00887), on July 23rd, 2021. All potential participants will be provided with a Participant Information Sheet (PIS). A signed (written) consent form will be obtained by site trial staff for all maternity service leaders and clinicians who participate in the trial.

Clinicians: Participation of clinicians (anonymous survey participation) is voluntary. Participant Information Sheets for clinicians will explicitly state that the decision to participate or not participate will not influence their professional standing or the care of any of their patients/clients in any way.

Maternity service leaders: The participation of maternity service leaders (semi-structured interviews) is voluntary. The Participant Information Sheet for leaders will explicitly state that the decision to participate or not participate will not influence their professional standing or the care of any of their patients/clients in any way. The Participant Information Sheet will also detail that information shared in interviews will be de-identified before publication or dissemination.

Availability of data and materials

Not applicable

COMPETING INTERESTS

None declared.

For peer review only

For peer review only

References

1. Australian Institue of Health and Welfare. Australia's mothers and babies 2020, web report - Summary Canberra: Australian Government; 2022 [Available from: https://www.aihw.gov.au/reports/mothers-babies/australias-mothers-babies/contents/antenatal-period/smoking-during-pregnancy accessed 23 February 2023.
2. Diamanti A, Papadakis S, Schoretsaniti S, et al. Smoking cessation in pregnancy: An update for maternity care practitioners. *Tobacco induced diseases* 2019;17:57. doi: https://doi.org/10.18332/tid/109906
3. Lange S, Probst C, Rehm J, et al. National, regional, and global prevalence of smoking during pregnancy in the general population: a systematic review and meta-analysis. *The Lancet Global Health* 2018;6(7):e769-e76. doi: https://doi.org/10.1016/S2214-109X(18)30223-7
4. Avşar TS, McLeod H, Jackson L. Health outcomes of smoking during pregnancy and the postpartum period: an umbrella review. *BMC Pregnancy and Childbirth* 2021;21(1):254. doi: 10.1186/s12884-021-03729-1
5. US Department of Health and Human Services. The Health Consequences of Smoking: 50 Years of Progress. A Report of the Surgeon General. Atlanta, GA: U.S. Department of Health and Human Services, Centers for Disease Control and Prevention, National Center for Chronic Disease Prevention and Health Promotion, Office on Smoking and Health, 2014.
6. Greenhalgh E, Ford C, Winstanley M. Section 3.7 Pregnancy and smoking Melbourne, Victoria: Cancer Council; 2021 [Available from: https://www.tobaccoinaustralia.org.au/chapter-3-health-effects/3-7-pregnancy-and-smoking accessed January 2024.
7. U.S. Department of Health & Human Services. Smoking Cessation: A Report of the Surgeon General. Rockville: US Department of Health and Human Services, 2020.
8. NSW Ministry of Health. The First 2000 Days Framework. In: Health and Social Policy Branch, ed. Sydney, Australia, 2019.
9. NSW Ministry of Health. First 2000 Days Implementation Strategy 2020-2025. St Leonards: NSW Ministry of Health, 2021.
10. NSW Ministry of Health. NSW Clinical Guidelines for the Management of Substance Use During Pregnancy, Birth and the Postnatal Period. Sydney: NSW Ministry of Health, 2014.
11. Passey ME, Longman JM, Adams CM, et al. Factors associated with provision of smoking cessation support to pregnant women – a cross-sectional survey of midwives in New South Wales, Australia. *BMC Pregnancy and Childbirth* 2020;20:219.
12. Longman JM, Adams CM, Johnston JJ, et al. Improving implementation of the smoking cessation guidelines with pregnant women: How to support clinicians? *Midwifery* 2018;58:137-44. doi: https://doi.org/10.1016/j.midw.2017.12.016
13. Passey M, Sanson-Fisher RW. Provision of antenatal smoking cessation support: a survey with pregnant Aboriginal and Torres Strait Islander women. *Nicotine Tob Res* 2015;17(6):746-49.
14. Bar Zeev Y, Bonevski B, Twyman L, et al. Opportunities Missed: A Cross-sectional Survey of the Provision of Smoking Cessation Care to Pregnant Women by Australian General Practitioners and Obstetricians. *Nicotine & tobacco research: official journal of the Society for Research on Nicotine and Tobacco* 2017;19(5):636-41.
15. Hoekzema L, Werumeus Buning A, Bonevski B, et al. Smoking rates and smoking cessation preferences of pregnant women attending antenatal clinics of two large Australian maternity hospitals. *Aust N Z J Obstet Gynaecol* 2014;54(1):53-8.
16. Passey ME, Sanson-Fisher RW, Stirling JM. Supporting pregnant Aboriginal and Torres Strait Islander women to quit smoking: views of antenatal care providers and pregnant Indigenous women. *Matern Child Health J* 2014;18:2293-99.
17. Chamberlain C, O'Mara-Eves A, Porter J, et al. Psychosocial interventions for supporting women to stop smoking in pregnancy. *The Cochrane Library* 2017(2) doi: 10.1002/14651858.CD001055.pub5.

For peer review only

18. Skivington K, Matthews L, Simpson SA, et al. A new framework for developing and evaluating complex interventions: update of Medical Research Council guidance. *bmj* 2021;374
19. U.S. Department of Health and Human Services. Smoking Cessation. A Report of the Surgeon General. Atlanta GA: U.S. Department of Health and Human Services, Centers for Disease Control and Prevention, National Center for Chronic Disease Prevention and Health Promotion, Office on Smoking and Health, 2020.
20. Michie S, van Stralen MM, West R. The behaviour change wheel: A new method for characterising and designing behaviour change interventions. *Implement Sci* 2011;6:42.
21. Passey ME, Adams C, Paul C, et al. Improving implementation of smoking cessation guidelines in pregnancy care: Development of an intervention to address system, maternity service leader and clinician factors. *Implementation Science Communications* 2021;2:128. doi: https://doi.org/10.1186/s43058-021-00235-5
22. Longman J, Adams C, Paul C, et al. Improving clinicians' implementation of guidelines to help women stop smoking in pregnancy: developing evidence-based print and video materials. *Int J Environ Res Public Health* 2021;18(19):10522. doi: https://doi.org/10.3390/ijerph181910522
23. Barnes LAJ, Longman J, Adams C, et al. The MOHMQuit (Midwives and Obstetricians Helping Mothers to Quit Smoking) Trial: protocol for a stepped-wedge implementation trial to improve best practice smoking cessation support in public antenatal care services. *Implement Sci* 2022;17(1):79. doi: 10.1186/s13012-022-01250-3
24. Bar-Zeev Y, Bonevski B, Lim LL, et al. Improving health providers smoking cessation care in pregnancy: A systematic review and meta-analysis. *Addict Behav* 2019;93:29-38. doi: https://doi.org/10.1016/j.addbeh.2019.01.002
25. Lewis CC, Boyd MR, Walsh-Bailey C, et al. A systematic review of empirical studies examining mechanisms of implementation in health. *Implement Sci* 2020;15:1-25.
26. Lewis CC, Klasnja P, Powell BJ, et al. From classification to causality: advancing understanding of mechanisms of change in implementation science. *Frontiers in public health* 2018;6:136.
27. Springer MV, Sales AE, Islam N, et al. A step toward understanding the mechanism of action of audit and feedback: a qualitative study of implementation strategies. *Implement Sci* 2021;16:1-13.
28. Pearce A, Scarfe J, Jones M, et al. Study protocol of an economic evaluation embedded in the Midwives and Obstetricians Helping Mothers to Quit Smoking (MOHMQuit) trial. *BMC Health Serv Res* 2023;23(1):1-9.
29. Proctor E, Silmere H, Raghavan R, et al. Outcomes for implementation research: conceptual distinctions, measurement challenges, and research agenda. *Administration and policy in mental health and mental health services research* 2011;38(2):65-76.
30. Graham Moore SA, Barker M, Bond L, et al. Process evaluation of complex interventions. *Complex Interventions in Health: An overview of research methods* 2015:222.
31. Glasgow RE, Vogt TM, Boles SM. Evaluating the public health impact of health promotion interventions: the RE-AIM framework. *Am J Public Health* 1999;89(9):1322-27.
32. Global Alliance for Chronic Diseases. GACD Process Evaluation Guidelines, 2015 [Available from: https://www.gacd.org/perch/resources/gacd-pe-guidelines-v1-0.pdf.
33. Hawe P, Shiell A, Riley T. Complex interventions: how "out of control" can a randomised controlled trial be? *Bmj* 2004;328(7455):1561-63.
34. Kirk MA, Moore JE, Wiltsey Stirman S, et al. Towards a comprehensive model for understanding adaptations' impact: the model for adaptation design and impact (MADI). *Implement Sci* 2020;15:1-15.
35. Moore GF, Audrey S, Barker M, et al. Process evaluation of complex interventions: Medical Research Council guidance. *bmj* 2015;350
36. Sekhon M, Cartwright M, Francis JJ. Acceptability of healthcare interventions: an overview of reviews and development of a theoretical framework. *BMC Health Serv Res* 2017;17(1):88.

For peer review only

37. Rogers EM. Diffusion of Innovations. 4th ed. New York: The Free Press 1995.
38. Fernandez ME, Walker TJ, Weiner BJ, et al. Developing measures to assess constructs from the Inner Setting domain of the Consolidated Framework for Implementation Research. *Implement Sci* 2018;13(1):52. doi: 10.1186/s13012-018-0736-7
39. Craig P, Di Ruggiero E, Frolich KL, et al. Taking account of context in population health intervention research: guidance for producers, users and funders of research. . Canadian Institutes of Health Research (CIHR)–National Institute for Health Research (NIHR) Southampton: National Institute for Health Research, 2018.
40. Beames JR, Lingam R, Boydell K, et al. Protocol for the process evaluation of a complex intervention delivered in schools to prevent adolescent depression: the Future Proofing Study. *BMJ open* 2021;11(1):e042133.
41. Moullin JC, Sklar M, Ehrhart MG, et al. Provider REport of Sustainment Scale (PRESS): development and validation of a brief measure of inner context sustainment. *Implement Sci* 2021;16(1):1-10.
42. Aarons GA, Ehrhart MG, Farahnak LR. The Implementation Leadership Scale (ILS): Development of a Brief Measure of Unit Level Implementation Leadership. *Implement Sci* 2014;9:45. doi: 10.1186/1748-5908-9-45
43. Braun V, Clarke V. Using thematic analysis in psychology. *Qualitative research in psychology* 2006;3(2):77-101.
44. Guetterman TC, Fetters MD, Creswell JW. Integrating quantitative and qualitative results in health science mixed methods research through joint displays. *The Annals of Family Medicine* 2015;13(6):554-61.
45. Eccles M, Mittman B. Welcome to implementation science [Internet]. Vol. 1. *Implementation Science BioMed Central* 2006:1.

For peer review only

**Antenatal Care clinicians**	• Antenatal care midwives<br>• Aboriginal Health Workers (AHWs) - primary healthcare workers who ensure culturally safe maternity care in supporting Aboriginal and/or Torres Strait Islander women or women having an Aboriginal baby<br>• Obstetricians (staff specialists; Visiting Medical Officers with specialist obstetric training, Career Medical Officers) and obstetric registrars
**Leaders**	Maternity service leaders (those who support or supervise health professionals providing antenatal care), including:<br>• Clinical Midwifery Consultants<br>• Maternity Unit Managers<br>• Clinical Midwifery Educators<br>• Clinical Midwifery Specialists<br>• Antenatal clinic coordinators<br>• Obstetric leads

For peer review only

	Implementation outcomes							
**Context measures**	Acceptability	Adoption	Appropriateness	Feasibility	Fidelity	Penetration	Sustainability	Reach
Leadership								
Implementation climate								
Service size								
Smoking prevalence								
Models of care								
Other demands on leaders								

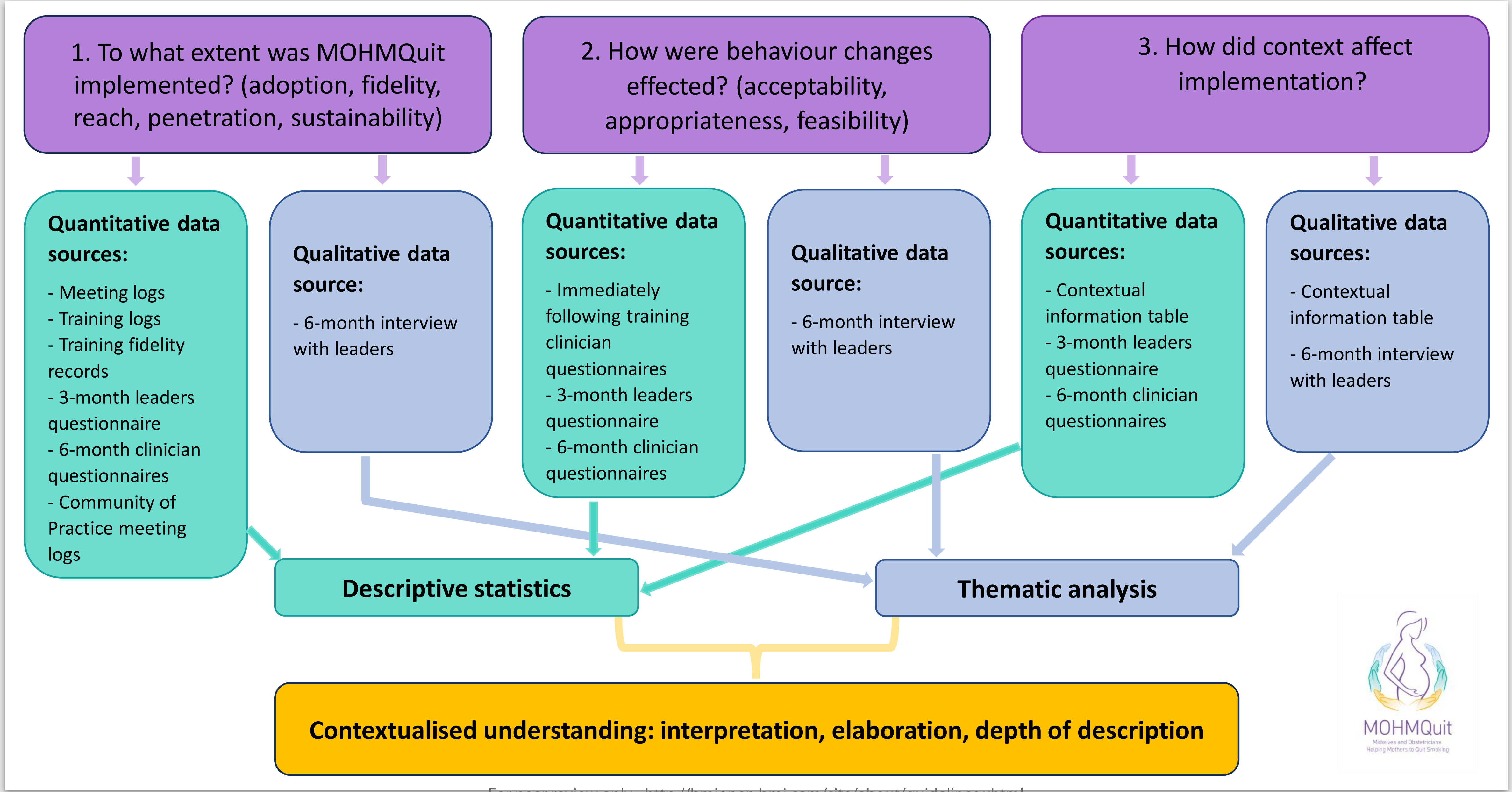
1. To what extent was MOHMQuit implemented? (adoption, fidelity, reach, penetration, sustainability)
2. How were behaviour changes effected? (acceptability, appropriateness, feasibility)
3. How did context affect implementation?
Quantitative data sources:
- Meeting logs
- Training logs
- Training fidelity records
- 3-month leaders questionnaire
- 6-month clinician questionnaires
- Community of Practice meeting logs
Qualitative data source:
- 6-month interview with leaders
Quantitative data sources:
- Immediately following training clinician questionnaires
- 3-month leaders questionnaire
- 6-month clinician questionnaires
Qualitative data source:
- 6-month interview with leaders
Quantitative data sources:
- Contextual information table
- 3-month leaders questionnaire
- 6-month clinician questionnaires
Qualitative data sources:
- Contextual information table
- 6-month interview with leaders
Descriptive statistics
Thematic analysis
Contextualised understanding: interpretation, elaboration, depth of description
MOHMQuit

Supplementary Table 1: Implementation outcomes, definitions, strategies for maximising implementation outcomes, frameworks used and measurement items

Implementation outcome (abbreviated definition)	Strategies used to maximise implementation outcomes	Level of analysis	Frameworks used	Data collection instruments, timing, participants	Items from instruments
**Adoption/Uptake (intention or action to try to employ MOHMQuit)**	• Warm-up meetings<br>• Follow up meetings<br>• Community of Practice	Site level<br>Individual clinician level	Proctor[1]<br>RE-AIM[2]<br>(Adoption)	• Warm-up and follow up meetings<br><br>• 3 months following training - questionnaire for leaders<br><br>• 6 months following training - questionnaire with clinicians<br><br>• 6 months following training - semi-structured interview with leaders<br><br>• Community of Practice peer support meetings attendance data	Meetings<br>* Whether the 10 and 2-week warm-up meetings took place/were attended;<br>* Whether the 2 and 4 week post training meetings took place/were attended<br>3 month questionnaire for leaders<br>* *In the last 3 months did you or any other staff in your service…* (followed by a number of MOHMQuit components)*?*<br>6 month questionnaire for clinicians<br>* *How useful were each of the MOHMQuit resources when working with women (scale of 1-3 Very useful to Not at all useful + Not Applicable as a response option)?*<br>6 month interview guide for leaders<br>* *Have you, or any of your colleagues implemented any of the components of MOHMQuit* (followed by a number of MOHMQuit components)*?*<br>* *What do you think helped in delivering MOHMQuit?*<br><br>Community of Practice meetings<br>* Sites attending community of practice meetings
**Fidelity (delivered as intended in the Protocol[3], adherence)**	• Warm-up and follow-up meetings<br>• Consistency in the team delivering MOHMQuit training at each site in the first instance	Site level	Proctor[1]<br>RE-AIM[2]<br>(Implementation)	• Warm-up and follow up meetings<br><br>• Training logs of expected and actual	Meetings<br>* Whether the 10 and 2-week warm-up meetings took place/were attended;<br>* Whether the 2 and 4 week post training meetings took place/were attended<br><br>Training logs

For peer review only

Implementation outcome (abbreviated definition)	Strategies used to maximise implementation outcomes	Level of analysis	Frameworks used	Data collection instruments, timing, participants	Items from instruments
	• clear plans and materials for content of training			attendance at training of leaders and clinicians recorded at the time of training<br>• Fidelity record (a checklist of which aspects of the planned training were actually delivered – completed by researchers observing the training, plus any additional observational data about engagement of participants)<br>• 6 months following training - semi-structured interview with leaders	* Proportion of eligible leaders and clinicians who were invited and who actually attended training<br><br>Fidelity record<br>* the extent to which training was delivered as anticipated<br><br>6 month interview guide for leaders<br>* *Were any adaptations made to MOHMQuit? (What/who/when/why/how?)*
Penetration (degree of integration of MOHMQuit practices within the service)	• Involving leaders in the training for clinicians for a whole-of-service approach<br>• MOHMQuit leadership components which focus on repeated audit and feedback plus action planning;	Site level	Proctor[1]<br>RE-AIM[2]<br>(Adoption)	• 3 months following training - questionnaire for leaders<br>• 6 months following training - semi-structured interview with leaders	3 month questionnaire for leaders<br>* *In the last 3 months did you or any other staff in your service…* (followed by a number of MOHMQuit components)*?*<br>6 month interview guide for leaders<br>* *Have you, or any of your colleagues implemented any of the components of MOHMQuit* (followed by a number of MOHMQuit components)*?*<br>* *How much has MOHMQuit become part of 'usual practice'? (e.g. standard operating procedures, local policies, SCS as a*

Implementation outcome (abbreviated definition)	Strategies used to maximise implementation outcomes	Level of analysis	Frameworks used	Data collection instruments, timing, participants	Items from instruments
	developing and implementing a clinical pathway for SCS; and the development and maintenance of SCS 'champions' within each service<br>• The train the trainer model as an integral part of the intervention to support its ongoing implementation				*standing item on meeting agendas, audit part of usual audit schedule etc.)*
Reach (did MOHMQuit include all clinicians and leaders that it aimed to?)	• 10-week warm-up meetings to allow time for planning and rostering<br>• The train the trainer model as an integral part of the intervention to support participation of all relevant existing and new staff	Site level	RE-AIM[4]	• Training logs of expected and actual attendance at training of leaders and clinicians recorded at the time of training<br>• 3 months following training - questionnaire for leaders<br>• 6 months following training - semi-	<u>Training logs</u><br>* Proportion of eligible leaders and clinicians who were invited and proportion who actually attended training (compare the seniority, and role e.g. midwife, obstetrician of those who participated to those who did not)<br><u>3 month questionnaire for leaders</u><br>* *In the last 3 months did you or any other staff in your service design and run any staff training on SCS?* (the train the trainer model);<br>* *Please tell us more about this training* (space to write a qualitative response)<br><u>6 month interview guide for leaders</u>

For peer review only

Implementation outcome (abbreviated definition)	Strategies used to maximise implementation outcomes	Level of analysis	Frameworks used	Data collection instruments, timing, participants	Items from instruments
				**structured interview with leaders**	*** *Have you, or any of your colleagues implemented any of the components of MOHMQuit* (followed by a number of MOHMQuit components including *Designing and running any staff training*)?**
**Sustainability (whether factors are in place to promote the ongoing use of MOHMQuit)**	**• MOHMQuit leadership components which focus on repeated audit and feedback plus action planning; developing and implementing a clinical pathway for SCS; and the development and maintenance of SCS 'champions' within each service<br>• The train the trainer model as an integral part of the intervention to support its ongoing implementation<br>• The Community of Practice**	**Site level<br>Individual clinician level**	**Proctor[1]<br>RE-AIM[2]<br>(Maintenance)<br>Rogers[5]**	**• 6 months following training - questionnaire with clinicians<br>• 6 months following training - semi-structured interview with leaders<br>• Community of Practice peer support attendance data**	**6 month questionnaire for clinicians<br>* *How useful were each of the MOHMQuit resources when working with women (scale of 1-3 Very useful to Not at all useful + Not Applicable as a response option)?*<br>6 month interview guide for leaders<br>* *How much has MOHMQuit become part of 'usual practice'?*<br>* *What do you think helped in delivering MOHMQuit? (contextual factors)*<br>Community of Practice meetings<br>* Sites attending community of practice meetings**
Acceptability (how palatable is MOHMQuit to	• Comprehensive and systematic design of MOHMQuit using the	Site level<br>Individual level	Proctor[1]<br>Sekhon[8]	• Immediately following training - questionnaire with clinicians	Immediately following training for clinicians:<br>* *On a scale of 1 to 3 (very useful to not at all useful) what's your impression of how useful the MOHMQuit training is going*

For peer review only

Implementation outcome (abbreviated definition)	Strategies used to maximise implementation outcomes	Level of analysis	Frameworks used	Data collection instruments, timing, participants	Items from instruments
clinicians and leaders?)	Behaviour Change Wheel integrating input from clinicians and leaders[6,7]<br>• Feasibility and acceptability trial with subsequent minor amendments to the intervention[6]<br>• At the 10 week warm-up the long 'history' of the development of MOHMQuit is described to ensure leaders are reassured about its quality, relevance and acceptability			• 3 months following training - questionnaire for leaders<br>• 6 months following training - questionnaire with clinicians<br>• 6 months following training - semi-structured interview with leaders	*to be in helping you provide SCS by addressing gaps in your knowledge/skills/confidence?* (perceived effectiveness[8]);<br>* *On a scale of 1-3 (very much* to *not at all) how much do you think MOHMQuit will help you provide SCS* (perceived effectiveness[8]);<br>**Overall how do you feel about MOHMQuit (scale of 1-4)?* (affective attitude[8])<br>3 month questionnaire for leaders:<br>* *Please give MOHMQuit a score of whether you think it has helped your service to routinely provide evidence-based SCS (scale of 1-10 Has not helped at all* to *Has been entirely helpful)* (perceived effectiveness[8]);<br>6 month questionnaire for clinicians:<br>* *On a scale of 1-5 (Strongly agree* to *Strongly disagree) I am confident providing smoking cessation assistance to pregnant women* (self-efficacy[8]);<br>* *On a scale of 1-5 (Strongly agree* to *Strongly disagree) I am confident arranging follow up support for pregnant smokers* (self-efficacy[8]);<br>* *On a scale of 1-4* (very much to not at all) *to what extent did MOHMQuit help you to provide high quality smoking cessation support to women at every visit?* (perceived effectiveness[8])<br>6 month interview guide for leaders<br>* *How would you describe MOHMQuit (what it is and how it aims to improve practice) to a leader in a maternity service in a different hospital?* (intervention coherence[8])<br>* *Did MOHMQuit improve the SCS provided to pregnant women in your service?* (perceived effectiveness[8])?

For peer review only

Implementation outcome (abbreviated definition)	Strategies used to maximise implementation outcomes	Level of analysis	Frameworks used	Data collection instruments, timing, participants	Items from instruments
Appropriateness (perceived fit or relevance of MOHMQuit with the service)	• Comprehensive and systematic design of MOHMQuit using the Behaviour Change Wheel integrating input from clinicians and leaders[6 7]<br>• Feasibility and acceptability trial with subsequent minor amendments to the intervention[6]<br>• At the 10 week warm-up the long 'history' of the development of MOHMQuit is described to ensure leaders are reassured about its quality, relevance and acceptability	Site level	Proctor[1]<br>Rogers[5]	• 6 months following training - semi-structured interview with leaders	6 month interview guide for leaders<br>* *Were any adaptations made to MOHMQuit? (What/who/when/why/how?)*
Feasibility (actual fit – the extent to which MOHMQuit can be integrated into usual care in a service)	• Comprehensive and systematic design of MOHMQuit using the Behaviour Change Wheel integrating input from clinicians and leaders[6 7]	Site level	Proctor[1]<br>Rogers[5]	• 3 months following training - questionnaire for leaders<br>• 6 months following training - semi-structured interview with leaders	3 month questionnaire for leaders<br>* *In the last 3 months did you or any other staff in your service…* (followed by a number of MOHMQuit components)*?*<br>6 month interview guide for leaders<br>* *Have you, or any of your colleagues implemented any of the components of MOHMQuit* (followed by a number of MOHMQuit components)*?*

Implementation outcome (abbreviated definition)	Strategies used to maximise implementation outcomes	Level of analysis	Frameworks used	Data collection instruments, timing, participants	Items from instruments
	• Feasibility and acceptability trial with subsequent minor amendments to the intervention[6]<br>• At the 10 week warm-up the long 'history' of the development of MOHMQuit is described to ensure leaders are reassured about its quality, relevance and acceptability				** How much has MOHMQuit become part of 'usual practice'? (e.g. standard operating procedures, local policies, SCS as a standing item on meeting agendas, audit part of usual audit schedule etc.)*
HOW the implementation of the intervention changed behaviour – the 'mechanisms of impact'+ acceptability, appropriateness and feasibility above		Site level	Moore[4]	• 6 months following training - semi-structured interview with leaders	<u>6 month interview guide for leaders</u><br>** Did MOHMQuit improve the SCS provided to pregnant women in your service? How did it do this?*<br>** How can the implementation of MOHMQuit be improved?*
**HOW context affected implementation**	• **Commitment of maternity service leaders in the**	**Site level**	**Fernandez[9]**	• **Key contextual information (Table 1) completed by research**	**<u>See Table 1 above</u>**<br>*** Birth numbers; smoking prevalence; Performance against the performance indicator of antenatal smoking; Safer Baby**

Implementation outcome (abbreviated definition)	Strategies used to maximise implementation outcomes	Level of analysis	Frameworks used	Data collection instruments, timing, participants	Items from instruments
	research as Partner Investigators on the grant and as members of the MOHMQuit research Steering Committee and various working groups<br>• Warm-up meetings<br>• Follow up meetings<br>• Community of Practice			team during the implementation<br>• 3 months following training - questionnaire for leaders<br>• 6 months following training - questionnaire with clinicians<br>• 6 months following training - semi-structured interview with leaders	Bundle; RSVP policy; Other SCS initiatives; Accreditation; leadership structure; models of care on offer; other<br><br>3 month questionnaire for leaders<br>* *Please indicate the extent to which you agree (from Not at all* to *Very great extent)…* all 12 items from the Implementation Leadership Scale[10] e.g. *I have developed a plan to facilitate the implementation of MOHMQuit*<br>6 month questionnaire for clinicians<br>* *How well do you feel your service leadership has supported the implementation of MOHMQuit* (scale of 1-5 from *Strongly Disagree* to *Strongly* agree) the 4 items of the Implementation Climate measure[9] *e.g. Our service leadership makes sure that we have the time and space necessary to discuss changes to improve care*<br>* *…the general feeling for implementation of MOHMQuit in your service* (scale of 1-5 from *Strongly Disagree* to *Strongly* agree) the 4 items from the Leadership Engagement measure[9] *e.g. Our service staff get the support they need to implement MOHMQuit*<br>6 month interview guide for leaders<br>* *Has anything changed in terms of your or others' leadership within the service/s around SCS due to MOHMQuit? (Why? How?)*<br>* *What do you think helped in delivering MOHMQuit?*<br>* *What made delivering MOHMQuit more of a challenge?*

BOLD TYPEFACE INDICATES OUTCOMES THAT WILL BE THE FOCUS OF THE PROCESS EVALUATION

Implementation cost is not included in Table 2 as a detailed economic evaluation of MOHMQuit is taking place and is the subject of a separate paper.[11] Data to contribute to the economic evaluation will be collected as part of the semi-structured interview with leaders.

References

1. Proctor E, Silmere H, Raghavan R, et al. Outcomes for implementation research: conceptual distinctions, measurement challenges, and research agenda. *Administration and policy in mental health and mental health services research* 2011;38(2):65-76.
2. Glasgow RE, Vogt TM, Boles SM. Evaluating the public health impact of health promotion interventions: the RE-AIM framework. *Am J Public Health* 1999;89(9):1322-27.
3. Barnes LAJ, Longman J, Adams C, et al. The MOHMQuit (Midwives and Obstetricians Helping Mothers to Quit Smoking) Trial: protocol for a stepped-wedge implementation trial to improve best practice smoking cessation support in public antenatal care services. *Implement Sci* 2022;17(1):79. doi: 10.1186/s13012-022-01250-3
4. Moore GF, Audrey S, Barker M, et al. Process evaluation of complex interventions: Medical Research Council guidance. *bmj* 2015;350
5. Rogers EM. Diffusion of Innovations. 4th ed. New York: The Free Press 1995.
6. Passey ME, Adams C, Paul C, et al. Improving implementation of smoking cessation guidelines in pregnancy care: Development of an intervention to address system, maternity service leader and clinician factors. *Implementation Science Communications* 2021;2:128. doi: https://doi.org/10.1186/s43058-021-00235-5
7. Longman J, Adams C, Paul C, et al. Improving clinicians' implementation of guidelines to help women stop smoking in pregnancy: developing evidence-based print and video materials. *Int J Environ Res Public Health* 2021;18(19):10522. doi: https://doi.org/10.3390/ijerph181910522
8. Sekhon M, Cartwright M, Francis JJ. Acceptability of healthcare interventions: an overview of reviews and development of a theoretical framework. *BMC Health Serv Res* 2017;17(1):88.
9. Fernandez ME, Walker TJ, Weiner BJ, et al. Developing measures to assess constructs from the Inner Setting domain of the Consolidated Framework for Implementation Research. *Implement Sci* 2018;13(1):52. doi: 10.1186/s13012-018-0736-7
10. Aarons GA, Ehrhart MG, Farahnak LR. The Implementation Leadership Scale (ILS): Development of a Brief Measure of Unit Level Implementation Leadership. *Implement Sci* 2014;9:45. doi: 10.1186/1748-5908-9-45
11. Pearce A, Scarfe J, Jones M, et al. Study protocol of an economic evaluation embedded in the Midwives and Obstetricians Helping Mothers to Quit Smoking (MOHMQuit) trial. *BMC Health Serv Res* 2023;23(1):1-9.

For peer review only

SPIRIT

STANDARD PROTOCOL ITEMS: RECOMMENDATIONS FOR INTERVENTIONAL TRIALS

SPIRIT 2013 Checklist: Recommended items to address in a clinical trial protocol and related documents*

Section/item	Item No	Description
**Administrative information**		
Title	1	Descriptive title identifying the study design, population, interventions, and, if applicable, trial acronym<br>***Protocol for the process evaluation of an intervention to improve antenatal smoking cessation support (MOHMQuit) in maternity services in New South Wales, Australia***
Trial registration	2a	Trial identifier and registry name. If not yet registered, name of intended registry<br>***The MOHMQuit trial is registered (ACTRN12622000167763 https://www.australianclinicaltrials.gov.au/anzctr/trial/ACTRN12622000167763)***
	2b	All items from the World Health Organization Trial Registration Data Set<br>**N/A**
Protocol version	3	Date and version identifier<br>**The protocol for the whole MOHMQuit trial (which includes the process evaluation) is a published paper (Barnes, L. A. J., J. Longman, C. Adams, C. Paul, L. Atkins, B. Bonevski, A. Cashmore, L. Twyman, R. Bailie, A. Pearce, D. Barker, A. J. Milat, J. Dorling, M. Nicholl and M. Passey (2022). "The MOHMQuit (Midwives and Obstetricians Helping Mothers to Quit Smoking) Trial: protocol for a stepped-wedge implementation trial to improve best practice smoking cessation support in public antenatal care services." Implementation Science 17(1): 79). This paper itself is a more detailed protocol for the process evaluation.**
Funding	4	Sources and types of financial, material, and other support<br>***This work was supported by funding from the NHMRC (GNT1072213) and the Cancer Institute NSW (13/ECF/1-11). MP was supported by a fellowship from the NHMRC (GNT1159601).***

For peer review only

Roles and responsibilities	5a	Names, affiliations, and roles of protocol contributors<br>***Jo Longman[1], Chris Paul[2], Aaron Cashmore[3,4], Laura Twyman[5], Larisa AJ Barnes[1], Cathy Adams[6], Billie Bonevski[7], Andrew Milat[3,4] and Megan E Passey[1]*** **(affiliations are listed on the title page of the paper).**<br>***The process evaluation was conceived and designed by MP, JL, CP, LT, LB, CA, BB and LA. The first draft of the paper was written by JL with input from MP and CP before receiving input from all other authors. All authors have read and approved the final manuscript.***
	5b	Name and contact information for the trial sponsor<br>N/A
	5c	Role of study sponsor and funders, if any, in study design; collection, management, analysis, and interpretation of data; writing of the report; and the decision to submit the report for publication, including whether they will have ultimate authority over any of these activities<br>**The funding bodies did not have any role in the design of the study and collection, analysis and interpretation of data.**
	5d	Composition, roles, and responsibilities of the coordinating centre, steering committee, endpoint adjudication committee, data management team, and other individuals or groups overseeing the trial, if applicable (see Item 21a for data monitoring committee)<br>N/A
**Introduction**		
Background and rationale	6a	Description of research question and justification for undertaking the trial, including summary of relevant studies (published and unpublished) examining benefits and harms for each intervention<br>**Includes research questions driving the process evaluation and justification for the process evaluation – see paragraph *“Aims of the MOHMQuit process evaluation”*.**
	6b	Explanation for choice of comparators<br>N/A
Objectives	7	Specific objectives or hypotheses<br>**See paragraph *“Overall design and objectives of the process evaluation”*.**

For peer review only - http://bmjopen.bmj.com/site/about/guidelines.xhtml

Trial design	8	Description of trial design including type of trial (eg, parallel group, crossover, factorial, single group), allocation ratio, and framework (eg, superiority, equivalence, noninferiority, exploratory)<br>***MOHMQuit is currently being trialled in a multi-site cluster randomised stepped-wedge effectiveness trial in nine sites in publicly-funded maternity services in NSW, Australia***

Methods: Participants, interventions, and outcomes

Study setting	9	Description of study settings (eg, community clinic, academic hospital) and list of countries where data will be collected. Reference to where list of study sites can be obtained<br>**MOHMQuit is currently being trialled in a multi-site cluster randomised stepped-wedge effectiveness trial in nine sites in publicly-funded maternity services in NSW, Australia**.
Eligibility criteria	10	Inclusion and exclusion criteria for participants. If applicable, eligibility criteria for study centres and individuals who will perform the interventions (eg, surgeons, psychotherapists)<br>**See "Recruitment and Consent" section**
Interventions	11a	Interventions for each group with sufficient detail to allow replication, including how and when they will be administered<br>**The MOHMQuit intervention has been described in detail in a previously published manuscript so is described in brief here. See "The MOHMQuit Intervention" section**
	11b	Criteria for discontinuing or modifying allocated interventions for a given trial participant (eg, drug dose change in response to harms, participant request, or improving/worsening disease)<br>N/A
	11c	Strategies to improve adherence to intervention protocols, and any procedures for monitoring adherence (eg, drug tablet return, laboratory tests)<br>N/A
	11d	Relevant concomitant care and interventions that are permitted or prohibited during the trial<br>N/A

For peer review only - http://bmjopen.bmj.com/site/about/guidelines.xhtml

Outcomes	12	Primary, secondary, and other outcomes, including the specific measurement variable (eg, systolic blood pressure), analysis metric (eg, change from baseline, final value, time to event), method of aggregation (eg, median, proportion), and time point for each outcome. Explanation of the clinical relevance of chosen efficacy and harm outcomes is strongly recommended<br>**Primary and secondary intervention outcomes are described in brief:** ***The primary intervention outcome is smoking cessation, and secondary intervention outcomes include changes to clinicians' knowledge, skills, confidence and behaviour in providing SCS and test the 'mechanisms of action' by which each of the components/strategies effect intervention outcomes and moderators of their impact in this framework-driven approach. Cost-effectiveness will be assessed in an economic evaluation.*** **The implementation outcomes (the process evaluation) are described in detail – see "Overall design and objectives of the process evaluation" section and Table 4**
Participant timeline	13	Time schedule of enrolment, interventions (including any run-ins and washouts), assessments, and visits for participants. A schematic diagram is highly recommended (see Figure)<br>**The timeline for the process evaluation is described in the "Trial status" paragraph**.
Sample size	14	Estimated number of participants needed to achieve study objectives and how it was determined, including clinical and statistical assumptions supporting any sample size calculations<br>N/A
Recruitment	15	Strategies for achieving adequate participant enrolment to reach target sample size<br>N/A

Methods: Assignment of interventions (for controlled trials)

Allocation:

Sequence generation	16a	Method of generating the allocation sequence (eg, computer-generated random numbers), and list of any factors for stratification. To reduce predictability of a random sequence, details of any planned restriction (eg, blocking) should be provided in a separate document that is unavailable to those who enrol participants or assign interventions<br>N/A
Allocation concealment mechanism	16b	Mechanism of implementing the allocation sequence (eg, central telephone; sequentially numbered, opaque, sealed envelopes), describing any steps to conceal the sequence until interventions are assigned<br>N/A

Implementation	16c	Who will generate the allocation sequence, who will enrol participants, and who will assign participants to interventions<br>N/A
Blinding (masking)	17a	Who will be blinded after assignment to interventions (eg, trial participants, care providers, outcome assessors, data analysts), and how<br>N/A
	17b	If blinded, circumstances under which unblinding is permissible, and procedure for revealing a participant's allocated intervention during the trial<br>N/A
**Methods: Data collection, management, and analysis**		
Data collection methods	18a	Plans for assessment and collection of outcome, baseline, and other trial data, including any related processes to promote data quality (eg, duplicate measurements, training of assessors) and a description of study instruments (eg, questionnaires, laboratory tests) along with their reliability and validity, if known. Reference to where data collection forms can be found, if not in the protocol<br>**This is described in the "Process evaluation data collection" section**
	18b	Plans to promote participant retention and complete follow-up, including list of any outcome data to be collected for participants who discontinue or deviate from intervention protocols<br>**Plans to keep MOHMQuit at the forefront of clinicians' and leaders' minds (from whom data will be collected 6 months following the intervention) include those addressing sustainability of the intervention: MOHMQuit leadership components; the 'train the trainer' model; and the Community of Practice (Table 4)**
Data management	19	Plans for data entry, coding, security, and storage, including any related processes to promote data quality (eg, double data entry; range checks for data values). Reference to where details of data management procedures can be found, if not in the protocol<br>**This detail is included in the ethics application for the trial (which includes the process evaluation) and for the sake of brevity are not included in this manuscript.**
Statistical methods	20a	Statistical methods for analysing primary and secondary outcomes. Reference to where other details of the statistical analysis plan can be found, if not in the protocol<br>**This is described in the "Data analysis" section**

	20b	Methods for any additional analyses (eg, subgroup and adjusted analyses)<br>N/A
	20c	Definition of analysis population relating to protocol non-adherence (eg, as randomised analysis), and any statistical methods to handle missing data (eg, multiple imputation)<br>N/A
**Methods: Monitoring**		
Data monitoring	21a	Composition of data monitoring committee (DMC); summary of its role and reporting structure; statement of whether it is independent from the sponsor and competing interests; and reference to where further details about its charter can be found, if not in the protocol. Alternatively, an explanation of why a DMC is not needed<br>N/A
	21b	Description of any interim analyses and stopping guidelines, including who will have access to these interim results and make the final decision to terminate the trial<br>N/A
Harms	22	Plans for collecting, assessing, reporting, and managing solicited and spontaneously reported adverse events and other unintended effects of trial interventions or trial conduct<br>N/A
Auditing	23	Frequency and procedures for auditing trial conduct, if any, and whether the process will be independent from investigators and the sponsor<br>N/A
**Ethics and dissemination**		
Research ethics approval	24	Plans for seeking research ethics committee/institutional review board (REC/IRB) approval<br>***Ethics approval for the research was received from the Population Health Services Research Ethics Committee (Reference Number 2021/ETH00887), on July 23rd, 2021***.
Protocol amendments	25	Plans for communicating important protocol modifications (eg, changes to eligibility criteria, outcomes, analyses) to relevant parties (eg, investigators, REC/IRBs, trial participants, trial registries, journals, regulators)<br>N/A
Consent or assent	26a	Who will obtain informed consent or assent from potential trial participants or authorised surrogates, and how (see Item 32)<br>**This is described in the section "Ethical approval and consent to participate"**

For peer review only - http://bmjopen.bmj.com/site/about/guidelines.xhtml

	26b	Additional consent provisions for collection and use of participant data and biological specimens in ancillary studies, if applicable<br>N/A
Confidentiality	27	How personal information about potential and enrolled participants will be collected, shared, and maintained in order to protect confidentiality before, during, and after the trial<br>**This is described in the section "Ethical approval and consent to participate"**
Declaration of interests	28	Financial and other competing interests for principal investigators for the overall trial and each study site<br>**There is a "Competing Interests" statement and a ICMJE Disclosure Form submitted with the manuscript**
Access to data	29	Statement of who will have access to the final trial dataset, and disclosure of contractual agreements that limit such access for investigators<br>N/A
Ancillary and post-trial care	30	Provisions, if any, for ancillary and post-trial care, and for compensation to those who suffer harm from trial participation<br>N/A
Dissemination policy	31a	Plans for investigators and sponsor to communicate trial results to participants, healthcare professionals, the public, and other relevant groups (eg, via publication, reporting in results databases, or other data sharing arrangements), including any publication restrictions<br>**This is described in the "Ethics and dissemination" section**
	31b	Authorship eligibility guidelines and any intended use of professional writers<br>**This level of detail has not been included for the sake of brevity. Professional writers will not be used.**
	31c	Plans, if any, for granting public access to the full protocol, participant-level dataset, and statistical code<br>N/A
**Appendices**		
Informed consent materials	32	Model consent form and other related documentation given to participants and authorised surrogates<br>**This level of detail has not been included for the sake of brevity.**
Biological specimens	33	Plans for collection, laboratory evaluation, and storage of biological specimens for genetic or molecular analysis in the current trial and for future use in ancillary studies, if applicable<br>N/A

*It is strongly recommended that this checklist be read in conjunction with the SPIRIT 2013 Explanation & Elaboration for important clarification on the items. Amendments to the

For peer review only - http://bmjopen.bmj.com/site/about/guidelines.xhtml

protocol should be tracked and dated. The SPIRIT checklist is copyrighted by the SPIRIT Group under the Creative Commons "Attribution-NonCommercial-NoDerivs 3.0 Unported" license.

For peer review only

BMJ Open

Protocol for the process evaluation of an intervention to improve antenatal smoking cessation support (MOHMQuit) in maternity services in New South Wales, Australia

Journal:	*BMJ Open*
Manuscript ID	bmjopen-2023-081208.R2
Article Type:	Protocol
Date Submitted by the Author:	28-Feb-2024
Complete List of Authors:	Longman, Jo; The University of Sydney, University Centre for Rural Health<br>Paul, Christine; University of Newcastle, School of Medicine and Public Health<br>Cashmore, Aaron; NSW Health, Centre for Epidemiology and Evidence; The University of Sydney, School of Public Health, Faculty of Medicine and Health<br>Twyman, Laura; Cancer Council NSW; The University of Newcastle, School of Medicine and Public Health<br>Barnes, Larisa; The University of Sydney, University Centre for Rural Health<br>Adams, Catherine; NSW Health, Clinical Excellence Commission<br>Bonevski, Billie; Flinders University, College of Medicine & Public Health<br>Milat, Andrew; The University of Sydney, School of Public Health<br>Passey, Megan; The University of Sydney, Daffodil Centre and the University Centre for Rural Health, Faculty of Medicine and Health
**Primary Subject Heading**:	Public health
Secondary Subject Heading:	Research methods
Keywords:	Implementation Science, Behavior, Primary Health Care, Pregnant Women, Change management < HEALTH SERVICES ADMINISTRATION & MANAGEMENT, Quality in health care < HEALTH SERVICES ADMINISTRATION & MANAGEMENT

SCHOLARONE™
Manuscripts

Protocol for the process evaluation of an intervention to improve antenatal smoking cessation support (MOHMQuit) in maternity services in New South Wales, Australia

Jo Longman[1], Chris Paul[2], Aaron Cashmore[3,4], Laura Twyman[5], Larisa AJ Barnes[1,6], Cathy Adams[7], Billie Bonevski[8], Andrew Milat[3,4] and Megan E Passey[1,6]

[1] The University of Sydney, The University Centre for Rural Health, 61 Uralba St., Lismore, NSW 2480, Australia.
[2] University of Newcastle, School of Medicine and Public Health, University Drive, Callaghan, NSW 2308, Australia.
[3] NSW Ministry of Health, Centre for Epidemiology and Evidence, 1 Reserve Road, St Leonards, NSW 2065, Australia.
[4] The University of Sydney, Sydney School of Public Health, Faculty of Medicine and Health, Camperdown, NSW 2006, Australia.
[5] Tobacco Control Unit, Cancer Prevention and Advocacy Division, Cancer Council NSW, and Conjoint Fellow, School of Medicine and Public Health, University of Newcastle, 153 Dowling St., Woolloomooloo, NSW 2011, Australia
[6] The University of Sydney, Daffodil Centre, PO Box 572 Kings Cross NSW 1340, Australia.
[7] Maternity and Neonatal Safety Program; Clinical Excellence Commission, 1 Reserve Road, St Leonards, NSW 2065
[8] Flinders University, College of Medicine & Public Health, Flinders Health and Medical Research Institute, Sturt Road, Bedford Park, SA 5042, Australia

Corresponding author:

Larisa A.J. Barnes
larisa.barnes@sydney.edu.au
Daffodil Centre and the University Centre for Rural Health
Faculty of Medicine and Health
The University of Sydney

For peer review only - http://bmjopen.bmj.com/site/about/guidelines.xhtml

ABSTRACT

Introduction

Smoking cessation in pregnancy remains a public health priority. Our team used the Behaviour Change Wheel to develop the MOHMQuit intervention (Midwives and Obstetricians Helping Mothers to Quit smoking) with health system, leader (including managers and educators) and clinician components. MOHMQuit addresses a critical evidence to practice gap in the provision of smoking cessation support in antenatal care. It involves nine maternity services in New South Wales in a cluster randomised stepped-wedge controlled trial of effectiveness. This paper describes the design and rationale for the process evaluation of MOHMQuit. The process evaluation aims to assess to what extent and how MOHMQuit is being implemented (acceptability; adoption/uptake; appropriateness; feasibility; fidelity; penetration and sustainability), and the context in which it is implemented, in order to support further refinement of MOHMQuit throughout the trial, and aid understanding and interpretation of the results of the trial.

Methods and analysis

The process evaluation is an integral part of the stepped-wedge trial. Its design is underpinned by implementation science frameworks and adopts a mixed methods approach. Quantitative evidence from participating leaders and clinicians in our study will be used to produce individual and site-level descriptive statistics. Qualitative evidence of leaders' perceptions about the implementation will be collected using semi-structured interviews and will be analysed descriptively within-site and thematically across the dataset. The process evaluation will also use publicly-available data and observations from the research team implementing MOHMQuit e.g. training logs. These data will be synthesised to provide site-level as well as individual-level implementation outcomes.

Ethics and dissemination

The study received ethical approval from the Population Health Services Research Ethics Committee for NSW, Australia (Reference 2021/ETH00887). Results will be communicated via the study's Steering Committee and will also be published in peer-reviewed journals and presented at conferences.

Trial registration

Australian New Zealand Trials Registry ACTRN12622000167763
https://www.australianclinicaltrials.gov.au/anzctr/trial/ACTRN12622000167763

Keywords

Implementation science, Behavior, Primary health care, Smoking cessation support, Pregnancy, Antenatal care, Systems change intervention, Stepped-wedge cluster-randomised controlled trial; evaluation studies as topic; process evaluation

For peer review only - http://bmjopen.bmj.com/site/about/guidelines.xhtml

Strengths and limitations of this study

► The process evaluation has been designed using implementation science frameworks

► The study uses multiple data sources. Qualitative and quantitative data will be collected independently from leaders and clinicians in each MOHMQuit site as well as contextual and publicly-available data, and observational data from the research team implementing MOHMQuit

► MOHMQuit is a complex intervention with many moving parts which interact with one another and the stakeholders involved. No process evaluation is able to collect data to understand all aspects of these interactions, particularly not in a 'real world' trial such as this one.

For peer review only

For peer review only - http://bmjopen.bmj.com/site/about/guidelines.xhtml

INTRODUCTION

In 2020, 9.2% of mothers in Australia smoked tobacco at some point during their pregnancy.(1) Smoking in pregnancy is associated with a multitude of adverse outcomes for both mother and baby including pre-term birth and low birth weight babies.(2-5) In Australia, smoking is the most common modifiable risk for adverse pregnancy and birth outcomes(6) and therefore supporting pregnant women to stop smoking remains a major public health concern and a priority for the New South Wales (NSW) Ministry of Health.(7-9) Clinical guidelines for NSW have existed for almost 20 years and recommend clinicians routinely provide evidence-based smoking cessation support (SCS) at all antenatal care visits for women who smoke or who have stopped smoking in this pregnancy.(10) Implementation of the Guidelines shows room for improvement.(11-14) This fact, along with wider evidence that women want to stop smoking in pregnancy but some lack confidence to do so, (15) would value support from their clinicians(16) and a systematic review demonstrating that psychosocial interventions helps women to stop smoking, (17) led us to develop a theoretically underpinned intervention, MOHMQuit (Midwives and Obstetricians Helping Mothers to Quit smoking) to improve implementation of the NSW Guidelines.

The MOHMQuit intervention

The MOHMQuit intervention has multiple components targeting different parts of a complex health system.(18) It is based on the '5As' of SCS: Ask, Advise, Assess, Assist and Arrange Follow-up, which has shown evidence of effectiveness for SCS.(19) MOHMQuit was developed using the Behaviour Change Wheel method.(20) It is an intervention built on local and international evidence identifying barriers and enablers for health systems, leaders and clinicians providing SCS.(21) It focuses on changing behaviours by targeting systems such as the electronic medical record system, leaders and clinicians (see Figure 1 for further detail on what is meant by leaders and clinicians). For example, changing clinicians' behaviours so that they implement the Guidelines by asking about smoking and discussing cessation at every antenatal visit, and assisting women by providing behavioural support such as discussing triggers for smoking, managing nicotine cravings, and planning a quit attempt. The MOHMQuit trial is an implementation trial using a stepped-wedge design across five Local Health Districts in NSW with diverse characteristics including organisational structure and staffing profiles.

Figure 1: Description of key participant groups

The development of the MOHMQuit intervention and its support materials have been described in detail previously.(21 22) In brief, there are four main components (also referred to in the implementation science literature as 'implementation strategies'):

(1) separate training events for maternity service leaders - half day, midwives and AHWs - full day and obstetricians - two hours. Midwifery educators also take part in the leaders' and midwives' training events as a 'train the trainer' model which includes a comprehensive MOHMQuit training manual, is central to the sustainability of the intervention;

(2) a number of MOHMQuit leadership processes and systems tools e.g. a report template for the electronic medical record system facilitating leaders' scrutiny of their services' SCS performance; a service audit tool for leaders;

(3) MOHMQuit written resources such as a booklet on 'Stopping smoking for you and your baby' for clinicians to use with women; and

For peer review only - http://bmjopen.bmj.com/site/about/guidelines.xhtml

(4) a series of 11 short video clips for training and skills development to be used in a wide variety of settings e.g. at handover meetings.

Two months prior to the implementation starting in the first site, a day-long face to face gathering was held bringing together key decision makers and clinicians from across the sites to ensure a shared awareness and understanding of MOHMQuit including its history and rationale, promote enthusiasm, motivation and engagement and establish shared understanding about roles and responsibilities.

At each site, ten weeks prior to the intervention the research team and the maternity service leaders will participate in a 'warm-up' meeting. Whilst each site has a strong existing connection with MOHMQuit via the face to face day, and through the inclusion of partner investigators at each site, the warm-up meeting includes: acknowledging and thanking those involved (which extend beyond the site partner investigators and include the antenatal clinic coordinator, the clinical midwifery educator and other leaders), generating enthusiasm, building momentum in the lead up to the implementation of MOHMQuit, and working through the logistics of implementation at each site. Two weeks prior to the intervention a second meeting will be held which has a 'trouble-shooting' agenda and will also include detail of the research elements of MOHMQuit for example how and when outcome and process evaluation data from the site will be collected. Additional meetings are planned for two and four weeks post-intervention, to maintain momentum and explore any unresolved issues in the ongoing implementation of MOHMQuit. A MOHMQuit Community of Practice will be established which each site can join following implementation. The Community of Practice will offer a regular forum for sharing and supporting other clinicians and leaders in continuing to implement MOHMQuit and is one of several sustainability features of MOHMQuit. Finally, three and a half months after implementation, each site will receive feedback from brief interviews with women about the smoking cessation support they received during their antenatal care. They will continue to receive these reports quarterly until the end of the trial.

MOHMQuit is currently being trialled in a multi-site cluster randomised stepped-wedge effectiveness trial in nine sites in publicly-funded maternity services in NSW, Australia.(23) Implementation is planned to take place over a 13 month timeframe. Unlike many earlier interventions aimed at improving SCS,(24) MOHMQuit is built on implementation science frameworks and is specific to the public maternity service setting. The trial will assess the intervention outcomes. The primary intervention outcome is smoking cessation, and secondary intervention outcomes include changes to clinicians' knowledge, skills, confidence and behaviour in providing SCS and test the 'mechanisms of action'(25-27) by which each of the components effect intervention outcomes and moderators of their impact in this framework-driven approach.(23) Cost-effectiveness will be assessed in an economic evaluation.(28) The trial will also assess key implementation outcomes (assessing how MOHMQuit was implemented) primarily based on Proctor et al's implementation science framework(29) in a detailed process evaluation. The process evaluation will complement the assessment of the MOHMQuit intervention outcomes. Conducting process evaluation alongside effectiveness trials in this way is recommended.(30 31)

Aims of the MOHMQuit process evaluation

Process evaluations explore how an intervention is implemented. They assess three aspects: (a) how and to what extent the intervention was implemented; (b) the 'mechanisms of impact' i.e. how the intervention components and participants' interactions with these components effected changes in behaviour; and (c) the context in which the intervention was implemented.(32) We anticipate that the process evaluation will contribute formatively by providing feedback that may further refine the

For peer review only - http://bmjopen.bmj.com/site/about/guidelines.xhtml

intervention. This is particularly useful in a stepped-wedge trial design where each site joins the trial sequentially, and acceptable as long as the changes made to components retain the integrity of the function they were meant to perform in the original intervention design.(33 34) The summative use of process evaluation is in providing insight into the mechanisms through which the intervention outcomes (the primary intervention outcome being pregnant women stopping smoking), were achieved or not, and therefore it will contribute to understanding and interpreting the results of the effectiveness trial.(35) Without this insight effective, and ineffective, aspects of the intervention may not be understood and this has implications for the scale-up of an intervention such as MOHMQuit. In this way, the process evaluation will maximise the knowledge gained throughout the trial and describe the most effective delivery processes for the MOHMQuit intervention. The aim of this protocol paper is to describe the process evaluation planned as an integral part of the MOHMQuit trial.

METHODS AND ANALYSIS

Overall design and objectives of the process evaluation

The design for the process evaluation began with the implementation outcomes defined by Enola Proctor and team in order to facilitate an understanding of the various dimensions of the implementation: acceptability; adoption/uptake; appropriateness; feasibility; fidelity; penetration and sustainability (and sustainment).(29) Implementation outcomes are "…the effects of deliberate and purposive actions to implement new…practices".(29) The Proctor implementation outcomes generally map on to other well-used frameworks such as the RE-AIM (Reach, Efficacy, Adoption, Implementation, Maintenance) framework (31) but 'Reach' from the RE-AIM framework was specifically added into the design as 'Reach' captures the number of clinicians and leaders invited to and taking part in the trial. Two other frameworks informed the implementation outcomes of interest: Sekhon(36) for acceptability, and Rogers(37) for sustainability, appropriateness and feasibility; and Moore(35) and Fernandez'(38) work guided exploration of mechanisms of impact and how context affected implementation. The context in which the intervention was implemented will also be assessed. Context is variously defined(39) but here contextual features are conceived of broadly as those influencing the delivery of the intervention and include the engagement of leaders and the organisational setting and culture of the service in which the intervention is implemented.(40)

Important features of the process of implementing MOHMQuit were discussed and agreed with a process evaluation working group of the project's Steering Committee (a key governance committee of the project and constituted of research academics, policy makers, managers and leaders(21)). Subsequently, instruments were developed which encompassed both individual and service level data collection. Decisions were made regarding the specific foci s of the process evaluation, acknowledging that "Process evaluations cannot expect to provide answers to all of the uncertainties of a complex intervention. It is generally better to answer the most important questions well than to try to answer too many questions and do so unsatisfactorily." (35)

With that in mind, a focus on fidelity; adoption/uptake; penetration; reach, sustainability and context was agreed. In part these foci were based on learning from the feasibility and acceptability trial of MOHMQuit.(21) In addition, the short duration of the trial (the time from implementation at the first site to the end of data collection, excluding the wash out period, is 24 months and from the final site, only 8 months) would make sustainment challenging to measure. Sustainability is, however, included in the evaluation. Sustainment is "the continued use of a practice that is the

For peer review only - http://bmjopen.bmj.com/site/about/guidelines.xhtml

target of the implementation, whereas sustainability addresses whether the factors are in place to promote the ongoing use." (41)

The process evaluation has three interrelated objectives; to, at both the individual and site level, assess:

1. To what extent MOHMQuit was implemented - measured quantitatively focusing on the implementation outcomes of adoption, fidelity, penetration, reach and sustainability, and will also involve qualitative measures (interviews with leaders)
2. How changes in behaviour were effected (the mechanisms of impact) – measured quantitatively focusing on the implementation outcomes of acceptability, appropriateness and feasibility, and a more nuanced understanding of this from leaders' perspectives in qualitative interviews
3. The impact of context (moderators) on the implementation of MOHMQuit. A moderator is a factor that will strengthen or lessen the influence of a strategy to implement MOHMQuit.(26) We anticipate a number of moderators will be an important part of the context for MOHMQuit implementation, as well as intervention outcomes, affecting the relationship between the implementation outcomes e.g. reach, and the implementation of MOHMQuit. The moderators measured include:

    a. Leadership
        i. Leaders self-assessment of their leadership for implementation at 3 months using the Implementation Leadership Scale(42)
        ii. Clinicians questionnaires at 6 months which include the Leadership Engagement Scale(38)
    b. Implementation climate
        i. Clinician questionnaires at 6 months which include the Implementation Climate Scale(38)
    c. Service Size
    d. Smoking prevalence amongst pregnant women birthing at that site
    e. Other demands on leaders/service e.g. new SCS policies and training or accreditation

In summary, we speculate that the impact of the context on the implementation outcomes could be as follows:

- Leadership and implementation climate - impacting on all outcomes
- Service size, smoking prevalence and models of care - impacting on adoption, appropriateness, feasibility, penetration and sustainability
- Other demands on leaders - impacting on implementation in terms of adoption, fidelity, penetration and sustainability

see Figure 2 below which summarises our speculation about which of each of the context elements might impact on each of the implementation outcomes e.g. we anticipate leadership will impact on all of the implementation outcomes.

For peer review only - http://bmjopen.bmj.com/site/about/guidelines.xhtml

Figure 2: Speculating which context elements may impact on each of the implementation outcomes

For peer review only

For peer review only - http://bmjopen.bmj.com/site/about/guidelines.xhtml

Recruitment and consent

The Local Health Districts (LHDs – which manage public hospitals and provide healthcare services in a defined geographic area) in NSW with relatively high rates of smoking in pregnancy were approached to participate in the MOHMQuit trial. There are 15 LHDs in total, seven with high smoking rates in pregnancy were invited and five agreed to participate in the trial. Between them they selected nine maternity services (sites) to take part. The senior midwives and lead obstetricians from these five LHDs were partner investigators in a Partnership Grant application subsequently awarded by Australia's National Health and Medical Research Council and so their involvement with the project substantially precedes the implementation trial of MOHMQuit.

Individual service leaders and clinicians in each of the nine sites will be provided with a Participant Information Sheet and those who agree to participate in the research will be asked to sign a written consent form indicating their consent to take part in data collection. This consent applies to data collection to measure the implementation outcomes and context as well as the intervention outcomes.

Process evaluation data collection

The process evaluation will adopt a mixed methods approach, collecting quantitative evidence from questionnaires and qualitative evidence of leaders' perceptions of how MOHMQuit may have changed behaviour (where it was perceived to have done so) from semi-structured interviews. Data will be collected by the research team independently from each of the nine MOHMQuit sites. Study-specific questionnaires will be used to collect implementation outcome data from leaders and clinicians at each site at various time points: immediately following training, three months after the training and six months after the training as outlined below. To minimise participant burden, the questionnaires will also collect the data required to measure the intervention outcomes.

Qualitative data will be collected using semi-structured interviews with leaders six months after the training at each site. The interviews have three key purposes. Firstly, interviews will collect data on the components of MOHMQuit which have been implemented in the six months following the MOHMQuit training (uptake) e.g. use of the report template for the electronic medical record system facilitating leaders' scrutiny of their services' SCS performance for feedback and continuous improvement, or MOHMQuit training delivered by the service themselves using the train the trainer manual. Secondly, they will collect data to support the calculation of an implementation cost as part of the detailed economic evaluation of MOHMQuit, the subject of a separate paper, (28) by recording how much time leaders' assess they spend implementing those components of MOHMQuit. Finally, they will collect data which will enhance the contextual information collected by the research team by eliciting leaders' perspectives of the enablers and barriers of the implementation of MOHMQuit and what might be improved with regard to it. Interviews will be conducted using the Teams platform, recorded and transcribed. They will be guided by an interview schedule driven by the implementation outcomes and the contextual factors that supported or hindered implementation and any adaptations made to the intervention. The semi-structured nature of the interviews will allow for flexibility in questioning and expansion on responses.

Data collection from leaders and antenatal care clinicians will be as follows:

Leaders

- An online questionnaire to all leaders **three months after** the training at each site regardless of whether they attended MOHMQuit training (anticipated numbers of leaders who will be invited approximately 55) .

For peer review only - http://bmjopen.bmj.com/site/about/guidelines.xhtml

- A semi-structured one to one telephone interview **six months after** the training with the midwifery partner investigator and one to two other leaders at each site.

Antenatal care clinicians

- A paper questionnaire **immediately following the training** at each site to participants who attended training (anticipated numbers of participants who will be invited approximately 250).
- An online questionnaire to all antenatal care clinicians and AHWs **six months after** the training at each site regardless of whether they attended MOHMQuit training (anticipated numbers of participants who will be invited approximately 300).

In addition, attendance and fidelity information (which aspects of the training were delivered) will be kept by the research team during each training event and the attendance and engagement at various meetings that are components of MOHMQuit. The additional data collection includes:

- Training logs – to calculate proportion attended at each training event (attendance/invited)
- A 'fidelity checklist' of which elements of the training were covered during each training event
- Attendance and notes from 10 week warm-up meetings
- Attendance and notes from 2 week warm-up meetings
- Attendance and notes from 2 week follow-up meetings
- Attendance and notes from 4 week follow-up meetings
- Attendance and notes from monthly Community of Practice meetings

For each site a 'context table' will be completed by the research team using publicly available sources and with input from partner investigators at each site (Table 1).

Table 1: Key contextual information collected for each site

**Number of births at site 2020**
**Smoking prevalence 2020**
**Performance against the NSW Ministry of Health's performance indicator of antenatal smoking**
**Safer Baby Bundle at site?* (Yes/No)**
**Preparation and training for new NSW Maternity Care Policy (RSVP)# overlaps with MOHMQuit timing? (Yes/No)**
**Other SCS initiatives running at the site? (Yes/No)**
**Accreditation for Quality Improvement going on concurrent with MOHMQuit? (Yes/No)**
**Leadership structure at the site**
**Models of care offered and proportion of women at booking and at birth for each model**
**Other e.g. external events like disasters, vacant posts**

* Safer Baby Bundle is a multi-component intervention in maternity service which aims to reduce the number of preventable stillbirths

# The RSVP policy is a policy directive establishing minimum requirements for health services to provide evidence-based smoking cessation support to women before during and after pregnancy. The RSVP Policy was released 14 October 2022.

We anticipate that the data collection itself may have the beneficial sustainability effect of reminding leaders and clinicians about MOHMQuit and possibly prompting renewed attention and/or commitment to it.

For peer review only - http://bmjopen.bmj.com/site/about/guidelines.xhtml

Table 2 provides detail of working definitions and how each of the implementation outcomes and contextual features will be measured at which timepoints, using which instruments with whom, and which strategies (components of the MOHMQuit intervention) are aimed to maximise the implementation outcomes. Further detail is available in Supplementary Table 1.

For peer review only

For peer review only - http://bmjopen.bmj.com/site/about/guidelines.xhtml

Table 2: Implementation outcomes, definitions, strategies for maximising implementation outcomes, frameworks used and measurement items

Implementation outcome	Strategies used to maximise implementation outcomes	Frameworks used	Data collection instruments, timing, participants
Adoption/Uptake (intention or action to try to employ MOHMQuit)	• Warm-up and follow-up meetings<br>• Community of Practice	Proctor(29)<br>RE-AIM(31)<br>(Adoption)	• Attendance at warm-up and follow up meetings<br>• 3 months post-training - questionnaire with leaders<br>• 6 months post-training - questionnaire with clinicians<br>• 6 months post- training - interview with leaders<br>• Community of Practice peer support meetings attendance
Fidelity (delivered as intended in the Protocol(23), adherence)	• Warm-up and follow-up meetings<br>• Consistency in the team delivering MOHMQuit training at each site in the first instance<br>• Clear plans and materials for content of training	Proctor(29)<br>RE-AIM(31)<br>(Implementation)	• Attendance at warm-up and follow up meetings<br>• Training logs of expected and actual attendance at training of leaders and clinicians<br>• Fidelity record (which aspects of the planned training were actually delivered)<br>• 6 months post- training - interview with leaders
Penetration (degree of integration of MOHMQuit practices within the service)	• Involving leaders in the training for clinicians for a whole-of-service approach<br>• MOHMQuit leadership components include repeated audit and feedback plus action planning; developing and implementing a clinical pathway for SCS; and the development and maintenance of SCS 'champions' within each service<br>• Train the trainer model an integral part of the intervention	Proctor(29)<br>RE-AIM(31)<br>(Adoption)	• 3 months post-training - questionnaire with leaders<br>• 6 months post-training - interview with leaders
Reach (did MOHMQuit include everyone that it aimed to?)	• 10-week warm-up meetings to allow time for planning and rostering<br>• The train the trainer model as an integral part of the intervention to support participation of all relevant existing and new staff	RE-AIM(35)	• Training logs of expected and actual attendance at training of leaders and clinicians recorded at the time of training<br>• 3 months post-training - questionnaire with leaders<br>• 6 months post-training - interview with leaders
Sustainability (factors promoting ongoing use of MOHMQuit)	• MOHMQuit leadership components include repeated audit and feedback plus action planning; developing and implementing a clinical pathway for SCS; and development and maintenance of SCS 'champions' within each service<br>• Train the trainer model an integral part of the intervention<br>• The Community of Practice	Proctor(29)<br>RE-AIM(31)<br>(Maintenance)<br>Rogers(37)	• 6 months post-training - questionnaire with clinicians<br>• 6 months post-training - interview with leaders<br>• Community of Practice peer support attendance data

For peer review only - http://bmjopen.bmj.com/site/about/guidelines.xhtml

Implementation outcome	Strategies used to maximise implementation outcomes	Frameworks used	Data collection instruments, timing, participants
Acceptability (how palatable is MOHMQuit to clinicians and leaders?)	• Comprehensive systematic design of MOHMQuit using the Behaviour Change Wheel with input from clinicians and leaders(21 22)<br>• Feasibility and acceptability trial with subsequent minor amendments to the intervention(21)<br>• 10 week warm-up includes the history of MOHMQuit so leaders are reassured about its quality, relevance and acceptability	Proctor(29)<br>Sekhon(36)	• Immediately post-training - questionnaire with clinicians<br>• 3 months post-training - questionnaire with leaders<br>• 6 months post-training - questionnaire with clinicians<br>• 6 months post- training - interview with leaders
Appropriateness (perceived fit or relevance of MOHMQuit with the service)	• Comprehensive and systematic design of MOHMQuit using the Behaviour Change Wheel integrating input from clinicians and leaders(21 22)<br>• Feasibility and acceptability trial with subsequent minor amendments to the intervention(21)<br>• 10 week warm-up includes the history of MOHMQuit so leaders are reassured about its quality, relevance and acceptability	Proctor(29)<br>Rogers(37)	• 6 months post-training - interview with leaders
Feasibility (actual fit – the extent to which MOHMQuit can be integrated into usual care in a service)	• Comprehensive and systematic design of MOHMQuit using the Behaviour Change Wheel integrating input from clinicians and leaders(21 22)<br>• Feasibility and acceptability trial with subsequent minor amendments to the intervention(21)<br>• 10 week warm-up includes the history of MOHMQuit so leaders are reassured about its quality, relevance and acceptability	Proctor(29)<br>Rogers(37)	• 3 months post-training - questionnaire with leaders<br>• 6 months post-training - interview with leaders
HOW behaviour was changed		Moore(35)	• 6 months post-training - interview with leaders
**HOW context affected implementation**	• **Commitment of maternity service leaders in the research as Partner Investigators and members of MOHMQuit Steering Committee and various working groups**<br>• **Warm-up meetings and follow up meetings**<br>• **Community of Practice**	**Fernandez(38)**	• **Key contextual information (Table 1) completed by research team during the implementation**<br>• **3 months post-training - questionnaire with leaders**<br>• **6 months post-training - questionnaire with clinicians**<br>• **6 months post-training - interview with leaders**

BOLD TYPEFACE INDICATES OUTCOMES THAT WILL BE THE FOCUS OF THE PROCESS EVALUATION

Implementation cost is not included in Table 2 as a detailed economic evaluation of MOHMQuit is taking place and is the subject of a separate paper.(28) Data to contribute to the economic evaluation will be collected as part of the semi-structured interview with leaders.

For peer review only - http://bmjopen.bmj.com/site/about/guidelines.xhtml

Patient and public involvement

As this is an implementation science trial, our partners in identifying the need for the study and in its design and implementation were health service clinicians, leaders and policy makers.(21) Patients were not involved in designing or implementing the research, but are participants in the trial(23) but not in the process evaluation.

Data analysis

We will assess each of the implementation outcomes (Table 2) for each site, including assessing variation across the nine sites. At this stage it is not possible to definitively describe which of the implementation outcomes our analyses will be focused on as that will depend on the variation in implementation outcomes across sites. For example, if there is little variation in fidelity it will not help explain the MOHMQuit (intervention) outcomes. However, where appropriate descriptive statistics (measures of central tendency, standard deviations and proportions) will be produced using data from questionnaire responses from clinicians and leaders to summarise quantitative results by participant and by site.

Analyses for the moderators will include calculation of a measure of central tendency, for the leadership(42) sub-scales for each participant. There are four subscales: the proactive subscale, the knowledgeable subscale, the supportive subscale, and the perseverant subscale. A measure of central tendency for each set of items that load onto the relevant subscale will be calculated for each subscale. A measure of central tendency of the scale scores will be calculated which will provide a total score for the Implementation Leadership Scale.(42) In addition, scores will be aggregated to provide a site-level score. We do not anticipate adding these results, or any of the data from Table 1 to any model but they will help constitute a broader assessment of the context for implementation to contribute to understanding of in which sites, and how, MOHMQuit was effective.

Qualitative data from semi-structured interviews with leaders will be analysed descriptively to explore perspectives of uptake by site and thematically across all sites regarding the enablers, barriers and how implementation of MOHMQuit might be improved. Thematic analysis will follow the steps outlined by Braun and Clarke: data familiarisation; initial generation of codes; development of themes by collating codes and then reviewing the raw data again to check the material sits together coherently as a theme; defining each theme and how themes work together to tell the overall story of the data).(43)

Data from multiple sources will facilitate triangulation, for example collecting data about acceptability from quantitative data (post-training questionnaires from clinicians and questionnaires at six months from all clinicians) along with qualitative interviews with leaders from each site. This mixed methods approach will broaden and deepen understanding of the results of the trial. The key findings will be presented in an integrated way using a side-by-side joint display table(44) each source being given equal weight.

Figure 3 below describes this visually.

For peer review only - http://bmjopen.bmj.com/site/about/guidelines.xhtml

Figure 3: Mixed methods approach to data collection and analysis

For peer review only

For peer review only - http://bmjopen.bmj.com/site/about/guidelines.xhtml

Ethical considerations and dissemination

The process evaluation received ethical approval from the NSW Population Health Services Research Ethics Committee (Reference Number 2021/ETH00887) on July 23rd, 2021. Results of the process evaluation will be written up for publication in peer-reviewed journals and presented at conferences. The process evaluation will perform a formative function facilitated by the stepped-wedge design with sites receiving the intervention in a staggered implementation, allowing for further polishing of the intervention as the trial proceeds. The process evaluation will also provide contextual information to elucidate the findings of the trial in terms of how MOHMQuit may have been effective in some sites but not in others. This understanding is critical in relation to rolling out MOHMQuit across NSW should the intervention prove to be effective.

Trial registration number

The MOHMQuit trial is registered with ANZCTR (www.anzctr.org.au): ACTRN12622000167763.

DISCUSSION

Implementation science is the study of approaches that support the systematic uptake of research findings into 'usual care'.(45) In cases where there is an urgent need for behaviour change and a clear evidence to practice gap, such as with SCS in antenatal care, implementation science provides a framework for examining an intervention such as MOHMQuit. This paper describes the mixed-methods design and underpinning frameworks for the process evaluation of MOHMQuit as part of an implementation science study. MOHMQuit is a complex multi-component intervention designed using the Behaviour Change Wheel.(20) It aims to change the behaviour of antenatal care providers to improve the support provided to women to stop smoking in pregnancy. MOHMQuit is being implemented in a stepped-wedge effectiveness trial across nine publicly funded maternity services in NSW.(23)

The process evaluation will facilitate the ongoing refinement of MOHMQuit and will provide an assessment of the extent to which MOHMQuit was implemented, what the mechanisms of impact were and what the context of implementation was, and how it affected the implementation of MOHMQuit. It will also inform other components of the study for example contributing data to support costing of MOHMQuit for the economic evaluation. We anticipate that the findings from the process evaluation will contextualise and aid understanding of our trial results, and may support the further implementation of MOHMQuit in NSW. For example, if it transpires that implementation leadership is more evident in those sites where MOHMQuit was shown to be particularly effective, the scale-up would need to include a focus on *implementation leadership* and on implementing the leadership components of the intervention. Our process evaluation will also contribute knowledge about the implementation of stepped-wedge trials which may be useful to others in the future. Whilst we have described our intended approach to evaluating the implementation of MOHMQuit, we have also included flexibility of approach in recognition of unanticipated implementation factors that may surface.(40)

Smoking in pregnancy is an ongoing public health challenge and represents a considerable gap between the evidence for smoking cessation support and practice. Providing a broader understanding of how MOHMQuit was or was not effective will be key to its potential future roll-out/scale up.

For peer review only - http://bmjopen.bmj.com/site/about/guidelines.xhtml

Empirical testing of the theory

Implementation science is a relatively new academic endeavour and this process evaluation has the potential to contribute to a growing body of evidence of approaches to implementing comprehensive stepped-wedge trial designs that are inclusive of process evaluation.

Strengths and limitations

The process evaluation has been designed using implementation science frameworks and explores the implementation of MOHMQuit, a thorough and theoretically underpinned intervention and trial design.(21) The results of the trial will provide further evidence for the effectiveness, or otherwise, of this theoretically driven approach. The mixed methods approach in the process evaluation includes qualitative and quantitative data collection from a wide range of leaders and clinicians in each MOHMQuit site, some of whom will not have directly participated in the MOHMQuit training, as well as publicly-available data and observational data from the research team implementing MOHMQuit. This approach has the potential to produce findings that have depth and nuance and will aid understanding of the trial findings. However, MOHMQuit is a complex intervention with many moving parts which interact with one another, and the stakeholders involved. No process evaluation is able to collect data to understand all aspects of these interactions. In addition, the MOHMQuit trial is a 'real world' trial. This has strengths in producing findings that can be confidently understood as realistic, however it also produces many challenges including the potential impact of new policies and procedures, staffing issues etc. many of which we have aimed to record as part of the process evaluation but some of which we are likely to have missed. This may compromise our capacity to fully understand and accurately interpret the intervention outcomes.

Trial status

Recruitment for the trial is underway. Process evaluation data collection commenced in March 2023 and will conclude in May 2024.

For peer review only

For peer review only - http://bmjopen.bmj.com/site/about/guidelines.xhtml

ACKNOWLEDGEMENTS

This paper is submitted on behalf of the MOHMQuit Trial team, including all chief investigators, partner investigators and associate investigators, and co-researchers and site leads at each of the MOHMQuit sites. In addition to the named authors, the team includes Dheya Al Mashat (NSW Health), Dianne Avery (NSW Health), Elizabeth Best (NSW Ministry of Health), Alecia Brooks (Cancer Council NSW), Rashna Chinoy (NSW Health), Justine Elliot (NSW Health), Jacinta Felsch (NSW Health), Mohamed Foda (NSW Health), Sandra Forde (NSW Health), Tara Farrugia (NSW Health), Tracey Greenberg (Alcohol and Drug Service, St Vincent's Hospital Sydney), Jane Griffith (NSW Health), Madeline Hubbard (NSW Health), Damien McCaul (NSW Ministry of Health), James McLennan (Alcohol and Drug Service, St Vincent's Hospital Sydney), Kate Reakes (Cancer Institute NSW), Virginia Stulz (NSW Health and Western Sydney University), Tracey Zakazakaarcher (NSW Health), and Lou Atkins (University College London) who provided excellent early guidance on the process evaluation design.

AUTHORS' CONTRIBUTIONS

The process evaluation was conceived and designed by all authors: MP, JL, CP, LT, LB, CA, BB, AC and AM . The first draft of the paper was written by JL with input from MP and CP before receiving input from all other authors. All authors have read and approved the final manuscript.

FUNDING

This work is currently supported by a partnership grant from the NHMRC (GNT1185261). Previously, NHMRC funding (GNT1072213) and the Cancer Institute NSW funding (13/ECF/1-11) supported the developmental work of MOHMQuit. MP was also supported during this time by a fellowship grant from the NHMRC (GNT1159601). The funding bodies did not have any role in the design of the study and collection, analysis and interpretation of data.

Ethics approval and consent to participate

Ethics approval for the research was received from the Population Health Services Research Ethics Committee (Reference Number 2021/ETH00887), on July 23rd, 2021. All potential participants will be provided with a Participant Information Sheet (PIS). A signed (written) consent form will be obtained by site trial staff for all maternity service leaders and clinicians who participate in the trial.

Clinicians: Participation of clinicians (anonymous survey participation) is voluntary. Participant Information Sheets for clinicians will explicitly state that the decision to participate or not participate will not influence their professional standing or the care of any of their patients/clients in any way.

Maternity service leaders: The participation of maternity service leaders (semi-structured interviews) is voluntary. The Participant Information Sheet for leaders will explicitly state that the decision to participate or not participate will not influence their professional standing or the care of any of their patients/clients in any way. The Participant Information Sheet will also detail that information shared in interviews will be de-identified before publication or dissemination.

Availability of data and materials

Not applicable

COMPETING INTERESTS

None declared.

For peer review only

For peer review only

For peer review only - http://bmjopen.bmj.com/site/about/guidelines.xhtml

References

1. Australian Institue of Health and Welfare. Australia's mothers and babies 2020, web report - Summary Canberra: Australian Government; 2022 [Available from: https://www.aihw.gov.au/reports/mothers-babies/australias-mothers-babies/contents/antenatal-period/smoking-during-pregnancy accessed 23 February 2023.
2. Diamanti A, Papadakis S, Schoretsaniti S, et al. Smoking cessation in pregnancy: An update for maternity care practitioners. *Tobacco induced diseases* 2019;17:57. doi: https://doi.org/10.18332/tid/109906
3. Lange S, Probst C, Rehm J, et al. National, regional, and global prevalence of smoking during pregnancy in the general population: a systematic review and meta-analysis. *The Lancet Global Health* 2018;6(7):e769-e76. doi: https://doi.org/10.1016/S2214-109X(18)30223-7
4. Avşar TS, McLeod H, Jackson L. Health outcomes of smoking during pregnancy and the postpartum period: an umbrella review. *BMC Pregnancy and Childbirth* 2021;21(1):254. doi: 10.1186/s12884-021-03729-1
5. US Department of Health and Human Services. The Health Consequences of Smoking: 50 Years of Progress. A Report of the Surgeon General. Atlanta, GA: U.S. Department of Health and Human Services, Centers for Disease Control and Prevention, National Center for Chronic Disease Prevention and Health Promotion, Office on Smoking and Health, 2014.
6. Greenhalgh E, Ford C, Winstanley M. Section 3.7 Pregnancy and smoking Melbourne, Victoria: Cancer Council; 2021 [Available from: https://www.tobaccoinaustralia.org.au/chapter-3-health-effects/3-7-pregnancy-and-smoking accessed January 2024.
7. U.S. Department of Health & Human Services. Smoking Cessation: A Report of the Surgeon General. Rockville: US Department of Health and Human Services, 2020.
8. NSW Ministry of Health. The First 2000 Days Framework. In: Health and Social Policy Branch, ed. Sydney, Australia, 2019.
9. NSW Ministry of Health. First 2000 Days Implementation Strategy 2020-2025. St Leonards: NSW Ministry of Health, 2021.
10. NSW Ministry of Health. NSW Clinical Guidelines for the Management of Substance Use During Pregnancy, Birth and the Postnatal Period. Sydney: NSW Ministry of Health, 2014.
11. Passey ME, Longman JM, Adams CM, et al. Factors associated with provision of smoking cessation support to pregnant women – a cross-sectional survey of midwives in New South Wales, Australia. *BMC Pregnancy and Childbirth* 2020;20:219.
12. Longman JM, Adams CM, Johnston JJ, et al. Improving implementation of the smoking cessation guidelines with pregnant women: How to support clinicians? *Midwifery* 2018;58:137-44. doi: https://doi.org/10.1016/j.midw.2017.12.016
13. Passey M, Sanson-Fisher RW. Provision of antenatal smoking cessation support: a survey with pregnant Aboriginal and Torres Strait Islander women. *Nicotine Tob Res* 2015;17(6):746-49.
14. Bar Zeev Y, Bonevski B, Twyman L, et al. Opportunities Missed: A Cross-sectional Survey of the Provision of Smoking Cessation Care to Pregnant Women by Australian General Practitioners and Obstetricians. *Nicotine & tobacco research: official journal of the Society for Research on Nicotine and Tobacco* 2017;19(5):636-41.
15. Hoekzema L, Werumeus Buning A, Bonevski B, et al. Smoking rates and smoking cessation preferences of pregnant women attending antenatal clinics of two large Australian maternity hospitals. *Aust N Z J Obstet Gynaecol* 2014;54(1):53-8.
16. Passey ME, Sanson-Fisher RW, Stirling JM. Supporting pregnant Aboriginal and Torres Strait Islander women to quit smoking: views of antenatal care providers and pregnant Indigenous women. *Matern Child Health J* 2014;18:2293-99.
17. Chamberlain C, O'Mara-Eves A, Porter J, et al. Psychosocial interventions for supporting women to stop smoking in pregnancy. *The Cochrane Library* 2017(2) doi: 10.1002/14651858.CD001055.pub5.

18. Skivington K, Matthews L, Simpson SA, et al. A new framework for developing and evaluating complex interventions: update of Medical Research Council guidance. *bmj* 2021;374
19. U.S. Department of Health and Human Services. Smoking Cessation. A Report of the Surgeon General. Atlanta GA: U.S. Department of Health and Human Services, Centers for Disease Control and Prevention, National Center for Chronic Disease Prevention and Health Promotion, Office on Smoking and Health, 2020.
20. Michie S, van Stralen MM, West R. The behaviour change wheel: A new method for characterising and designing behaviour change interventions. *Implement Sci* 2011;6:42.
21. Passey ME, Adams C, Paul C, et al. Improving implementation of smoking cessation guidelines in pregnancy care: Development of an intervention to address system, maternity service leader and clinician factors. *Implementation Science Communications* 2021;2:128. doi: https://doi.org/10.1186/s43058-021-00235-5
22. Longman J, Adams C, Paul C, et al. Improving clinicians' implementation of guidelines to help women stop smoking in pregnancy: developing evidence-based print and video materials. *Int J Environ Res Public Health* 2021;18(19):10522. doi: https://doi.org/10.3390/ijerph181910522
23. Barnes LAJ, Longman J, Adams C, et al. The MOHMQuit (Midwives and Obstetricians Helping Mothers to Quit Smoking) Trial: protocol for a stepped-wedge implementation trial to improve best practice smoking cessation support in public antenatal care services. *Implement Sci* 2022;17(1):79. doi: 10.1186/s13012-022-01250-3
24. Bar-Zeev Y, Bonevski B, Lim LL, et al. Improving health providers smoking cessation care in pregnancy: A systematic review and meta-analysis. *Addict Behav* 2019;93:29-38. doi: https://doi.org/10.1016/j.addbeh.2019.01.002
25. Lewis CC, Boyd MR, Walsh-Bailey C, et al. A systematic review of empirical studies examining mechanisms of implementation in health. *Implement Sci* 2020;15:1-25.
26. Lewis CC, Klasnja P, Powell BJ, et al. From classification to causality: advancing understanding of mechanisms of change in implementation science. *Frontiers in public health* 2018;6:136.
27. Springer MV, Sales AE, Islam N, et al. A step toward understanding the mechanism of action of audit and feedback: a qualitative study of implementation strategies. *Implement Sci* 2021;16:1-13.
28. Pearce A, Scarfe J, Jones M, et al. Study protocol of an economic evaluation embedded in the Midwives and Obstetricians Helping Mothers to Quit Smoking (MOHMQuit) trial. *BMC Health Serv Res* 2023;23(1):1-9.
29. Proctor E, Silmere H, Raghavan R, et al. Outcomes for implementation research: conceptual distinctions, measurement challenges, and research agenda. *Administration and policy in mental health and mental health services research* 2011;38(2):65-76.
30. Graham Moore SA, Barker M, Bond L, et al. Process evaluation of complex interventions. *Complex Interventions in Health: An overview of research methods* 2015:222.
31. Glasgow RE, Vogt TM, Boles SM. Evaluating the public health impact of health promotion interventions: the RE-AIM framework. *Am J Public Health* 1999;89(9):1322-27.
32. Global Alliance for Chronic Diseases. GACD Process Evaluation Guidelines, 2015 [Available from: https://www.gacd.org/perch/resources/gacd-pe-guidelines-v1-0.pdf.
33. Hawe P, Shiell A, Riley T. Complex interventions: how "out of control" can a randomised controlled trial be? *Bmj* 2004;328(7455):1561-63.
34. Kirk MA, Moore JE, Wiltsey Stirman S, et al. Towards a comprehensive model for understanding adaptations' impact: the model for adaptation design and impact (MADI). *Implement Sci* 2020;15:1-15.
35. Moore GF, Audrey S, Barker M, et al. Process evaluation of complex interventions: Medical Research Council guidance. *bmj* 2015;350
36. Sekhon M, Cartwright M, Francis JJ. Acceptability of healthcare interventions: an overview of reviews and development of a theoretical framework. *BMC Health Serv Res* 2017;17(1):88.

For peer review only

37. Rogers EM. Diffusion of Innovations. 4th ed. New York: The Free Press 1995.
38. Fernandez ME, Walker TJ, Weiner BJ, et al. Developing measures to assess constructs from the Inner Setting domain of the Consolidated Framework for Implementation Research. *Implement Sci* 2018;13(1):52. doi: 10.1186/s13012-018-0736-7
39. Craig P, Di Ruggiero E, Frolich KL, et al. Taking account of context in population health intervention research: guidance for producers, users and funders of research. . Canadian Institutes of Health Research (CIHR)–National Institute for Health Research (NIHR) Southampton: National Institute for Health Research, 2018.
40. Beames JR, Lingam R, Boydell K, et al. Protocol for the process evaluation of a complex intervention delivered in schools to prevent adolescent depression: the Future Proofing Study. *BMJ open* 2021;11(1):e042133.
41. Moullin JC, Sklar M, Ehrhart MG, et al. Provider REport of Sustainment Scale (PRESS): development and validation of a brief measure of inner context sustainment. *Implement Sci* 2021;16(1):1-10.
42. Aarons GA, Ehrhart MG, Farahnak LR. The Implementation Leadership Scale (ILS): Development of a Brief Measure of Unit Level Implementation Leadership. *Implement Sci* 2014;9:45. doi: 10.1186/1748-5908-9-45
43. Braun V, Clarke V. Using thematic analysis in psychology. *Qualitative research in psychology* 2006;3(2):77-101.
44. Guetterman TC, Fetters MD, Creswell JW. Integrating quantitative and qualitative results in health science mixed methods research through joint displays. *The Annals of Family Medicine* 2015;13(6):554-61.
45. Eccles M, Mittman B. Welcome to implementation science [Internet]. Vol. 1. *Implementation Science BioMed Central* 2006:1.

For peer review only

**Antenatal Care clinicians**	• Antenatal care midwives<br>• Aboriginal Health Workers (AHWs) - primary healthcare workers who ensure culturally safe maternity care in supporting Aboriginal and/or Torres Strait Islander women or women having an Aboriginal baby<br>• Obstetricians (staff specialists; Visiting Medical Officers with specialist obstetric training, Career Medical Officers) and obstetric registrars
**Leaders**	Maternity service leaders (those who support or supervise health professionals providing antenatal care), including:<br>• Clinical Midwifery Consultants<br>• Maternity Unit Managers<br>• Clinical Midwifery Educators<br>• Clinical Midwifery Specialists<br>• Antenatal clinic coordinators<br>• Obstetric leads

For peer review only

	Implementation outcomes							
**Context measures**	Acceptability	Adoption	Appropriateness	Feasibility	Fidelity	Penetration	Sustainability	Reach
Leadership								
Implementation climate								
Service size								
Smoking prevalence								
Models of care								
Other demands on leaders								

For peer review only

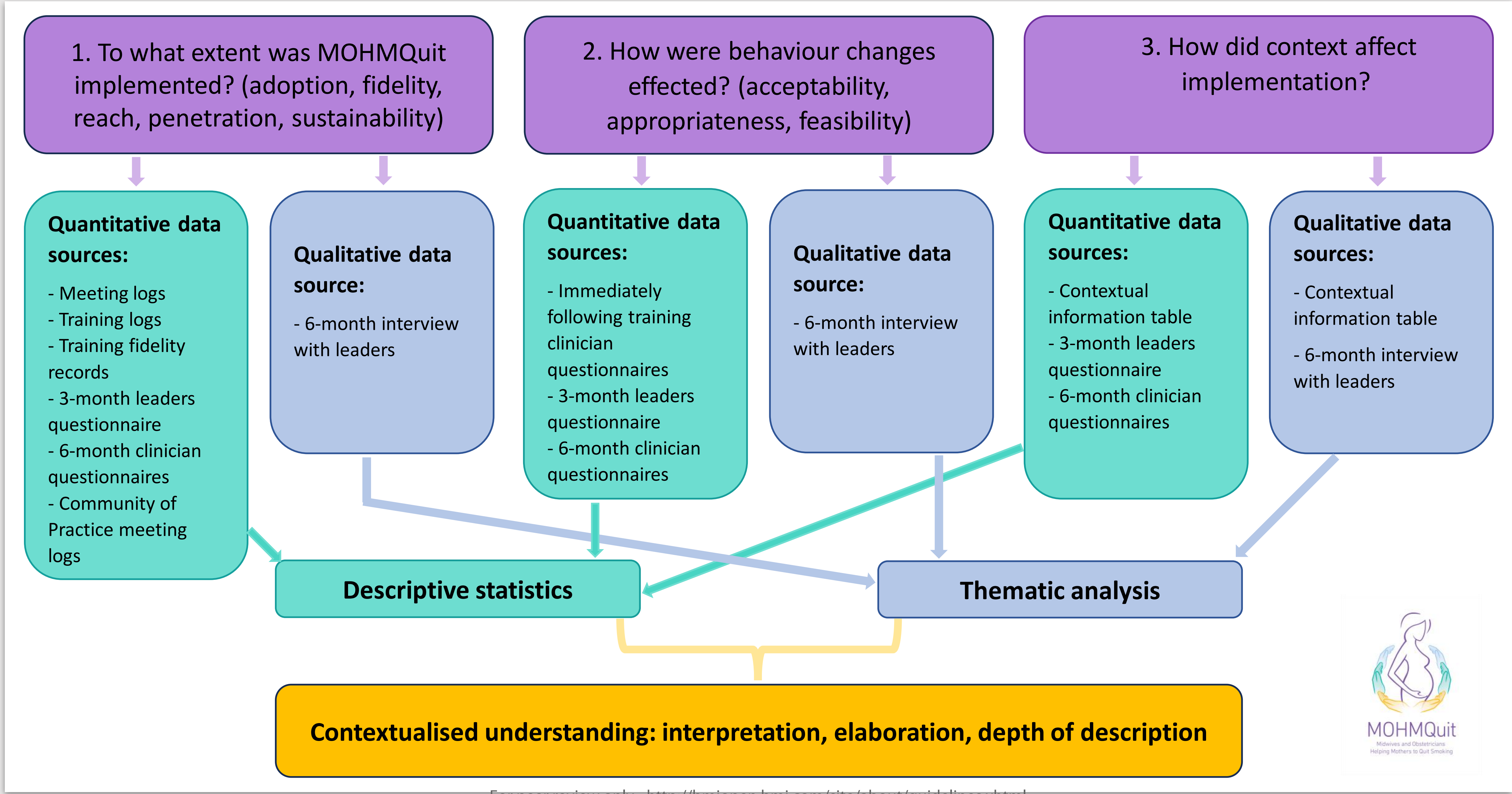

For peer review only - http://bmjopen.bmj.com/site/about/guidelines.xhtml

Supplementary Table 1: Implementation outcomes, definitions, strategies for maximising implementation outcomes, frameworks used and measurement items

Implementation outcome (abbreviated definition)	Strategies used to maximise implementation outcomes	Level of analysis	Frameworks used	Data collection instruments, timing, participants	Items from instruments
**Adoption/Uptake (intention or action to try to employ MOHMQuit)**	• Warm-up meetings<br>• Follow up meetings<br>• Community of Practice	Site level<br>Individual clinician level	Proctor[1]<br>RE-AIM[2]<br>(Adoption)	• Warm-up and follow up meetings<br><br>• 3 months following training - questionnaire for leaders<br><br>• 6 months following training - questionnaire with clinicians<br><br>• 6 months following training - semi-structured interview with leaders<br><br>• Community of Practice peer support meetings attendance data	<u>Meetings</u><br>* Whether the 10 and 2-week warm-up meetings took place/were attended;<br>* Whether the 2 and 4 week post training meetings took place/were attended<br><u>3 month questionnaire for leaders</u><br>* *In the last 3 months did you or any other staff in your service…* (followed by a number of MOHMQuit components)*?*<br><u>6 month questionnaire for clinicians</u><br>* *How useful were each of the MOHMQuit resources when working with women (scale of 1-3 Very useful to Not at all useful + Not Applicable as a response option)?*<br><u>6 month interview guide for leaders</u><br>* *Have you, or any of your colleagues implemented any of the components of MOHMQuit* (followed by a number of MOHMQuit components)*?*<br>* *What do you think helped in delivering MOHMQuit?*<br><br><u>Community of Practice meetings</u><br>* Sites attending community of practice meetings
**Fidelity (delivered as intended in the Protocol[3], adherence)**	• Warm-up and follow-up meetings<br>• Consistency in the team delivering MOHMQuit training at each site in the first instance	Site level	Proctor[1]<br>RE-AIM[2]<br>(Implementation)	• Warm-up and follow up meetings<br><br>• Training logs of expected and actual	<u>Meetings</u><br>* Whether the 10 and 2-week warm-up meetings took place/were attended;<br>* Whether the 2 and 4 week post training meetings took place/were attended<br><br><u>Training logs</u>

Implementation outcome (abbreviated definition)	Strategies used to maximise implementation outcomes	Level of analysis	Frameworks used	Data collection instruments, timing, participants	Items from instruments
	• clear plans and materials for content of training			attendance at training of leaders and clinicians recorded at the time of training<br>• Fidelity record (a checklist of which aspects of the planned training were actually delivered – completed by researchers observing the training, plus any additional observational data about engagement of participants)<br>• 6 months following training - semi-structured interview with leaders	* Proportion of eligible leaders and clinicians who were invited and who actually attended training<br><br>Fidelity record<br>* the extent to which training was delivered as anticipated<br><br>6 month interview guide for leaders<br>* *Were any adaptations made to MOHMQuit? (What/who/when/why/how?)*
Penetration (degree of integration of MOHMQuit practices within the service)	• Involving leaders in the training for clinicians for a whole-of-service approach<br>• MOHMQuit leadership components which focus on repeated audit and feedback plus action planning;	Site level	Proctor[1]<br>RE-AIM[2]<br>(Adoption)	• 3 months following training - questionnaire for leaders<br>• 6 months following training - semi-structured interview with leaders	3 month questionnaire for leaders<br>* *In the last 3 months did you or any other staff in your service…* (followed by a number of MOHMQuit components)*?*<br>6 month interview guide for leaders<br>* *Have you, or any of your colleagues implemented any of the components of MOHMQuit* (followed by a number of MOHMQuit components)*?*<br>* *How much has MOHMQuit become part of 'usual practice'? (e.g. standard operating procedures, local policies, SCS as a*

Implementation outcome (abbreviated definition)	Strategies used to maximise implementation outcomes	Level of analysis	Frameworks used	Data collection instruments, timing, participants	Items from instruments
	developing and implementing a clinical pathway for SCS; and the development and maintenance of SCS 'champions' within each service<br>• The train the trainer model as an integral part of the intervention to support its ongoing implementation				*standing item on meeting agendas, audit part of usual audit schedule etc.)*
Reach (did MOHMQuit include all clinicians and leaders that it aimed to?)	• 10-week warm-up meetings to allow time for planning and rostering<br>• The train the trainer model as an integral part of the intervention to support participation of all relevant existing and new staff	Site level	RE-AIM[4]	• Training logs of expected and actual attendance at training of leaders and clinicians recorded at the time of training<br>• 3 months following training - questionnaire for leaders<br>• 6 months following training - semi-	<u>Training logs</u><br>* Proportion of eligible leaders and clinicians who were invited and proportion who actually attended training (compare the seniority, and role e.g. midwife, obstetrician of those who participated to those who did not)<br><u>3 month questionnaire for leaders</u><br>* *In the last 3 months did you or any other staff in your service design and run any staff training on SCS?* (the train the trainer model);<br>* *Please tell us more about this training* (space to write a qualitative response)<br><u>6 month interview guide for leaders</u>

For peer review only

Implementation outcome (abbreviated definition)	Strategies used to maximise implementation outcomes	Level of analysis	Frameworks used	Data collection instruments, timing, participants	Items from instruments
				structured interview with leaders	* *Have you, or any of your colleagues implemented any of the components of MOHMQuit* (followed by a number of MOHMQuit components including *Designing and running any staff training*)?
**Sustainability (whether factors are in place to promote the ongoing use of MOHMQuit)**	• **MOHMQuit leadership components which focus on repeated audit and feedback plus action planning; developing and implementing a clinical pathway for SCS; and the development and maintenance of SCS 'champions' within each service**<br>• **The train the trainer model as an integral part of the intervention to support its ongoing implementation**<br>• **The Community of Practice**	**Site level Individual clinician level**	**Proctor[1] RE-AIM[2] (Maintenance) Rogers[5]**	• **6 months following training - questionnaire with clinicians**<br>• **6 months following training - semi-structured interview with leaders**<br>• **Community of Practice peer support attendance data**	**6 month questionnaire for clinicians**<br>**** How useful were each of the MOHMQuit resources when working with women (scale of 1-3 Very useful to Not at all useful + Not Applicable as a response option)?***<br>**6 month interview guide for leaders**<br>**** How much has MOHMQuit become part of 'usual practice'?***<br>**** What do you think helped in delivering MOHMQuit? (contextual factors)***<br>**Community of Practice meetings**<br>*** Sites attending community of practice meetings**
Acceptability (how palatable is MOHMQuit to	• Comprehensive and systematic design of MOHMQuit using the	Site level Individual level	Proctor[1] Sekhon[8]	• Immediately following training - questionnaire with clinicians	Immediately following training for clinicians:<br>* *On a scale of 1 to 3 (very useful to not at all useful) what's your impression of how useful the MOHMQuit training is going*

For peer review only

Implementation outcome (abbreviated definition)	Strategies used to maximise implementation outcomes	Level of analysis	Frameworks used	Data collection instruments, timing, participants	Items from instruments
clinicians and leaders?)	Behaviour Change Wheel integrating input from clinicians and leaders[6 7]<br>• Feasibility and acceptability trial with subsequent minor amendments to the intervention[6]<br>• At the 10 week warm-up the long 'history' of the development of MOHMQuit is described to ensure leaders are reassured about its quality, relevance and acceptability			• 3 months following training - questionnaire for leaders<br>• 6 months following training - questionnaire with clinicians<br>• 6 months following training - semi-structured interview with leaders	*to be in helping you provide SCS by addressing gaps in your knowledge/skills/confidence?* (perceived effectiveness[8]);<br>* *On a scale of 1-3 (very much* to *not at all) how much do you think MOHMQuit will help you provide SCS* (perceived effectiveness[8]);<br>**Overall how do you feel about MOHMQuit (scale of 1-4)?* (affective attitude[8])<br>3 month questionnaire for leaders:<br>* *Please give MOHMQuit a score of whether you think it has helped your service to routinely provide evidence-based SCS (scale of 1-10 Has not helped at all* to *Has been entirely helpful)* (perceived effectiveness[8]);<br>6 month questionnaire for clinicians:<br>* *On a scale of 1-5 (Strongly agree* to *Strongly disagree) I am confident providing smoking cessation assistance to pregnant women* (self-efficacy[8]);<br>* *On a scale of 1-5 (Strongly agree* to *Strongly disagree) I am confident arranging follow up support for pregnant smokers* (self-efficacy[8]);<br>* *On a scale of 1-4* (very much to not at all) *to what extent did MOHMQuit help you to provide high quality smoking cessation support to women at every visit?* (perceived effectiveness[8])<br>6 month interview guide for leaders<br>* *How would you describe MOHMQuit (what it is and how it aims to improve practice) to a leader in a maternity service in a different hospital?* (intervention coherence[8])<br>* *Did MOHMQuit improve the SCS provided to pregnant women in your service?* (perceived effectiveness[8])?

For peer review only

Implementation outcome (abbreviated definition)	Strategies used to maximise implementation outcomes	Level of analysis	Frameworks used	Data collection instruments, timing, participants	Items from instruments
Appropriateness (perceived fit or relevance of MOHMQuit with the service)	• Comprehensive and systematic design of MOHMQuit using the Behaviour Change Wheel integrating input from clinicians and leaders[6 7]<br>• Feasibility and acceptability trial with subsequent minor amendments to the intervention[6]<br>• At the 10 week warm-up the long 'history' of the development of MOHMQuit is described to ensure leaders are reassured about its quality, relevance and acceptability	Site level	Proctor[1]<br>Rogers[5]	• 6 months following training - semi-structured interview with leaders	6 month interview guide for leaders<br>* *Were any adaptations made to MOHMQuit? (What/who/when/why/how?)*
Feasibility (actual fit – the extent to which MOHMQuit can be integrated into usual care in a service)	• Comprehensive and systematic design of MOHMQuit using the Behaviour Change Wheel integrating input from clinicians and leaders[6 7]	Site level	Proctor[1]<br>Rogers[5]	• 3 months following training - questionnaire for leaders<br>• 6 months following training - semi-structured interview with leaders	3 month questionnaire for leaders<br>* *In the last 3 months did you or any other staff in your service…* (followed by a number of MOHMQuit components)*?*<br>6 month interview guide for leaders<br>* *Have you, or any of your colleagues implemented any of the components of MOHMQuit* (followed by a number of MOHMQuit components)*?*

For peer review only

Implementation outcome (abbreviated definition)	Strategies used to maximise implementation outcomes	Level of analysis	Frameworks used	Data collection instruments, timing, participants	Items from instruments
	• Feasibility and acceptability trial with subsequent minor amendments to the intervention[6]<br>• At the 10 week warm-up the long 'history' of the development of MOHMQuit is described to ensure leaders are reassured about its quality, relevance and acceptability				* *How much has MOHMQuit become part of 'usual practice'? (e.g. standard operating procedures, local policies, SCS as a standing item on meeting agendas, audit part of usual audit schedule etc.)*
HOW the implementation of the intervention changed behaviour – the 'mechanisms of impact'+ acceptability, appropriateness and feasibility above		Site level	Moore[4]	• 6 months following training - semi-structured interview with leaders	<u>6 month interview guide for leaders</u><br>* *Did MOHMQuit improve the SCS provided to pregnant women in your service? How did it do this?*<br>* *How can the implementation of MOHMQuit be improved?*
**HOW context affected implementation**	• **Commitment of maternity service leaders in the**	**Site level**	**Fernandez[9]**	• **Key contextual information (Table 1) completed by research**	**<u>See Table 1 above</u>**<br>* **Birth numbers; smoking prevalence; Performance against the performance indicator of antenatal smoking; Safer Baby**

For peer review only - http://bmjopen.bmj.com/site/about/guidelines.xhtml

Implementation outcome (abbreviated definition)	Strategies used to maximise implementation outcomes	Level of analysis	Frameworks used	Data collection instruments, timing, participants	Items from instruments
	research as Partner Investigators on the grant and as members of the MOHMQuit research Steering Committee and various working groups<br>• Warm-up meetings<br>• Follow up meetings<br>• Community of Practice			team during the implementation<br>• 3 months following training - questionnaire for leaders<br>• 6 months following training - questionnaire with clinicians<br>• 6 months following training - semi-structured interview with leaders	Bundle; RSVP policy; Other SCS initiatives; Accreditation; leadership structure; models of care on offer; other<br><br><u>3 month questionnaire for leaders</u><br>* *Please indicate the extent to which you agree (from Not at all* to *Very great extent)…* all 12 items from the Implementation Leadership Scale[10] e.g. *I have developed a plan to facilitate the implementation of MOHMQuit*<br><u>6 month questionnaire for clinicians</u><br>* *How well do you feel your service leadership has supported the implementation of MOHMQuit* (scale of 1-5 from *Strongly Disagree* to *Strongly* agree) the 4 items of the Implementation Climate measure[9] *e.g. Our service leadership makes sure that we have the time and space necessary to discuss changes to improve care*<br>* *…the general feeling for implementation of MOHMQuit in your service* (scale of 1-5 from *Strongly Disagree* to *Strongly* agree) the 4 items from the Leadership Engagement measure[9] *e.g. Our service staff get the support they need to implement MOHMQuit*<br><u>6 month interview guide for leaders</u><br>* *Has anything changed in terms of your or others' leadership within the service/s around SCS due to MOHMQuit? (Why? How?)*<br>* *What do you think helped in delivering MOHMQuit?*<br>* *What made delivering MOHMQuit more of a challenge?*

BOLD TYPEFACE INDICATES OUTCOMES THAT WILL BE THE FOCUS OF THE PROCESS EVALUATION

Implementation cost is not included in Table 2 as a detailed economic evaluation of MOHMQuit is taking place and is the subject of a separate paper.[11] Data to contribute to the economic evaluation will be collected as part of the semi-structured interview with leaders.

References

1. Proctor E, Silmere H, Raghavan R, et al. Outcomes for implementation research: conceptual distinctions, measurement challenges, and research agenda. *Administration and policy in mental health and mental health services research* 2011;38(2):65-76.
2. Glasgow RE, Vogt TM, Boles SM. Evaluating the public health impact of health promotion interventions: the RE-AIM framework. *Am J Public Health* 1999;89(9):1322-27.
3. Barnes LAJ, Longman J, Adams C, et al. The MOHMQuit (Midwives and Obstetricians Helping Mothers to Quit Smoking) Trial: protocol for a stepped-wedge implementation trial to improve best practice smoking cessation support in public antenatal care services. *Implement Sci* 2022;17(1):79. doi: 10.1186/s13012-022-01250-3
4. Moore GF, Audrey S, Barker M, et al. Process evaluation of complex interventions: Medical Research Council guidance. *bmj* 2015;350
5. Rogers EM. Diffusion of Innovations. 4th ed. New York: The Free Press 1995.
6. Passey ME, Adams C, Paul C, et al. Improving implementation of smoking cessation guidelines in pregnancy care: Development of an intervention to address system, maternity service leader and clinician factors. *Implementation Science Communications* 2021;2:128. doi: https://doi.org/10.1186/s43058-021-00235-5
7. Longman J, Adams C, Paul C, et al. Improving clinicians' implementation of guidelines to help women stop smoking in pregnancy: developing evidence-based print and video materials. *Int J Environ Res Public Health* 2021;18(19):10522. doi: https://doi.org/10.3390/ijerph181910522
8. Sekhon M, Cartwright M, Francis JJ. Acceptability of healthcare interventions: an overview of reviews and development of a theoretical framework. *BMC Health Serv Res* 2017;17(1):88.
9. Fernandez ME, Walker TJ, Weiner BJ, et al. Developing measures to assess constructs from the Inner Setting domain of the Consolidated Framework for Implementation Research. *Implement Sci* 2018;13(1):52. doi: 10.1186/s13012-018-0736-7
10. Aarons GA, Ehrhart MG, Farahnak LR. The Implementation Leadership Scale (ILS): Development of a Brief Measure of Unit Level Implementation Leadership. *Implement Sci* 2014;9:45. doi: 10.1186/1748-5908-9-45
11. Pearce A, Scarfe J, Jones M, et al. Study protocol of an economic evaluation embedded in the Midwives and Obstetricians Helping Mothers to Quit Smoking (MOHMQuit) trial. *BMC Health Serv Res* 2023;23(1):1-9.

For peer review only

SPIRIT

STANDARD PROTOCOL ITEMS: RECOMMENDATIONS FOR INTERVENTIONAL TRIALS

SPIRIT 2013 Checklist: Recommended items to address in a clinical trial protocol and related documents*

Section/item	Item No	Description
**Administrative information**		
Title	1	Descriptive title identifying the study design, population, interventions, and, if applicable, trial acronym<br>***Protocol for the process evaluation of an intervention to improve antenatal smoking cessation support (MOHMQuit) in maternity services in New South Wales, Australia***
Trial registration	2a	Trial identifier and registry name. If not yet registered, name of intended registry<br>***The MOHMQuit trial is registered (ACTRN12622000167763 https://www.australianclinicaltrials.gov.au/anzctr/trial/ACTRN12622000167763)***
	2b	All items from the World Health Organization Trial Registration Data Set<br>**N/A**
Protocol version	3	Date and version identifier<br>**The protocol for the whole MOHMQuit trial (which includes the process evaluation) is a published paper (Barnes, L. A. J., J. Longman, C. Adams, C. Paul, L. Atkins, B. Bonevski, A. Cashmore, L. Twyman, R. Bailie, A. Pearce, D. Barker, A. J. Milat, J. Dorling, M. Nicholl and M. Passey (2022). "The MOHMQuit (Midwives and Obstetricians Helping Mothers to Quit Smoking) Trial: protocol for a stepped-wedge implementation trial to improve best practice smoking cessation support in public antenatal care services." Implementation Science 17(1): 79). This paper itself is a more detailed protocol for the process evaluation.**
Funding	4	Sources and types of financial, material, and other support<br>***This work was supported by funding from the NHMRC (GNT1072213) and the Cancer Institute NSW (13/ECF/1-11). MP was supported by a fellowship from the NHMRC (GNT1159601).***

For peer review only - http://bmjopen.bmj.com/site/about/guidelines.xhtml

Roles and responsibilities	5a	Names, affiliations, and roles of protocol contributors<br>***Jo Longman[1], Chris Paul[2], Aaron Cashmore[3,4], Laura Twyman[5], Larisa AJ Barnes[1], Cathy Adams[6], Billie Bonevski[7], Andrew Milat[3,4] and Megan E Passey[1]* (affiliations are listed on the title page of the paper).**<br>***The process evaluation was conceived and designed by MP, JL, CP, LT, LB, CA, BB and LA. The first draft of the paper was written by JL with input from MP and CP before receiving input from all other authors. All authors have read and approved the final manuscript.***
	5b	Name and contact information for the trial sponsor<br>N/A
	5c	Role of study sponsor and funders, if any, in study design; collection, management, analysis, and interpretation of data; writing of the report; and the decision to submit the report for publication, including whether they will have ultimate authority over any of these activities<br>**The funding bodies did not have any role in the design of the study and collection, analysis and interpretation of data.**
	5d	Composition, roles, and responsibilities of the coordinating centre, steering committee, endpoint adjudication committee, data management team, and other individuals or groups overseeing the trial, if applicable (see Item 21a for data monitoring committee)<br>N/A
**Introduction**		
Background and rationale	6a	Description of research question and justification for undertaking the trial, including summary of relevant studies (published and unpublished) examining benefits and harms for each intervention<br>**Includes research questions driving the process evaluation and justification for the process evaluation – see paragraph *"Aims of the MOHMQuit process evaluation"*.**
	6b	Explanation for choice of comparators<br>N/A
Objectives	7	Specific objectives or hypotheses<br>**See paragraph *"Overall design and objectives of the process evaluation"*.**

For peer review only

Trial design	8	Description of trial design including type of trial (eg, parallel group, crossover, factorial, single group), allocation ratio, and framework (eg, superiority, equivalence, noninferiority, exploratory)<br>***MOHMQuit is currently being trialled in a multi-site cluster randomised stepped-wedge effectiveness trial in nine sites in publicly-funded maternity services in NSW, Australia***

Methods: Participants, interventions, and outcomes

Study setting	9	Description of study settings (eg, community clinic, academic hospital) and list of countries where data will be collected. Reference to where list of study sites can be obtained<br>**MOHMQuit is currently being trialled in a multi-site cluster randomised stepped-wedge effectiveness trial in nine sites in publicly-funded maternity services in NSW, Australia**.
Eligibility criteria	10	Inclusion and exclusion criteria for participants. If applicable, eligibility criteria for study centres and individuals who will perform the interventions (eg, surgeons, psychotherapists)<br>**See "Recruitment and Consent" section**
Interventions	11a	Interventions for each group with sufficient detail to allow replication, including how and when they will be administered<br>**The MOHMQuit intervention has been described in detail in a previously published manuscript so is described in brief here. See "The MOHMQuit Intervention" section**
	11b	Criteria for discontinuing or modifying allocated interventions for a given trial participant (eg, drug dose change in response to harms, participant request, or improving/worsening disease)<br>N/A
	11c	Strategies to improve adherence to intervention protocols, and any procedures for monitoring adherence (eg, drug tablet return, laboratory tests)<br>N/A
	11d	Relevant concomitant care and interventions that are permitted or prohibited during the trial<br>N/A

For peer review only - http://bmjopen.bmj.com/site/about/guidelines.xhtml

Outcomes	12	Primary, secondary, and other outcomes, including the specific measurement variable (eg, systolic blood pressure), analysis metric (eg, change from baseline, final value, time to event), method of aggregation (eg, median, proportion), and time point for each outcome. Explanation of the clinical relevance of chosen efficacy and harm outcomes is strongly recommended<br>**Primary and secondary intervention outcomes are described in brief:** ***The primary intervention outcome is smoking cessation, and secondary intervention outcomes include changes to clinicians' knowledge, skills, confidence and behaviour in providing SCS and test the 'mechanisms of action' by which each of the components/strategies effect intervention outcomes and moderators of their impact in this framework-driven approach. Cost-effectiveness will be assessed in an economic evaluation.*** **The implementation outcomes (the process evaluation) are described in detail – see "Overall design and objectives of the process evaluation" section and Table 4**
Participant timeline	13	Time schedule of enrolment, interventions (including any run-ins and washouts), assessments, and visits for participants. A schematic diagram is highly recommended (see Figure)<br>**The timeline for the process evaluation is described in the "Trial status" paragraph**.
Sample size	14	Estimated number of participants needed to achieve study objectives and how it was determined, including clinical and statistical assumptions supporting any sample size calculations<br>N/A
Recruitment	15	Strategies for achieving adequate participant enrolment to reach target sample size<br>N/A

Methods: Assignment of interventions (for controlled trials)

Allocation:

Sequence generation	16a	Method of generating the allocation sequence (eg, computer-generated random numbers), and list of any factors for stratification. To reduce predictability of a random sequence, details of any planned restriction (eg, blocking) should be provided in a separate document that is unavailable to those who enrol participants or assign interventions<br>N/A
Allocation concealment mechanism	16b	Mechanism of implementing the allocation sequence (eg, central telephone; sequentially numbered, opaque, sealed envelopes), describing any steps to conceal the sequence until interventions are assigned<br>N/A

Implementation	16c	Who will generate the allocation sequence, who will enrol participants, and who will assign participants to interventions<br>N/A
Blinding (masking)	17a	Who will be blinded after assignment to interventions (eg, trial participants, care providers, outcome assessors, data analysts), and how<br>N/A
	17b	If blinded, circumstances under which unblinding is permissible, and procedure for revealing a participant's allocated intervention during the trial<br>N/A
**Methods: Data collection, management, and analysis**		
Data collection methods	18a	Plans for assessment and collection of outcome, baseline, and other trial data, including any related processes to promote data quality (eg, duplicate measurements, training of assessors) and a description of study instruments (eg, questionnaires, laboratory tests) along with their reliability and validity, if known. Reference to where data collection forms can be found, if not in the protocol<br>**This is described in the "Process evaluation data collection" section**
	18b	Plans to promote participant retention and complete follow-up, including list of any outcome data to be collected for participants who discontinue or deviate from intervention protocols<br>**Plans to keep MOHMQuit at the forefront of clinicians' and leaders' minds (from whom data will be collected 6 months following the intervention) include those addressing sustainability of the intervention: MOHMQuit leadership components; the 'train the trainer' model; and the Community of Practice (Table 4)**
Data management	19	Plans for data entry, coding, security, and storage, including any related processes to promote data quality (eg, double data entry; range checks for data values). Reference to where details of data management procedures can be found, if not in the protocol<br>**This detail is included in the ethics application for the trial (which includes the process evaluation) and for the sake of brevity are not included in this manuscript.**
Statistical methods	20a	Statistical methods for analysing primary and secondary outcomes. Reference to where other details of the statistical analysis plan can be found, if not in the protocol<br>**This is described in the "Data analysis" section**

	20b	Methods for any additional analyses (eg, subgroup and adjusted analyses)<br>N/A
	20c	Definition of analysis population relating to protocol non-adherence (eg, as randomised analysis), and any statistical methods to handle missing data (eg, multiple imputation)<br>N/A
**Methods: Monitoring**		
Data monitoring	21a	Composition of data monitoring committee (DMC); summary of its role and reporting structure; statement of whether it is independent from the sponsor and competing interests; and reference to where further details about its charter can be found, if not in the protocol. Alternatively, an explanation of why a DMC is not needed<br>N/A
	21b	Description of any interim analyses and stopping guidelines, including who will have access to these interim results and make the final decision to terminate the trial<br>N/A
Harms	22	Plans for collecting, assessing, reporting, and managing solicited and spontaneously reported adverse events and other unintended effects of trial interventions or trial conduct<br>N/A
Auditing	23	Frequency and procedures for auditing trial conduct, if any, and whether the process will be independent from investigators and the sponsor<br>N/A
**Ethics and dissemination**		
Research ethics approval	24	Plans for seeking research ethics committee/institutional review board (REC/IRB) approval<br>***Ethics approval for the research was received from the Population Health Services Research Ethics Committee (Reference Number 2021/ETH00887), on July 23rd, 2021*.**
Protocol amendments	25	Plans for communicating important protocol modifications (eg, changes to eligibility criteria, outcomes, analyses) to relevant parties (eg, investigators, REC/IRBs, trial participants, trial registries, journals, regulators)<br>N/A
Consent or assent	26a	Who will obtain informed consent or assent from potential trial participants or authorised surrogates, and how (see Item 32)<br>**This is described in the section "Ethical approval and consent to participate"**

For peer review only - http://bmjopen.bmj.com/site/about/guidelines.xhtml

	26b	Additional consent provisions for collection and use of participant data and biological specimens in ancillary studies, if applicable<br>N/A
Confidentiality	27	How personal information about potential and enrolled participants will be collected, shared, and maintained in order to protect confidentiality before, during, and after the trial<br>**This is described in the section "Ethical approval and consent to participate"**
Declaration of interests	28	Financial and other competing interests for principal investigators for the overall trial and each study site<br>**There is a "Competing Interests" statement and a ICMJE Disclosure Form submitted with the manuscript**
Access to data	29	Statement of who will have access to the final trial dataset, and disclosure of contractual agreements that limit such access for investigators<br>N/A
Ancillary and post-trial care	30	Provisions, if any, for ancillary and post-trial care, and for compensation to those who suffer harm from trial participation<br>N/A
Dissemination policy	31a	Plans for investigators and sponsor to communicate trial results to participants, healthcare professionals, the public, and other relevant groups (eg, via publication, reporting in results databases, or other data sharing arrangements), including any publication restrictions<br>**This is described in the "Ethics and dissemination" section**
	31b	Authorship eligibility guidelines and any intended use of professional writers<br>**This level of detail has not been included for the sake of brevity. Professional writers will not be used.**
	31c	Plans, if any, for granting public access to the full protocol, participant-level dataset, and statistical code<br>N/A
**Appendices**		
Informed consent materials	32	Model consent form and other related documentation given to participants and authorised surrogates<br>**This level of detail has not been included for the sake of brevity.**
Biological specimens	33	Plans for collection, laboratory evaluation, and storage of biological specimens for genetic or molecular analysis in the current trial and for future use in ancillary studies, if applicable<br>N/A

*It is strongly recommended that this checklist be read in conjunction with the SPIRIT 2013 Explanation & Elaboration for important clarification on the items. Amendments to the

For peer review only - http://bmjopen.bmj.com/site/about/guidelines.xhtml

protocol should be tracked and dated. The SPIRIT checklist is copyrighted by the SPIRIT Group under the Creative Commons "Attribution-NonCommercial-NoDerivs 3.0 Unported" license.

For peer review only

For peer review only - http://bmjopen.bmj.com/site/about/guidelines.xhtml
